# Fluorine-Induced Rigidity and Entropy Effects in Mixed-Halide 2,2-Difluoroethylammonium Cadmium Hybrids

**DOI:** 10.1021/acs.inorgchem.6c01320

**Published:** 2026-06-10

**Authors:** Maciej Ptak, Dorota A. Kowalska, Szymon Smółka, Edyta Kucharska, Mariusz Stefanski, Damian Szymański, Anna Ładak, Adam Sieradzki

**Affiliations:** † Institute of Low Temperature and Structure Research, Polish Academy of Sciences, 50-422 Wrocław, Poland; ‡ Department of Bioorganic Chemistry, Wroclaw University of Economics and Business, 53-345 Wrocław, Poland; § Department of Experimental Physics, 49567 Wrocław University of Science and Technology, 50-370 Wrocław, Poland

## Abstract

We report a comprehensive study of structure-related properties in difluorinated 2D cadmium halides (F_2_EA)_2_CdX_4_ (X = Cl^–^, Br^–^, and mixed Br/Cl ratio of 60:40). Structural single-crystal X-ray diffraction studies demonstrated that the halide substitution introduces positional disorder, affecting the organic cations and the inorganic layers of corner-sharing octahedra. Differential scanning calorimetry showed large entropy changes related to the phase transition, largely exceeding the values expected for the order–disorder processes, indicating a complex mechanism involving the ordering of cations, hydrogen bond reorganization, and distortion of inorganic sublattices. Structural studies, together with IR spectroscopy experiments supported by DFT calculations, demonstrated that fluorination enhances the intermolecular F···F interactions, leading to unusually short contacts and increasing the rigidity of the hybrid. The noncovalent interaction calculations confirm that the short F···F contacts are not associated with stabilizing interactions but rather with sterically enforced packing. The phase transition is accompanied by significant changes in molecular dynamics and the tilting of inorganic slabs. Optical studies indicated that tunable emission is governed by a halide substitution and a change in the structural rigidity resulting from hydrogen bonds, accompanied by the F···F interactions. These findings highlight the important role of multiple fluorination and halide mixing in controlling structural disorder, lattice dynamics, and optoelectronic response in layered hybrid crystals.

## Graphical abstract

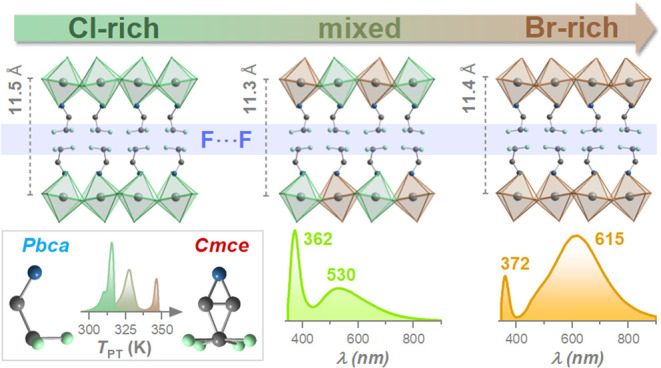

## Introduction

Hybrid organic–inorganic halides are attracting increasing attention from scientists due to their multifunctionality, which includes good structural, thermal, electric, magnetic, phonon, optical, and other properties.^1–6^ Among the vast variety of compounds, cadmium-based hybrid halides are gaining attention due to their strong tendency to form flexible two-dimensional (2D) structures. The 2D crystal structures with a general formula of A_2_CdX_4_ (X = Cl^–^, Br^–^, I^–^) can accommodate either small or more complex ammonium cations A^+^, so the interlayer distance can be easily fine-tuned during the synthesis.^7–11^

Their optoelectronic properties vary from those of common Pb^2+^ hybrids because Cd^2+^ ions do not have stereochemically active lone pairs, making their halide octahedra less prone to tilting and distortion. Differences in the crystal lattice rigidity result in different photoluminescence responses. In cadmium-based halides, the optical behavior is influenced not only by the softness of the inorganic layer but also by the dynamics of organic cations and the hydrogen-bonding (HB) network, which govern the ability of the crystal lattice to undergo deformations. This makes the Cd^2+^-based systems particularly sensitive to coupled organic–inorganic lattice dynamics.

One example of modifying the chemical properties of hybrid cadmium halides is the halogenation of organic cations.^5,12–16^ The smallest aliphatic ammonium cation that has a stable halogenated form at room temperature (RT) is the ethylammonium cation (EA^+^). It has been demonstrated in the literature that fluorination is very promising, as it may cause a strong increase in the dipole moment of the cation and, through an inductive effect, increase the strength of HBs created by the protonated amino group.^13–15^ Changes in interactions within the crystal structure modify the mechanisms of phase transitions, molecular dynamics, and fundamental properties. In extreme cases, it can even induce noncentrosymmetry, which is an important factor for future applications involving nonlinear optical properties or triggering of ferroelectric polarization.^13,14,17,18^ Furthermore, fluorination may cause the increase of mechanical properties, as observed for FEA_2_[KFe­(CN)_6_] (FEA^+^ = 2-fluoroethylammonium),^19^ or regulate the stability and hydrophobicity.^17,20^ Most studies focus on lead hybrid organic–inorganic halides.^17,21^

To better understand the influence of F atoms in the organic cation on the properties, we have recently performed studies of FEA^+^-composed cadmium chlorobromides, demonstrating that fluorination has an important role in the order–disorder mechanism of phase transition (PT) characterized by an unusually high change in entropy.^5,12^ Continuing, in this work, we present similar studies for analogous chloride, bromide, and mixed chlorobromide (∼63 mol % Br) compounds containing two F atoms in EA^+^, namely, 2,2-difluoroethylammonium cation (F_2_EA^+^). This modification causes unusually short intermolecular F···F contacts to appear in the crystal structure, from 2.55 to 2.85 Å in the low-temperature (LT) predominantly ordered phase to 2.45–2.74 Å in the high-temperature (HT) dynamically disordered phase.

The existence of the F···F halogen bond is still disputed because the C–F bond is hardly polarizable, and most of the distances reported to date were slightly lower than the sum of the van der Waals radii of 2.94 Å.^22,23^ Such a short distance is not associated with attractive halogen forces but rather results from the repulsive forces, as in most reported cases, is explained just by a dense packing type.^22–25^ It has been reported that fluorine can develop a σ-hole and potentially engage in halogen bonding interactions when bonded to strongly electron-withdrawing moieties. However, such interactions are generally too weak to be stabilized in the presence of competing intermolecular forces, for example, HBs.^26^ More recently, Scheiner has demonstrated using density functional theory (DFT) calculations that the presence of a σ-hole alone is not sufficient evidence for a stabilizing interaction and must be supported by energetic analysis to confirm its attractive nature. Furthermore, it has been shown that even short F···F contacts, shorter than the sum of van der Waals radii and displaying geometries typically associated with halogen bonding, are unlikely to correspond to true halogen bonds and are instead governed predominantly by dispersion forces.^27^

In line with the ongoing discourse, it is important to examine materials with short F···F contacts as representative model cases. A systematic study of F···F interactions is essential for understanding structure–property relationships in hybrid halide crystals, particularly when multiple intermolecular interactions coexist and collectively determine the crystal packing and resulting physical properties.

The research performed here on PT mechanisms makes a significant contribution to the state of knowledge in understanding fluorination-induced processes and changes of ligands in 2D hybrids. In addition, the effect of ligand substitution on the structural, phonon, thermal, and optical (absorption and emission) properties was analyzed.

## Materials and Methods

### Materials and Synthesis

The following chemicals were purchased and used without additional purification: 2,2-difluoroethylammonium chloride (95%, Sigma-Aldrich), cadmium chloride (99%, Sigma-Aldrich), cadmium bromide tetrahydrate (98%, Thermo Fisher Scientific), hydrobromic acid (48%, Sigma-Aldrich), hydrochloric acid (33%, Avantor), acetonitrile (99.5%, Sigma-Aldrich), and methanol (99.8%, Sigma-Aldrich).

To grow (F_2_EA)_2_CdCl_4_ (**F**_**2**_ **EACdC**) and (F_2_EA)_2_CdBr_4_ (**F**_**2**_ **EACdB**) crystals, 1 mmol of 2,2-difluoroethylammonium chloride (0.1175 g) and 0.5 mmol of cadmium chloride (0.0916 g) were dissolved in a mixture of 2.5 mL of methanol, 2.5 mL of acetonitrile, and 1.25 mL of concentrated acid. In the synthesis of (F_2_EA)_2_Cd­[Br_0.6_Cl_0.4_]_4_ (**F**_**2**_ **EACdCB**), a mixture of 0.25 mmol of CdCl_2_ (0.0458 g) + CdBr_2_·4H_2_O (0.0861 g) and 0.75 mL of hydrochloric acid + 0.75 mL of hydrobromic acid was used. After a few days, white plate-shaped crystals were separated and rinsed with cold methanol.

### Differential Scanning Calorimetry (DSC)

The heat-flow thermogram was measured by using a high-resolution 0.04 μW Mettler Toledo DSC-3 calorimeter and nitrogen as a purging gas. The experiments were carried out in the 150 and 340 or 380 K temperature range, using a heating/cooling rate of 5 K min^–1^. The excess heat capacities associated with PTs were determined by removing the baseline in the absence of the PTs.

### Single-Crystal X-ray Diffraction (sc-XRD)

An Oxford X’Calibur four-circle diffractometer equipped with a CCD Atlas detector and graphite-monochromated Mo Kα radiation (λ = 0.71073 Å) was used for single-crystal X-ray diffraction measurements. An Oxford Cryosystems Cryostream1000 Plus cryocooler was used for temperature control under non-ambient conditions. Diffraction intensities were collected and processed using the CrysAlis PRO software suite,^28^ and absorption effects were corrected numerically by Gaussian integration over a multifaceted crystal model. For each sample, diffraction data in both phases were obtained from the same crystal specimen.

The structures were solved by direct methods and subsequently refined by full-matrix least-squares procedures on *F* ^2^ with the SHELX software package^29,30^ as implemented in Olex^2^.^31^ All non-hydrogen atoms were refined anisotropically, whereas hydrogen atoms were treated using constrained models, except in the LT of **F**_**2**_ **EACdBC**, where a combination of independent and constrained refinement was employed. The Br^–^/Cl^–^ site occupancies in **F**_**2**_ **EACdBC** were refined independently for each site and phase, giving an overall Br/Cl ratio of 62:38. Structural illustrations were generated with Diamond 3.2k.^32^ Hirshfeld surface analysis of the F_2_EA^+^ cation and corresponding fingerprint plots were prepared using CrystalExplorer.^33^

### Powder X-ray Diffraction (p-XRD)

Powder X-ray diffraction patterns were measured using an X’Pert PRO X-ray diffractometer equipped with a PIXcel ultrafast detector and Soller slits for Cu Kα radiation (λ = 1.54158 Å). The patterns were measured as reflections with the X-ray tube set at 30 mA and 40 kV.

### Infrared Spectra (IR)

The temperature-dependent mid-IR spectra (3500–400 cm^–1^), in the 10–320 K range and 10 K steps, were collected using a Nicolet iS50 IR spectrometer (Thermo Scientific) combined with a CS202AE-DMX-1AL closed-cycle helium cryostat (Advanced Research Systems) equipped with KRS-5 windows. The corresponding mid-IR spectra above RT (300–400 K, 10 K step) were registered in the 3500–600 cm^–1^ spectral range using a Nicolet iN10 stand-alone IR microscope (Thermo Scientific) combined with an FTIR600 stage (Linkam) equipped with ZnSe windows. All IR measurements were performed on KBr pellets with a spectral resolution of 2 cm^–1^.

### Raman Spectra

Room-temperature Raman spectra were measured using an FT MultiRam spectrometer (Bruker) with an excitation at 1064 nm (YAG:Nd); the spectral resolution was set to 2 cm^–1^.

### Absorption Spectra

The diffuse reflection spectra (DRS) of small single crystals in the UV–vis-NIR range were collected in the backscattering geometry using an Agilent Cary 5000 spectrophotometer. The Al_2_O_3_ powder was used as a reference.

### Emission Spectra and Decay Times

Temperature-dependent emission spectra were measured with a Hamamatsu photonic multichannel analyzer PMA-12 equipped with a BT-CCD linear image sensor. A laser diode operating at 266 nm was used as an excitation source. The Linkam THMS600 heating/freezing stage controlled the temperature of the samples during emission measurements. The luminescence decay profile was recorded using a femtosecond laser (Coherent Model Libra).

### Energy-Dispersive X-ray Spectroscopy (EDS)

The chemical composition of **F**_**2**_ **EACdBC** was investigated by using an FE-SEM FEI NovaNano SEM 230 (FEI Company as a part of Thermo Fisher Scientific) equipped with an energy-dispersive X-ray spectrometer EDAX Genesis XM4 with a resolution better than 135 eV and compatible with Genesis EDAX Microanalysis software. In the first step, the samples were placed on the stub to eliminate the charging and drift problems. After that, the samples were put under a microscope and analyzed by using secondary electron (SE) and the following settings: an acceleration voltage of 30 kV and a chamber pressure equal to 0.5 mbar. The EDS analysis was performed in three randomly selected areas to obtain satisfactory statistical averaging.

### Quantum Chemical Calculations

The geometry optimization of the molecular structures of the 2,2-difluoroethylamine (F_2_EA) molecule and the F_2_EA^+^ cation was performed with the use of the Gaussian 16 program package.^34^ All calculations were performed using density functional three-parameter hybrid (B3LYP) methods^35,36^ with the 6–311G­(2d,2p)^37,38^ basis set starting from the X-ray geometry. The harmonic and anharmonic wavenumbers were also calculated. The calculated harmonic frequencies were scaled using scaling factors (0.96 and 0.98) to correct the evaluated wavenumbers for vibrational anharmonicity and deficiencies inherent to the computational level. The potential energy distribution (PED) of the normal modes among the respective internal coordinates was calculated for the studied compounds using the BALGA program.^39^ The data from DFT calculations were introduced into the BALGA program. The theoretical Raman intensities were calculated by using the Chemcraft program.^40^ This program was also used for the visualization of molecules.

### Noncovalent Interaction (NCI) Analysis

Calculations have been carried out using resources provided by the Wroclaw Centre for Networking and Supercomputing (https://wcss.pl); computing resources via account hpc-1779111527. The ORCA program was used to perform quantum chemical calculations.^41^ Multiwfn was employed to compute the reduced density gradient and analyze noncovalent interactions.^42,43^ VMD was used to visualize the NCI isosurfaces and intermolecular interactions.^44^ RDG (reduced density gradient) plots were prepared using gnuplot software (version 5.4).^45^

## Results and Discussion

### Chemical Composition and Phase Purity

To determine the exact composition of the mixed-halide compound **F**_**2**_ **EACdBC**, we applied the EDS technique (Figure S1a), giving the average results of 55.6 at% of Br and 44.4 at% of Cl elements. The sc-XRD data gave slightly varying values, i.e., 62 at% for Br^–^ and 38 at% for Cl^–^; therefore, the mean composition of **F**_**2**_ **EACdBC** was estimated to (F_2_EA)_2_Cd­[Br_0.59_Cl_0.41_]_4_. The corresponding SEM images show a typical platelike architecture of crystals adopting the 2D structure (Figure S1b–d).

The phase purity of the crystals was validated by powder X-ray diffraction. For **F**_**2**_ **EACdC** and **F**_**2**_ **EACdB**, there is a good match of measured patterns with simulations based on single-crystal refinement (Figure S2). For the mixed-ligand crystal, some additional phase is evidenced but could not be identified. Additional peaks for **F**_**2**_ **EACdBC** may also arise from the partial decomposition or halide segregation of finely ground powders after prolonged exposure to ambient conditions, which may explain the resemblance of the **F**_**2**_ **EACdBC** pattern as a mixture of two end-member phases. This instability was not observed for shorter DRS measurements, those performed on single crystals (sc-XRD, emission, and decay times) or powdered samples stored in inert gas chambers (DSC, Raman, and emission), or those stored in a vacuum (IR and EDS), ensuring that all experiments were performed on phase-pure samples.

### DSC

Figure S3 presents the DSC curves recorded for all three crystals at the thermal rate of 5 K min^–1^. For **F**_**2**_ **EACdC**, two anomalies occur: stronger at *T*_1_ = 315.5/319.8 K; Δ*T* = 4.3 K (cooling/heating) and weaker at *T*_2_ = 310.3/313.1 K; Δ*T* = 2.8 K (see Table 1). Thermal hysteresis for both transitions and the symmetric shape of peaks indicate their first-order nature. This is in line with previously reported data for **F**_**2**_ **EACdC**, showing a phase transition at 317.15/319.29 K; however, the weaker transformation at *T*_2_ has not been analyzed.^12^

**Table 1:** DSC-Derived Thermodynamic Parameters Estimated for the Studied Crystals

compound	*T*_PT_ ^a^ [K]	Δ*T* [K]	Δ*S* ^a^ ^,^ ^b^ [Jmol^–1^ K^–1^]	*N* ^b^
F_2_EACdC	315.5/319.8	4.3	30.4/30.6	38.7/39.7
	310.3/313.1	2.8		
F_2_EACdBC	327.4/330.9	3.5	26.5/27.8	24.2/28.3
F_2_EACdB	346.0/349.3	3.3	29.9/27.4	36.5/27.0

a For cooling/heating run.

b For **F**_**2**_ **EACdC** given as a sum of Δ*S* at *T*_1_ and *T*_2_.

For bromide analogue **F**_**2**_ **EACdB**, only one sharp peak is observed at *T*_1_ = 346.0/349.3 K; Δ*T* = 3.3 K. Both the exothermic and endothermic peaks exhibit asymmetry; however, the existence of thermal hysteresis suggests that this transformation is discontinuous, implying that the asymmetry is linked to the overlap of two closely related phase transitions. For the third analogue with mixed ligands **F**_**2**_ **EACdBC**, the PT is observed at *T*_1_ = 327.4/330.9 K; Δ*T* = 3.5 K, as broader peaks, indicating blurring caused by substitution disorder. The analysis of peak shift and asymmetry leads to similar conclusions as in the case of **F**_**2**_ **EACdB**.

The calculated changes in heat capacity Δ*C*_p_, presented in Figure 1a, and estimated changes in entropy Δ*S* (Figure 1b) associated with phase transitions are rather high, i.e., 30.5, 27.1, and 28.7 Jmol^–1^ K^–1^ on average for **F**_**2**_ **EACdC**, **F**_**2**_ **EACdBC**, and **F**_**2**_ **EACdC**, respectively (see Table 1). Considering that each organic cation F_2_EA^+^ has only two possible orientations in the HT phase (*N* = 2) and is fully ordered below the PT temperature, the expected change in entropy based on the Boltzmann equation Δ*S* = *R *ln *N* (*R* is the gas constant) has a magnitude of 5.8 Jmol^–1^ K^–1^, as depicted in Figure 1b. Such high variability in Δ*S* indicates a complex PT mechanism, which, in addition to cation ordering, also has another very important contribution. Similarly, high values of Δ*S* have been reported for monofluorinated analogues and have been explained by strong displacement of inorganic layers during PT,^5,12^ but most likely are associated with the densely packed structure and the complicated network of HBs involving fluorine atoms.

**Figure 1:**
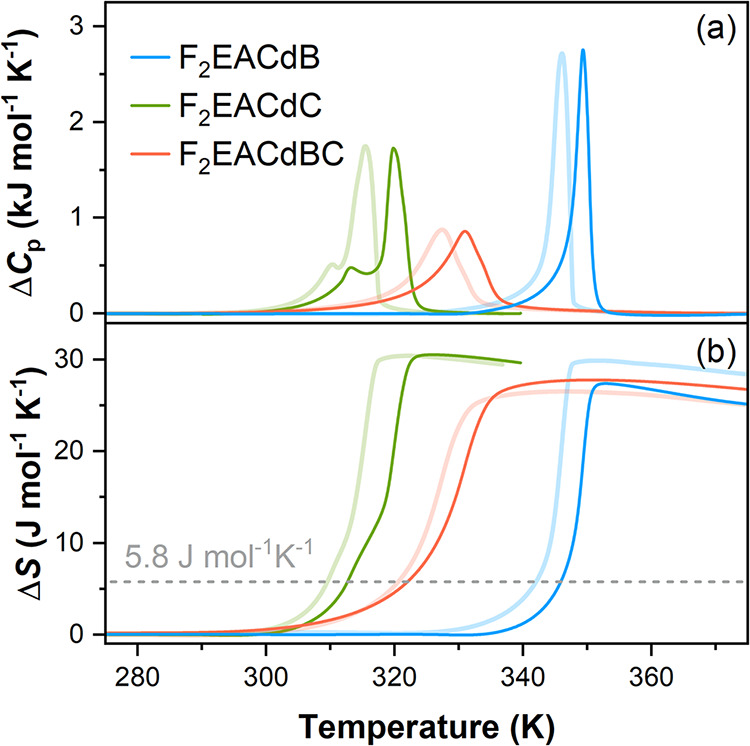
Estimated changes in heat capacity Δ*C*_p_ (a) and entropy Δ*S* (b) corresponding to PTs observed for **F**_**2**_ **EACdC**, **F**_**2**_ **EACdB**, and **F**_**2**_ **EACdBC**; the darker curves were calculated for heating runs, and the semitransparent ones for cooling runs; the horizontal gray dashed line at the Δ*S* value of 5.8 Jmol^–1^ K^–1^ corresponds to the ordering process from two disordered states (*N* = 2).

### Single-Crystal X-ray Diffraction

The crystal structure of **F**_**2**_ **EACdC** was previously reported by Song et al.;^12^ **F**_**2**_ **EACdBC** and **F**_**2**_ **EACdB** are isostructural analogues in both the LT and HT phases. The LT phase, measured at room temperature, is characterized by the orthorhombic space group *Pbc*a. The HT phase, determined at 350 K for **F**_**2**_ **EACdB** and at 340 K for the two other compounds, adopts the orthorhombic space group *Cmce*. Details of the diffraction experiments, including refinement parameters, are summarized in Table S1.

The asymmetric unit of all three compounds comprises one F_2_EA^+^ cation and an inorganic fragment containing one cadmium and two bromide/chloride ions (Figure S4). As in its FEA^+^ analogue,^5^ **F**_**2**_ **EACdBC** shows an almost statistical distribution of bromide and chloride ions at the equatorial positions. However, the axial sites are preferentially occupied by bromide (ca. 70:30), although to a lesser extent than in the previously described compound. The halide disorder in **F**_**2**_ **EACdBC** induces the disorder of the F_2_EA^+^ cation in the LT phase, where each of the two F atoms is disordered over two positions with a refined occupancy ratio of 62:38. In the LT phase, **F**_**2**_ **EACdC** and **F**_**2**_ **EACdB** are fully ordered. The comparison of the observed electron density of the F_2_EA^+^ cation in the three compounds is shown in Figure S5. In the HT phase, however, the F_2_EA^+^ cation is disordered over a mirror plane in all of the investigated compounds.

For consistency with the standard space group settings reported in^12^, the correspondence between the crystal axes of the HT and LT phases is maintained as *a*_HT_ → *b*_LT_, *b*_HT_ → *c*_LT_, and *c*_HT_ → *a*_LT_. Substitution of chloride with bromide ions (partial or complete) expands the two shorter lattice parameters but reduces the longest parameter (*b*_HT_) aligned between planes of corner-sharing inorganic octahedra (Table S1, Figures 2, S6, and S7). As expected, this reduction is greater in the HT phase for **F**_**2**_ **EACdB** than in **F**_**2**_ **EACdBC**, whereas in the LT phase, the trend is reversed, likely due to disorder of the F_2_EA^+^ in **F**_**2**_ **EACdBC** at this phase, which enhances F···F interactions (Table S2). Notably, partial replacement of chloride by bromide decreases the thermal expansion of the longest lattice parameter from +0.379 Å (+1.6% of *c*_LT_) in **F**_**2**_ **EACdC** to +0.242 Å (+1.1%) in **F**_**2**_ **EACdBC**, whereas full substitution induces negative thermal expansion (NTE) along the same direction, reaching −0.047 Å (−0.2%).

**Figure 2:**
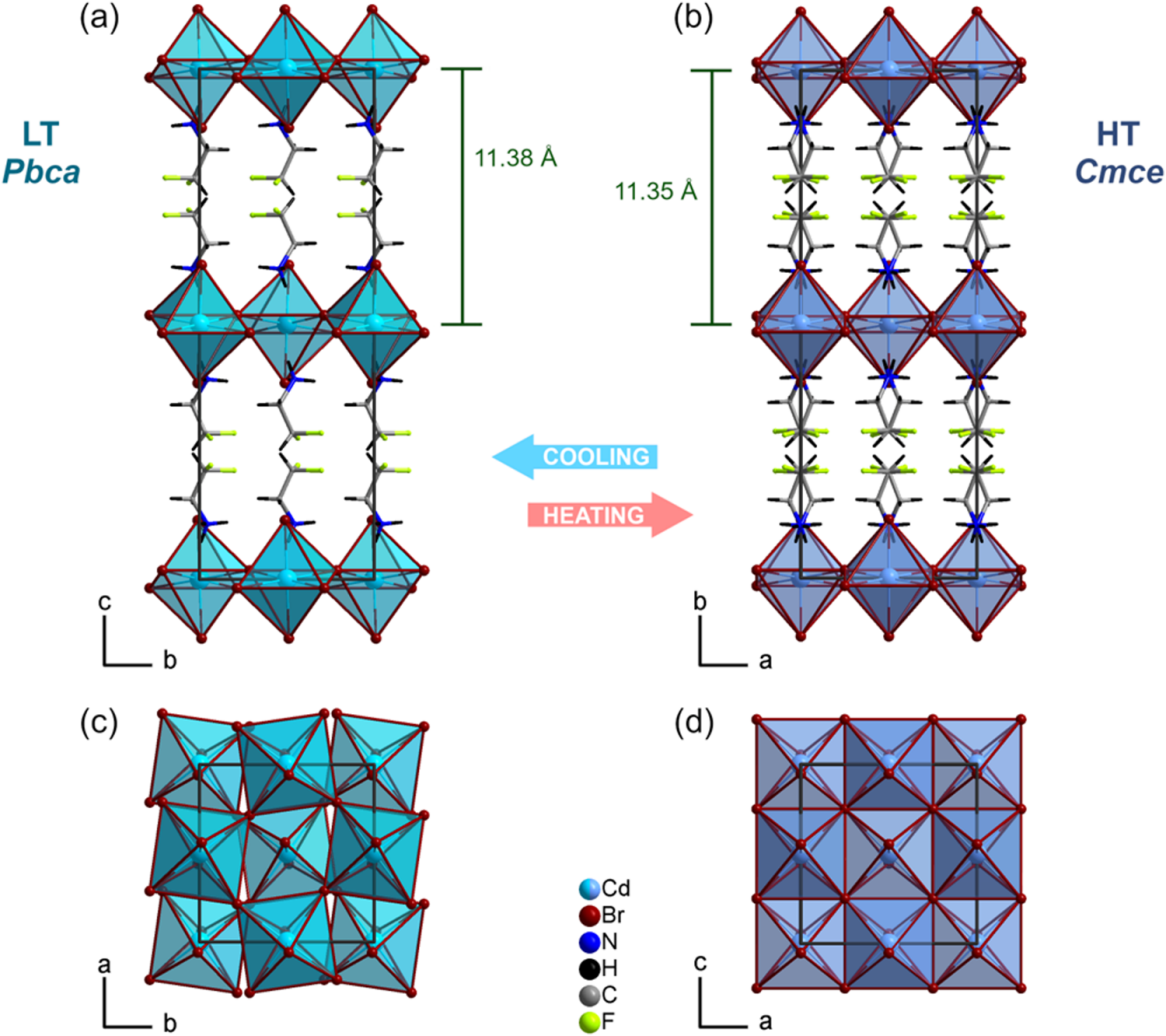
Selected corresponding views of the **F**_**2**_ **EACdB** structure in the LT phase (a, c) and the HT phase (b, d). In views (c) and (d), the organic part is omitted for clarity.

The structure arrangement is comparable to the FEA^+^ analogues,^5,12^ with corner-sharing octahedra arranged in layers and linked by a hydrogen-bonding network mediated by the cations (Figure S8). Consistent with this modification, the separation between adjacent inorganic layers is greater than in the FEA^+^ analogues. All HBs elongate progressively upon bromide substitution (**F**_**2**_ **EACdC** → **F**_**2**_ **EACdBC** → **F**_**2**_ **EACdB**; Table S3), except for the C2–H2···F1A^v^ contact observed in the LT phase of **F**_**2**_ **EACdBC** with substitutional disorder. This interaction arises from the positional disorder of fluorine atoms and therefore is not present in the other compounds.

An analysis of the octahedral parameters (Table S4) suggests that the larger polyhedral volumes observed in the bromine-substituted compounds primarily arise from differences in ionic radius. But in all compounds, polyhedral volumes decrease after PT to the HT phase. This is a result of a decrease in most of the Cd–Br/Cl distances with PT upon heating (Tables S5–S7). The most pronounced change is observed in **F**_**2**_ **EACdB**, where the decrease in equatorial Cd1–Br2 distances reaches up to 1% of *c*_LT_ (nearly 0.03 Å, Table S7). The decrease in both other compounds is similar, not greater than 0.7% of *c*_LT_ (less than 0.02 Å, Tables S7 and S8). Simultaneously, the slightly uneven change in equatorial and axial distances along with LT → HT PT causes an increase in the bond length distortion index (*Δ*). At the same time, bond angle variance (σ^2^) decreases along with LT → HT PT as the halide ions from octahedra are located on higher symmetrical sites, on mirror and glide planes (Figure 3).

**Figure 3:**
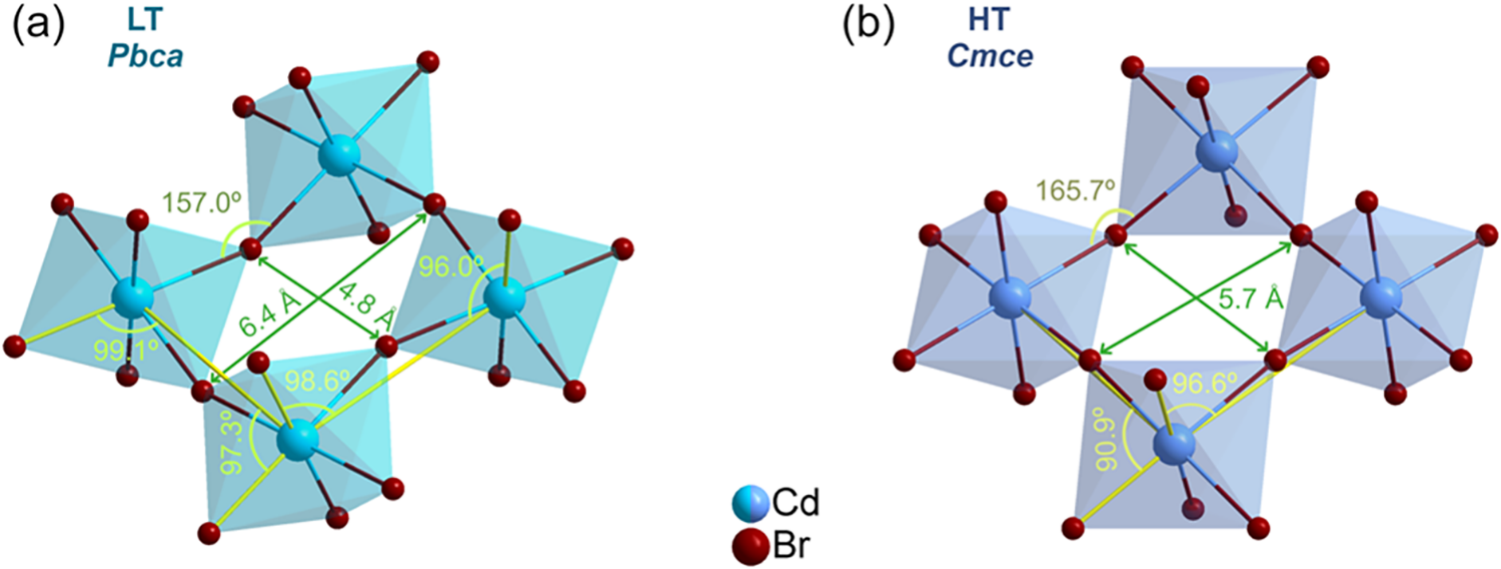
Comparison of octahedral tilting angles and interoctahedral distances in the inorganic layer of **F**_**2**_ **EACdB** in the LT phase (a) and the HT phase (b).

Intermolecular interactions were examined by constructing Hirshfeld surfaces for the F_2_EA^+^ fragment. The most significant interactions are highlighted in darker red on the Hirshfeld surface plots. Figure S9 presents corresponding plots for **F**_**2**_ **EACdB**. Notably, the contribution of F···F contacts to the Hirshfeld surface increases with LT → HT PT. Two-dimensional fingerprint plots were generated to visualize intermolecular contact distances, with the highlighted regions corresponding to F···F, H···F/F···H, and H···Br/Cl interactions (Figure S10). The quantitative data for all three compounds are summarized in Table S8. The contribution of F···F contacts increases from 8% in the LT phase to approximately 25–26% in the HT phase for **F**_**2**_ **EACdC** and **F**_**2**_ **EACdB**. In **F**_**2**_ **EACdBC**, however, the positional disorder of fluorine atoms in the LT phase leads to a higher F···F contribution (ca. 15%), which becomes comparable to two other compounds in the HT phase (ca. 26%). In the LT phase, H···F/F···H contacts contribute to the Hirshfeld surface to a similar extent as H···Br/Cl, both accounting for approximately 40% in all compounds. In the HT phase, the contributions of H···Br/Cl remain nearly unchanged (varying by +0.7% to −2.0%), whereas the H···F/F···H contributions decrease to roughly one-third of their initial LT-phase values.

As anticipated, the volume of void domains, corresponding to the unoccupied regions within the crystal lattice, decreases across the LT → HT phase transition. In the HT phase, it also decreases upon bromide substitution, reaching 38.19 Å^3^ in **F**_**2**_ **EACdB**, which corresponds to 2.71% of the unit cell volume (Table S9, Figure S11). Such densely packed structures are therefore expected to exhibit short intermolecular contacts. Consistent with this, the studied crystals display some of the shortest reported intermolecular F···F distances in the HT phase, measuring 2.52 Å, 2.47 Å, and 2.45 Å, for **F**_**2**_ **EACdC**, **F**_**2**_ **EACdB**, and **F**_**2**_ **EACdBC**, respectively.^23^ Selected F···F distances are listed in Table S2 and for **F**_**2**_ **EACdB** shown in Figure S12. In the Cambridge Structural Database (CSD),^46^ only 6 structures were found involving distances less than twice the van der Waals radius of fluorine (<2.94 Å) with monofluorinated compounds (Figure S13). The shortest distance was found to be 2.69 Å (Refcode: AVAHOW).^47^ The number of difluorinated compounds in the same search was eight times greater (48 structures), with the shortest distance of 2.57 Å (Refcode: FUDGIY).^48^ For the trifluorinated structures, there are evidently more results (1586 structures) with 4 structures with F···F distances less than 2.45 Å, with the shortest found distance equal to 2.32 Å (Refcode: YIZKEY).^49^ Based on these facts, the crystals studied here may be new examples of rare compounds with homohalogen F···F bonds; however, this should be further confirmed by calculations. In contrast to F_2_EA^+^-based cadmium halides, in previously reported monofluorinated FEA_2_CdX_4_, the intermolecular F···F distances are longer (3.16 Å), excluding the presence of halogen bonds.^5^

Due to the presence of highly electronegative F atoms in the cation, a relatively high dipole moment is sensitive to small changes in geometry. Fluorine atoms very rarely create typical halogen bonds like Cl, Br, and I;^24,50^ thus, the reported short intermolecular F···F contacts have been mostly explained because of the dense packing rather than real stabilizing interactions.^22,23^

### NCI Calculations

To investigate the nature of the short F···F interactions between cations, NCI calculations were performed for a single F_2_EA^+^ cation and its dimer, based on the geometry in both the LT and HT phases of **F**_**2**_ **EACdB**. Figures S14 and S15 present the results as RDG plots as a function of sign­(λ_2_)­ρ. The value of sign­(λ_2_)­ρ characterizes the strength and nature of interactions as follows: attractive forces (λ_2_ < 0, blue), van der Waals forces (λ_2_ ≈ 0, green), and repulsive attractions (λ_2_ > 0, red).^51^

In HT, a single cation exhibits a distribution of interactions (Figure S14a) with a dominant contribution of repulsive forces between neighboring fluorine atoms, visible as a red area (Figure S14b). A comparison with the RDG plot for the dimer does not reveal significant changes in the red and blue regions (Figure S14c), indicating the absence of additional strong interactions. Differences are observed only in the green region, corresponding to the emergence of weak intermolecular van der Waals F···F contacts (Figure S14d) and C–F···H hydrogen bonds.

The PT to LT leads to a slight decrease in repulsion between neighboring fluorine atoms (Figure S15), accompanied by an increase in weak interactions, mainly HBs. In the dimeric configuration, these weak interactions are pronounced. Similar to HT, no significant changes in attractive interactions are observed for a dimer.

Overall, the NCI analysis demonstrates that the shortest F···F contacts in **F**_**2**_ **EACdB** are predominantly repulsive, suggesting that the observed crystal rigidity arises from dense packing rather than stabilizing attractive interactions.

### Phonon Properties

#### DFT Calculations and Band Assignment

The geometry of the optimized F_2_EA molecule and its protonated analogue are presented in Table S10. Figure S16 presents the numbering used for calculations, and Figure 4 demonstrates the room-temperature IR and Raman spectra compared to the calculated spectra of F_2_EA^+^ in harmonic or anharmonic approximation. Theoretical spectra show very good agreement with experimental ones, except for bands originating from groups that participate in hydrogen bonding. The IR and Raman spectra calculated for a neutral molecule are presented in Figure S17.

**Figure 4:**
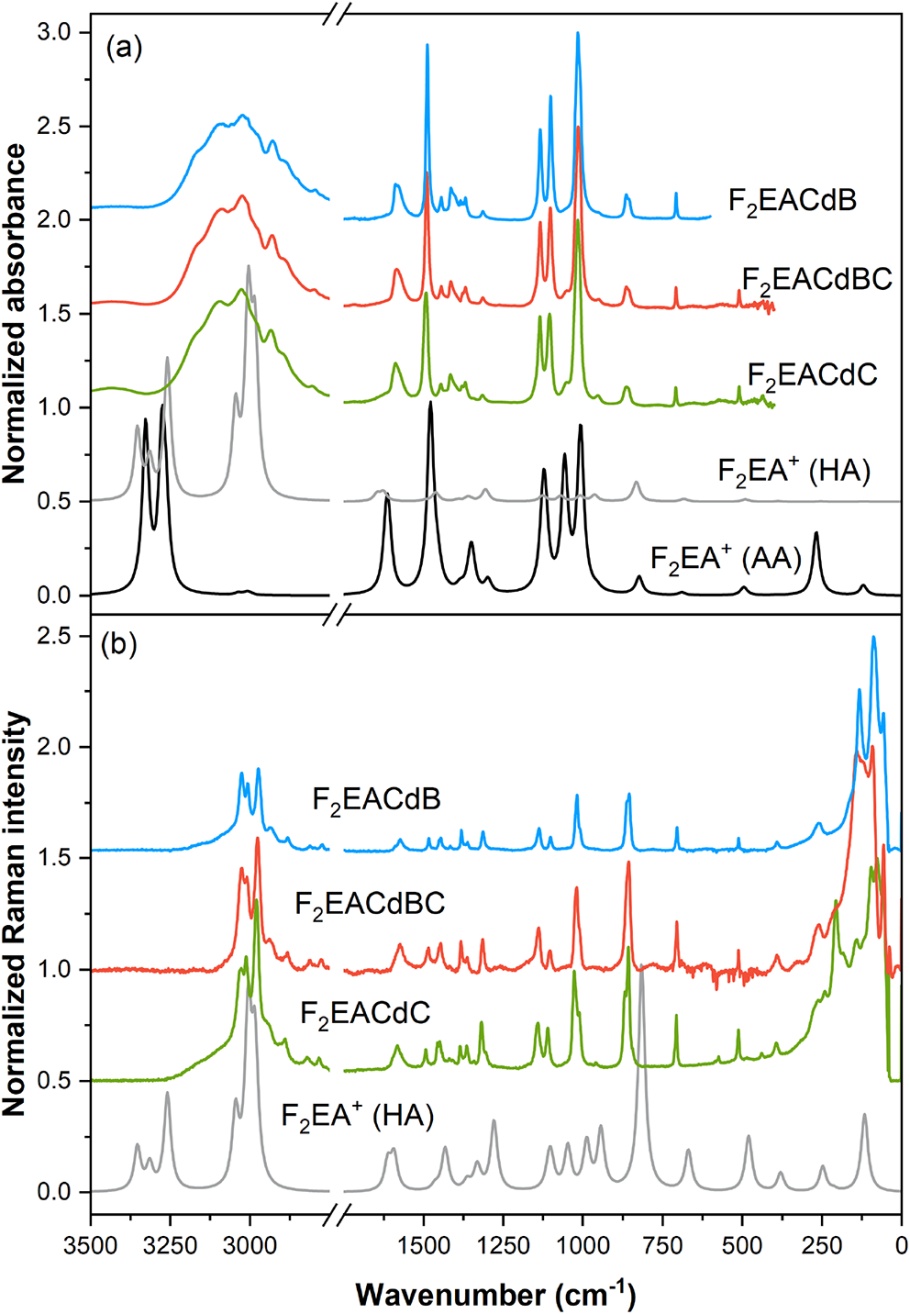
Comparison of room-temperature experimental IR (a) and Raman (b) spectra of **F**_**2**_ **EACdC**, **F**_**2**_ **EACdB**, and **F**_**2**_ **EACdBC**, together with calculated IR and Raman spectra of F_2_EA^+^ in harmonic (HA) and anharmonic approximation (AA); the HH spectra were scaled by 0.98 (2499–0 cm^–1^) and 0.96 (3600–2500 cm^–1^).

Table S11 contains all calculated wavenumbers and values of PEDs for each mode. They were further used to propose the assignment of bands listed in Table S12. The DFT results are in good agreement with the internal vibration assignments proposed in the literature for compounds with EA^+^ or its derivatives^52–54^ and with previous *ab initio* calculations for F_2_EA performed by Durig et al.^55^

Regarding the internal vibrations of CdX_6_ octahedra, their assignments are easy, as the νCdCl (stretching) modes observed at 207, 184, and 142 cm^–1^ are downshifted for νCdBr to 164 and 134 cm^–1^ (Table S12).

#### Temperature-Dependent IR Spectra

The comparison of normalized mid-IR spectra from the 7 (or 20 K for bromide), depending on the sample, to 400 K is demonstrated in Figure S18. Figure S19 compares the spectra at the lowest temperature, room temperature, and 400 K for a better visualization. At 7 K (20 K for **F**_**2**_ **EACdB**), the spectra exhibit narrow bands that gradually broaden to the PT temperatures. The transition to the high-temperature phase is clearly visible as a very strong broadening and overlaying of the bands, as well as shifts (see factor group analysis in the SI). It is important to note that the narrowest bands at cryogenic temperatures are observed for the **F**_**2**_ **EACdB** network, in which organic cations have the highest molecular space of 174.9 Å^3^, defined as the unit cell volume divided by the number of cations in the cell. For **F**_**2**_ **EACdC**, bands are broader because the volume per cation is significantly decreased to 160.2 Å^3^, cations are more strongly confined and coupled to the inorganic layers. For the mixed crystals, this value is 168.8 Å^3^, but the observed broadening is also contributed by a substitutional disorder.

In addition, PT causes a strong increase in the intensity of bands at approximately 1575, 1425, 1120, 1050, and 950 cm^–1^ (see arrows for the most intense bands in Figure S19). In the LT phase, they are weakly temperature-dependent. This interesting behavior is discussed further.

#### NH_3_^+^ Groups and Manifestations of the N–H···X Hydrogen Bonds

According to our DFT calculations, the wavenumbers of the ν_as_NH_2_ and ν_s_NH_2_ stretching modes in anharmonic approximation for the F_2_EA molecule are 3397 and 3351 cm^–1^, respectively (Table S11). The protonation of the amino groups leads to the downshift by 88–121 cm^–1^. The interaction with the nonorganic subnetwork causes a further strong decrease, the magnitude of which corresponds directly to the strength of the N–H···X contacts. In the F_2_EA^+^-based crystals, due to complex contours above 3000 cm^–1^, it is difficult to estimate the HBs strength based on the IR spectra. Despite the significant influence of stronger N–H···X HBs on the νNH_3_^+^ bands in **F**_**2**_ **EACdC**, as resulted from sc-XRD (Table S3), in both the LT and HT phases, there are no clear red shifts, reflecting the HB strength.

The thermal behavior of bands with the contribution of the amino group vibrations is typical; softening of modes is observed with increasing temperature (Figures 5, S20, and S21). At the PT temperature, all components of broad and complex contours associated with δ_as_NH_3_^+^ and δ_s_NH_3_^+^ merge into a single and broadband and expand significantly (Figures 5, S18, S19, S22, and S23), which is an indicator of the first type of order–disorder transformation connected with the breaking of HBs and unlocking the F_2_EA^+^ movements.

**Figure 5:**
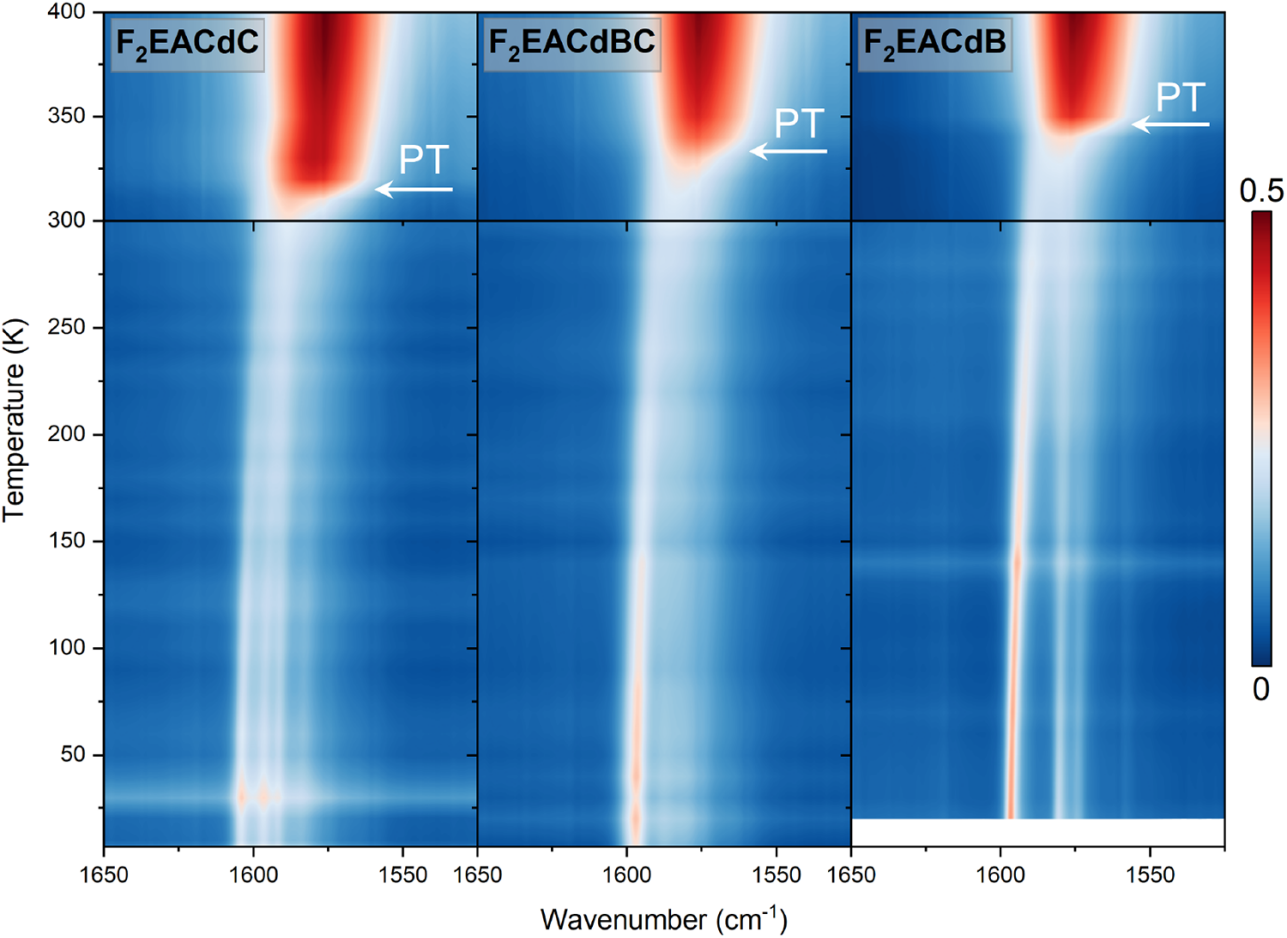
Comparison of thermal evolution intensity maps for bands corresponding to δ_as_NH_3_^+^.

Another interesting feature is a weak splitting for the intense bands associated with the δ_s_NH_3_^+^ molecular vibrations at cryogenic temperatures (see Figures S18c, S19d, and S23). For **F**_**2**_ **EACdC**, there is a 1496 and 1489 cm^–1^ doublet, which, for **F**_**2**_ **EACdBC**, appears as a band at 1488 cm^–1^ with an asymmetry (shoulder) at 1492 cm^–1^. For **F**_**2**_ **EACdB**, only one narrow band at 1488 cm^–1^ is observed. This behavior can be explained by Davydov splitting associated with the presence of eight equivalent F_2_EA^+^ cations in the primitive cell. The splitting is largest for the chloride crystal (7.1 cm^–1^), smaller for the mixed compound (3.2 cm^–1^), and negligible for the bromide analogue (below the spectral resolution of 2 cm^–1^). Stronger splitting for chloride implies that cations experience stronger coupling and dipole–dipole interactions, likely due to the reduced interlayer spacing in the LT phase.

#### Vibrational Modes of C–H Bonds in Methylene and Difluoromethyl Groups

Figures S16, S17, and S19 demonstrate that the thermal response of bands involving vibrations of C–H bonds is weaker in comparison to those involving N–H modes. This is because, in most cases, they are strongly coupled with other vibrations, as indicated by our DFT calculations (Table S11). Furthermore, the C–H···X HB interactions are relatively weak, making them less sensitive to temperature changes. The exception is stronger C–H···F-type HBs, but these are broken in the HT phase and affect cations in the ordered LT phase.

#### IR Characteristics of the CCN Skeleton

According to our DFT calculations, the νCC and νCN vibrations are coupled with the νCF and δCH_2_ vibrational modes. Their temperature response deviates from a typical anharmonic dependence, but due to their low intensity, the fitting errors are bigger. The most important feature is that bands near 950 cm^–1^ with the strongest contribution of νCN do not exhibit any pronounced changes at the PT temperature, suggesting that the geometry of the skeleton is only negligibly affected by transformation. This behavior is observed regardless of the type of ligand. The intensity evolution maps for bands between 850 and 900 cm^–1^, with strong contribution of νCC and νCN, are presented in Figure S26.

#### Vibrational Modes Involving F Atoms

As mentioned above, Figures 6, S18, and S19 show three bands with unusually increasing intensity with higher temperature. Since the effect is most pronounced for bands with a strong contribution from vibrating F atoms, we propose that it provides additional evidence for strong F···F interactions affecting the oscillating dipoles. A strong temperature dependence in HT, and negligible in the LT phase, is interpreted as a redistribution of oscillator strength driven by dynamic orientational disorder, rather than a population crossover between two states in the HT phase. In the LT phase, where F_2_EA^+^ cations are immobilized, these bands remain weak. Their increase in intensity upon heating is evidence of enhanced molecular dynamics in the HT phase up to 400 K. A similar increase in intensity accompanied by red shift has previously been observed for νCI, providing a distinct signature of the halogen bonding in iodopentafluorobenzene.^56^

**Figure 6:**
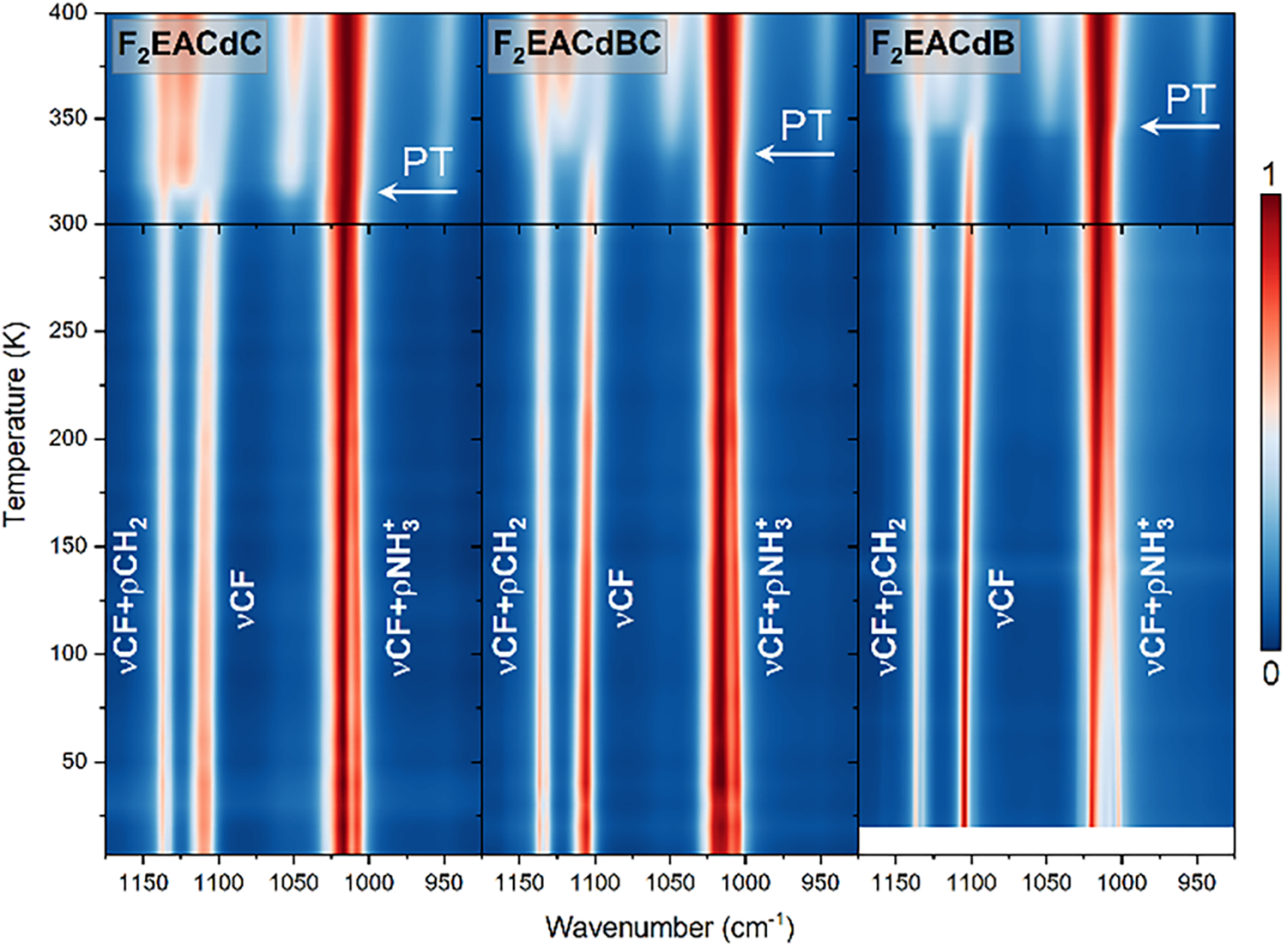
Comparison of thermal evolution intensity maps for bands having a contribution of νCF.

The dependence of the bandwidth for νCF modes is unusual (Figure S22c), as it shows a continuous, strong, and rapid increase in the HT phase, amounting to 0.29, 0.24, and 0.25 cm^–1^ K^–1^ for **F**_**2**_ **EACdC**, **F**_**2**_ **EACdBC**, and **F**_**2**_ **EACdB**, respectively. This behavior most likely arises from the strong anharmonicity of these vibrations, enhanced phonon–phonon interactions, and coupling between molecular vibrations with the inorganic sublattice. This increase is several times stronger than for compounds with a monofluorinated derivative (0.04 cm^–1^ K^–1^ for FEA_2_CdCl_4_ and 0.05 cm^–1^ K^–1^ for FEA_2_Cd­[Br_0.66_Cl_0.34_]_4_),^5^ which can be attributed to stronger fluctuations in the dipole moment. For this reason, the jump during PT is less pronounced for the νCF modes, as they are governed by local fluctuations rather than long-range order. This interpretation is further supported by the much weaker thermal response for γCF vibrations, which are considerably less sensitive to local fluctuations (Figure S22d).

It is also worth noting that the bandwidth of the νCF mode in the LT phase is significantly higher for the **F**_**2**_ **EACdBC** compound (Figure S22c). This observation correlates well with the sc-XRD results, demonstrating statistical disorder of the F atoms in this phase for the mixed compound.

### Mechanism of Phase Transition

Certainly, the PT mechanism in (F_2_EA)_2_CdX_4_ is not dominated by a sudden ordering, as indicated by DSC studies. The pure order–disorder contribution to Δ*S* is estimated to be 20%; therefore, the remaining approximately 80% arises from displacive effects, including octahedral tilting, twisting, and distortion, as well as changes within the complex network of diverse HBs and forces resulting from the presence of F atoms. Based on the literature data, the contribution of the inorganic sublattice is not strong, as there is no clear correlation between its deformation and high entropy change.^7–9^

The cooperative F···F contacts affect the arrangement of the crystal structure and enforce the structural order, requiring much more thermal energy to induce PT in the relatively rigid LT phase. The F_2_EA^+^ cations have limited librational and translational freedom, which accounts for the large change in entropy observed during PT to a disordered HT phase, where this confinement is reduced. Furthermore, a higher negative potential of the cation close to the F atoms leads to the formation of relatively strong N–H···X HBs, along with additional weak C–F···H and C–F···X contacts, increasing the required energy to activate the 2-fold disorder.

The fluorination increases the rotational barrier of the CF_2_H group and enhances coupling between cations and the inorganic sublattice, further contributing to the rigidity of the LT phase.

PT is also accompanied by a minor deformation of the inorganic sublattice, indicating weak octahedral tilting and twisting within the plane of the inorganic layers. Although ligand substitution significantly alters the volume available for the cations in both phases, thereby modifying the interaction strengths and the PT temperature, it does not substantially change the overall transition mechanism.

### Optical Properties

The diffuse reflectance spectra of the **F**_**2**_ **EACdB**, **F**_**2**_ **EACdBC**, and **F**_**2**_ **EACdC** crystals presented in Figure S25a clearly show that the investigated materials exhibit strong absorption in the UV range. The absorption edge for chloride is observed at 230 nm and red shifts as the Br^–^ concentration increases, i.e., to 274 nm for **F**_**2**_ **EACdBC** and to 282 nm for **F**_**2**_ **EACdB**. Moreover, the diffuse reflectance spectra contain other transitions whose absorption maximum red shifts from 286 to 324 nm as the bromine concentration in the sample increases. According to the literature reports, it appears that these bands also belong to the host lattice, as they are observed in other hybrid perovskites with a similar structure.^5,57^

On the basis of the above measurements, the sizes of the energy band gaps (*E*_g_) in the studied materials were determined using the Kubelka–Munk theory^58^ and are depicted in Figure S25b–d. As expected, the *E*_g_ decreased from 5.24 eV for the chloride analogue through 4.36 eV for the sample containing both halides to 4.24 eV for the chloride-free crystals. The order of magnitude of the obtained *E*_g_ values is quite close to the literature data for organic–inorganic cadmium-containing hybrid halides.^5,11,59–62^

Figure 7a,7c reveals that excitation of the investigated materials with a laser diode operating in the UV range (266 nm) results in the generation of broad emission consisting of two components. The first one is characterized by a significantly narrower fwhm and an emission maximum at 362 nm for **F**_**2**_ **EACdB** and 372 nm for **F**_**2**_ **EACdBC**, whereas the second one covers a wider spectral range, with its maximum located at 615 nm for **F**_**2**_ **EACdB** and 530 nm for **F**_**2**_ **EACdBC**. Considering the fwhm and the position of both components, it can be concluded that their origins are distinct. The large Stokes shift and relatively long lifetime (microseconds) of the bands located in the UV range exclude their association with a free exciton (FE), which is characterized by a small distance between its emission and absorption maxima as well as a lifetime of the order of nanoseconds. It therefore seems likely that these bands are related to structural defects, the presence of which has already been described in 2D organic–inorganic halides.^63^ On the other hand, the second transition located in the visible range may be associated with a self-trapped exciton (STE) due to strong electron–phonon coupling induced by lattice distortions caused by structural modifications.^57,64,65^ The scattering of the excitation beam on the sample is the reason for the asymmetric shape of the emission spectrum of the **F**_**2**_ **EACdB** crystals.

**Figure 7:**
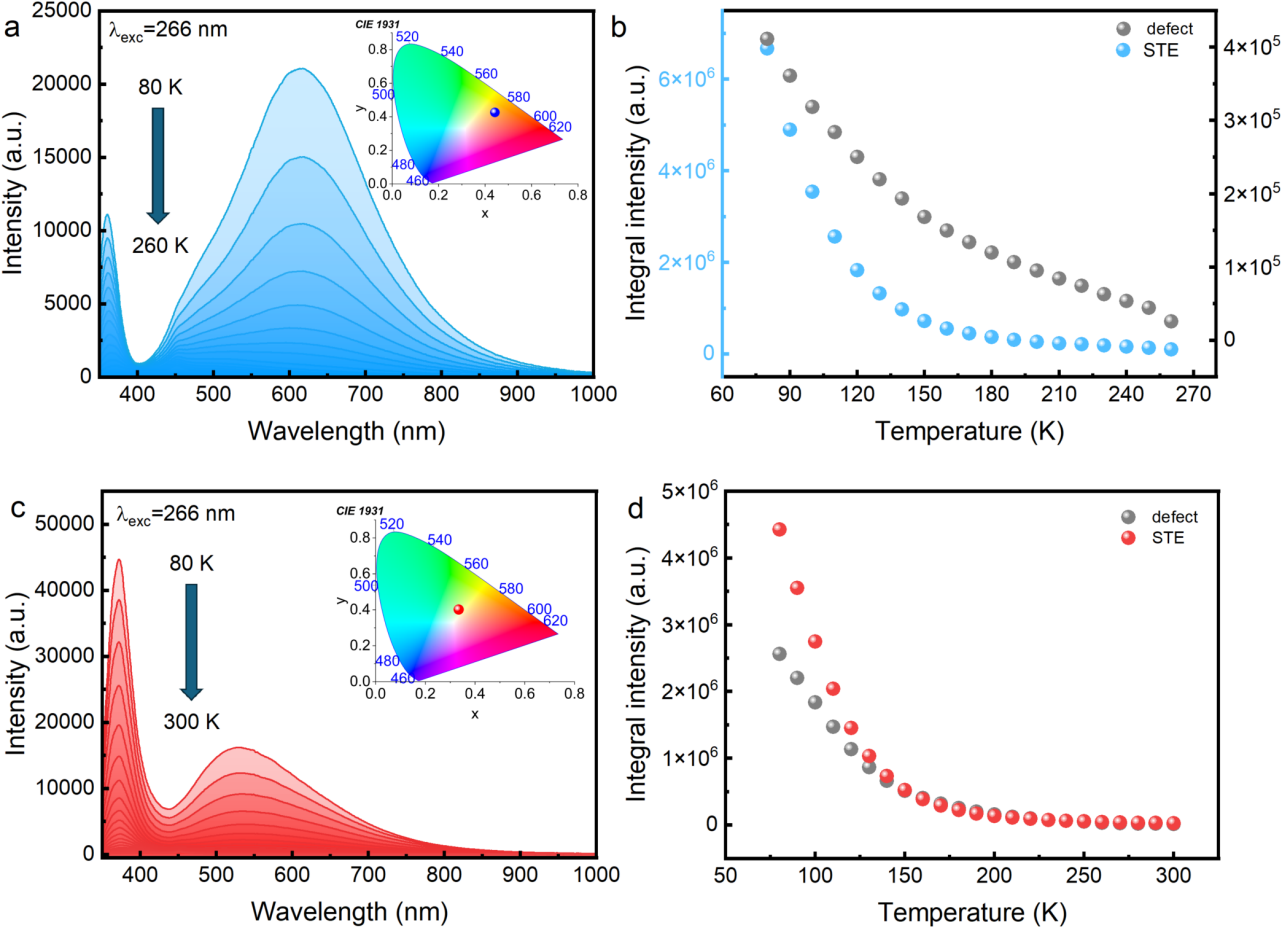
Emission spectra for **F**_**2**_ **EACdB** (a) and **F**_**2**_ **EACdBC** (c) crystals measured as a function of temperature, together with their integral intensities (b, d) and CIE chromaticity diagrams determined at 80 K.

It is worth noting that spectroscopic studies of a similar material, but without fluorine, have already been reported.^11^ Interestingly, the authors of this report recorded only a narrow emission peak at 388 nm, while the broadband STE emission was not observed. Therefore, it can be assumed that the structural changes caused by the presence of fluorine in the **F**_**2**_ **EACdB** and **F**_**2**_ **EACdBC** crystal structures suggest stronger electron–phonon coupling and, consequently, the generation of STE emission.

The majority of reports on 2D hybrids have demonstrated that the optoelectronic properties in hybrid halides are strongly governed by ligand and organic cation substitutions, both of which are responsible for the lattice dynamics.^66,67^

Analysis of the structural data indicates that the observed dual emissions cannot be directly attributed to distortion of the CdX_6_ octahedra (see Table S4), as both monitored parameters, the distortion index (*Δ*) and bond angle variance (σ^2^), are comparable for all three analogues in the LT phase (0.0222–0.0252, and 1.591–1.665, respectively). For nonfluorinated chloride and bromide analogues, they are slightly different, amounting to 0.0169 and 0.0304 (*Δ*), as well as 5.81 and 5.71 (σ^2^), respectively.^11,68^ Another important structural feature affecting the PL response is the Cd–X–Cd angle, reflecting the tilting of octahedral units.^69,70^ For EA_2_CdCl_4_ and EA_2_CdBr_4_, they are equal to 162.0 and 159.5°.^11,68^ For difluorinated compounds, the tilting also increases with bromide ligand concentration, i.e., 158.6, 158.4, and 157.0° (Tables S5–S7). A comparison of the structures also shows the increase in the interlayer distances for the F_2_EA^+^-based compounds (11.35–11.52 Å) and nonfluorinated analogues (11.05–11.00 Å). The increase in Br^–^ concentration is also followed by a decrease in the N–H···X interactions, namely, from 3.225 to 3.337 Å for **F**_**2**_ **EACdC**, 3.339–3.455 Å for **F**_**2**_ **EACdCB**, and 3.386–3.494 Å for **F**_**2**_ **EACdB** (Tables S3), as well as 3.251–3.381 Å for EA_2_CdCl_4_, and 3.489–3.593 Å for EA_2_CdBr_4_.^11,68^

Although the octahedral distortion and tilting vary slightly for fluorinated analogues, the PL response substantially differs, suggesting that these structural parameters do not primarily govern the emission. The observed behavior can be explained by the softer and more polarizable crystal structure of **F**_**2**_ **EACdB**. In contrast, stronger N–H···X HBs in **F**_**2**_ **EACdBC** make the lattice more rigid, affecting its optical properties. This effect is further enhanced by fluorination (Tables S2 and S8), which increases structural rigidity and intermolecular interactions, influencing the PL response of the material.

Considering the above, the composition of the material affects spectroscopic properties such as the shape of the emission spectrum, the position of its maximum, and changes in the luminescence intensity ratio of individual components. Nevertheless, CIE diagrams show that at 80 K, the **F**_**2**_ **EACdB** and **F**_**2**_ **EACdBC** crystals exhibit similar emission colors, but the material containing mixed halides displays a color much closer to ideal white light because the narrower component shifts toward the visible range. Figure 7b,7d proves that the highest emission intensity for both luminescence centers was obtained at 80 K. An increase in temperature led to a gradual quenching of emissions attributed to increasing lattice vibrations and the occurrence of new nonradiative relaxation channels.^71^ As a result, the luminescence of both samples is quenched before reaching room temperature. The nearly identical trends in the integral emission intensity of STE luminescence for both samples indicate that these transitions are of a similar nature. On the other hand, the higher number of defects in **F**_**2**_ **EACdBC** crystals, due to the presence of two halide ions compared to a single halide ion in **F**_**2**_ **EACdB** crystals, results in a different temperature evolution of defect-related emission. It is worth mentioning that because the absorption bands of **F**_**2**_ **EACdC** crystals are in the deeper ultraviolet range (Figure S25), it was not possible to excite the material, and therefore, its emission was not recorded. Applying the modified Arrhenius equation^72,73^ allowed us to calculate the activation energy of thermal quenching (*E*_a_) for both structural defect-related and STE emission bands for both glowing samples (Figure S26). It turned out that *E*_a_ for both luminescence centers for **F**_**2**_ **EACdB** and **F**_**2**_ **EACdBC** crystals is in the range of 44–67 meV. The obtained values are approximately half those determined for FEA_2_Cd­[Br_0.66_Cl_0.34_]_4_ crystals with a very similar structure but with a monofluorinated cation.^5^ This highlights the impact of fluorination on optical properties.

The lifetimes of emissions from **F**_**2**_ **EACdB** and **F**_**2**_ **EACdBC** crystals were determined based on low-temperature measurements of luminescence decay curves under 266 nm pulse excitation and monitoring of structural defect-related and STE emission maxima. The recorded luminescence profiles are nonexponential in both cases, as can be seen in Figure S27. This means that, in addition to radiative energy transfer, nonradiative energy transfer also occurs in the samples. After fitting the curves with a double-exponential function, it was found that both defect-related and STE emissions have an average lifetime of 13 and 0.85 μs, respectively, for **F**_**2**_ **EACdB** crystals, and 35 and 5.74 μs, respectively, for **F**_**2**_ **EACdBC** crystals. Such long lifetimes are not common for STE emission in hybrid halides but are still reported in the literature.^74–76^ It is assumed that ligand substitutional disorder and the number of defects affect the depth of the electron-trapping wells and, consequently, the luminescence lifetimes of both defect-related and STE emissions.

## Conclusions

The crystals of 2D hybrids – cadmium bromide, chloride, and chlorobromide (∼60 mol %) containing a 2,2-difluoroethylammonium cation – were grown using a simple wet chemistry method.

The thermodynamic properties of crystals were investigated using the DSC technique and revealed the presence of complex order–disorder phase transitions and lattice distortions associated with a significant change in entropy. Partial halide substitution introduces higher structural disorder and raises the phase-transition temperatures as the Br content increases.

The single-crystal XRD analysis demonstrated that the mixed-halide crystal exhibits an emergence of the ligand and F atoms disorder, as opposed to chloride and bromide, which are fully ordered in the LT phase. The strength of conventional N–H···X and weaker C–F···X, but equally important, hydrogen bonds was considered in terms of the occurrence of dynamical disorder in the HT phases and co-occurring changes in the slabs of octahedral units. Increasing halide size improves intermolecular interactions, especially the F···F connections. The presented difluorocompounds exhibit exceptionally short F···F intermolecular distances; however, the noncovalent interaction calculations confirmed their repulsive nature.

It was demonstrated that vibrational spectroscopy combined with DFT quantum calculations for a single molecule and its protonated cation is a powerful tool for studying the dynamics of difluorinated cadmium hybrids and the properties and influence of F atoms on the crystal properties. Temperature-dependent IR studies showed that the mechanism of phase transitions in all three hybrids is primarily governed by breaking cooperative hydrogen bonding, which allows for cation hopping between two equivalent positions. This process reduces octahedral tilting without causing significant octahedral deformation.

The effect of ligand substitution in the studied hybrids alters the strength of interplaying interactions rather than the mechanism. The optical and vibrational properties further confirmed that different degrees of selective fluorination and halide mixing provide an effective way to finely control the lattice dynamics and emission behavior in hybrid cadmium halides. Our studies show that octahedral distortion and tilting in the inorganic layers are not the key factors in inducing differences in luminescent behavior.

## Supplementary Material



## Data Availability

The raw experimental data are available at 10.5281/zenodo.18978495.

## Notes

EDS spectra, SEM pictures, powder XRD, DSC, asymmetric units, electron densities, crystal structures, Hirshfeld surfaces, fingerprint plots, visualization of void domains, C–H···F interactions, CSD search result for the F···F distances, atom numbering, RDG vs sign­(λ_2_)­ρ plots and RDG isosurfaces, calculated IR and Raman spectra, temperature-dependent IR spectra, changes in positions and width of IR bands as a function of temperature, thermal evolution of spectra presented as maps, diffuse reflectance spectra, activation energy calculation, luminescence decay profiles, crystallographic data, F···F distances, HB parameters, octahedral distortion parameters, bond distances, angles, intermolecular contacts, volumes of void domains, structural parameters of the optimized molecule and cation, calculated IR and Raman wavenumbers, and assignment of IR and Raman bands (PDF)

## References

[1] Saparov B., Mitzi D. B. (2016). Organic-Inorganic Perovskites: Structural Versatility
for Functional Materials Design. Chem. Rev..

[2] Park G., Oh I. H., Park J. M. S., Park S. H., Hong C. S., Lee K. S. (2018). Investigation
of Magnetic Phase Transition on the Layered
Inorganic-Organic Hybrid Perovskites (C_6_H_5_CH_2_CH_2_NH_3_)_2_MnCl_4_ by
Single-Crystal Neutron Diffraction. Phys. B.

[3] Jena A. K., Kulkarni A., Miyasaka T. (2019). Halide Perovskite
Photovoltaics:
Background, Status, and Future Prospects. Chem.
Rev..

[4] Mączka M., Zaręba J. K., Gągor A., Stefańska D., Ptak M., Roleder K., Kajewski D., Soszyński A., Fedoruk K., Sieradzki A. (2021). [Methylhydrazinium]_2_PbBr_4_, a Ferroelectric Hybrid Organic-Inorganic
Perovskite with
Multiple Nonlinear Optical Outputs. Chem. Mater..

[5] Ptak M., Kowalska D., Smółka S., Stefanski M., Osmólska J., Szymański D., Sieradzki A. (2025). Ligand Substitutional
Effects on Molecular Dynamics and Broadband Near-White Emission in
2D 2-Fluoroethylammonium Cadmium Chlorobromide. J. Phys. Chem. C.

[6] Akkerman Q. A., Manna L. (2020). What Defines a Halide
Perovskite?. ACS Energy
Lett..

[7] Wang C. F., Fan X. W., Tan Y. H., Wei W. J., Tang Y. Z. (2019). High-Temperature
Reversible Phase Transition and Switchable Dielectric and Semiconductor
Properties in a 2D Hybrid [(C_3_H_12_N_2_O)­CdCl_4_]_n_. Eur. J. Inorg.
Chem..

[8] Wang Z., Lu Y., Chen H. P., Ge J. Z. (2017). Controllable Structures Designed
with Multiple-Dielectric Responses in Hybrid Perovskite-Type Molecular
Crystals. Inorg. Chem..

[9] Sun X. F., Wang Z., Li P. F., Liao W. Q., Ye H. Y., Zhang Y. (2017). Tunable Dielectric
Responses Triggered by Dimensionality Modification
in Organic-Inorganic Hybrid Phase Transition Compounds (C_5_H_6_N)­Cd_n_Cl_2n+1_ (n = 1 and 2). Inorg. Chem..

[10] Yue Z. Y., Luo W., Wang N., Li H. K., Xu Z. J., Feng Y., Shi C., Ye H. Y., Miao L. P. (2023). Two-Dimensional Organic–Inorganic
Hybrid Perovskite Ferroelastics: (PEA)_2_[CdCl_4_], (3-FPEA)_2_[CdCl_4_], and (4-FPEA)_2_[CdCl_4_]. CrystEngComm.

[11] Kucheriv O.
I., Sirenko V. Y., Shova S., Gural’skiy I.
A. (2024). 2D Hybrid
Organic-Inorganic Perovskite Displaying Narrow-Band Violet-Blue Photoluminescence. J. Lumin..

[12] Song N., Chen S. P., Fan X. W., Tan Y. H., Wei W. J., Tang Y. Z. (2020). Regulating Reversible Phase Transition
Behaviors by
Poly-H/F Substitution in Hybrid Perovskite-Like 2­[CH_2_FCH_2_NH_3_]·[CdCl_4_]. ACS Omega.

[13] Chu L.-L., Zhang T., Gao Y.-F., Zhang W.-Y., Shi P.-P., Ye Q., Fu D.-W. (2020). Fluorine Substitution
in Ethylamine Triggers Second
Harmonic Generation in Noncentrosymmetric Crystalline [NH_3_CH_2_CH_2_F]_3_BiCl_6_. Chem. Mater..

[14] Sha T. T., Xiong Y. A., Pan Q., Chen X. G., Song X. J., Yao J., Miao S. R., Jing Z. Y., Feng Z. J., You Y. M., Xiong R. G. (2019). Fluorinated
2D Lead Iodide Perovskite Ferroelectrics. Adv.
Mater..

[15] Rao W., Li M., You X., Wei Z., Zhang M., Wang L., Cai H. (2021). The Role of Fluorine-Substituted Positions on the Phase Transition
in Organic-Inorganic Hybrid Perovskite Compounds. Inorg. Chem..

[16] Deka N., Sahoo S., Kushwaha V., Zaręba J. K., Boomishankar R. (2025). A Highly Electrostrictive A_2_BX_4_-Type Hybrid 2D Perovskite Ferroelectric and the Utility of Its Piezoelectric
Nanogenerator in Wireless Mat-Sensor Technology. ACS Mater. Lett..

[17] Zhang H. Y., Zhang Z. X., Song X. J., Chen X. G., Xiong R. G. (2020). Two-Dimensional
Hybrid Perovskite Ferroelectric Induced by Perfluorinated Substitution. J. Am. Chem. Soc..

[18] Li J. Y., Wang C. F., Wu H., Liu L., Xu Q. L., Ye S. Y., Tong L., Chen X., Gao Q., Hou Y. L., Wang F. M., Tang J., Chen L. Z., Zhang Y. (2021). Eco-Friendly and Highly Efficient Light-Emission Ferroelectric Scintillators
by Precise Molecular Design. Adv. Funct. Mater..

[19] Ji L. Y., Zhou J. S., Liu S. Y., Qin Y., Lv H. P., Chen X. G. (2025). Effective Enhancement of Mechanical Properties via
H/F Substitution in 3D Cyanide Hybrid Perovskites. Chem. Commun..

[20] Shi J., Gao Y., Gao X., Zhang Y., Zhang J., Jing X., Shao M. (2019). Fluorinated
Low-Dimensional Ruddlesden–Popper Perovskite Solar
Cells with over 17% Power Conversion Efficiency and Improved Stability. Adv. Mater..

[21] Muddam R. S., Jagadamma L. K. (2025). Ferroelectric
Polarization in 2D Halide Hybrid Perovskites:
Influence on Bulk Crystals, Thin Films, and Applications. J. Mater. Chem. C.

[22] Reichenbächer K., Süss H. I., Hulliger J. (2005). Fluorine in Crystal Engineering –
“the Little Atom That Could. Chem. Soc.
Rev..

[23] Pérez-Torralba M., García M. Á., López C., Torralba M. C., Torres M. R., Claramunt R. M., Elguero J. (2014). Structural Investigation of Weak Intermolecular Interactions
(Hydrogen and Halogen Bonds) in Fluorine-Substituted Benzimidazoles. Cryst. Growth Des..

[24] Pavan M. S., Prasad K. D., Row T. N. G. (2013). Halogen
Bonding in Fluorine: Experimental
Charge Density Study on Intermolecular F···F and F···S
Donor–Acceptor Contacts. Chem. Commun..

[25] Janjić G. V., Jelić S. T., Trišović N. P., Popović D. M., Dordević I. S., Milčić M. K. (2020). New Theoretical
Insight into Fluorination and Fluorine–Fluorine Interactions
as a Driving Force in Crystal Structures. Cryst.
Growth Des..

[26] Metrangolo P., Murray J. S., Pilati T., Politzer P., Resnati G., Terraneo G. (2011). Fluorine-Centered Halogen Bonding: A Factor in Recognition
Phenomena and Reactivity. Cryst. Growth Des..

[27] Scheiner S. (2025). Testing the
Reality of F··F Halogen Bonds. Chem.
Phys. Lett..

[28] Rigaku Oxford Diffraction . CrysAlisPro Software System, Version 1.171.42.93a,; Rigaku Corporation: OXford, U.K., 2023.

[29] Sheldrick G. M. (2015). SHELXT
- Integrated Space-Group and Crystal-Structure Determination. Acta Crystallogr., Sect. A: Found. Adv..

[30] Sheldrick G. M. (2015). Crystal
Structure Refinement with SHELXL. Acta Crystallogr,.
Sect. C: Struct. Chem..

[31] Dolomanov O. V., Bourhis L. J., Gildea R. J., Howard J. A. K., Puschmann H. (2009). OLEX2: A Complete
Structure Solution, Refinement and Analysis Program. J. Appl. Crystallogr..

[32] Brandenburg, K. ; Putz, H. Diamond 3.2k – Crystal and Molecular Structure Visualization 102 Crystal Impact: Kreuzherrenstr: Germany; 2014. https://www.crystalimpact.de/diamond.

[33] Spackman P. R., Turner M. J., McKinnon J. J., Wolff S. K., Grimwood D. J., Jayatilaka D., Spackman M. A. (2021). CrystalExplorer: A Program for Hirshfeld
Surface Analysis, Visualization and Quantitative Analysis of Molecular
Crystals. J. Appl. Crystallogr..

[34] M, J. ; Frisch, G. W. ; Trucks, H. B. ; Schlegel, G. E. ; Scuseria, M. A. ; Robb, J. R. ; Cheeseman, J. A. ; Montgomery, T., Jr. ; Vreven, K. N. ; Kudin, J. C. ; Burant, J. M. Gaussian 03, Revision A.1; Gaussian, Inc.: Pittsburgh PA, 2003.

[35] Becke A. D. (1996). Density-Functional
Thermochemistry. IV. A New Dynamical Correlation Functional and Implications
for Exact-Exchange Mixing. J. Chem. Phys..

[36] Calais J.-L. (1993). Density-Functional
Theory of Atoms and Molecules. R.G. Parr and W. Yang, Oxford University
Press, New York, Oxford, 1989. IX + 333 Pp. Price £45.00. Int. J. Quantum Chem..

[37] McLean A. D., Chandler G. S. (1980). Contracted Gaussian
Basis Sets for Molecular Calculations.
I. Second Row Atoms, Z = 11–18. J. Chem.
Phys..

[38] Krishnan R., Binkley J. S., Seeger R., Pople J. A. (1980). Self-Consistent
Molecular Orbital Methods. XX. A Basis Set for Correlated Wave Functions. J. Chem. Phys..

[39] Rostkowska H., Lapinski L., Nowak M. J. (2009). Analysis
of the Normal Modes of Molecules
with D3h Symmetry: Infrared Spectra of Monomeric s-Triazine and Cyanuric
Acid. Vib. Spectrosc..

[40] Zhurko, G. A. ; Zhurko, D. A. Chemcraft Graphical Program of Visualization of Computed Results. http://chemcraftprog.com.

[41] Neese F. (2012). The ORCA Program
System. WIREs Comput. Mol. Sci..

[42] Lu T., Chen F. (2012). Multiwfn: A Multifunctional
Wavefunction Analyzer. J. Comput. Chem..

[43] Lu T. (2024). A Comprehensive
Electron Wavefunction Analysis Toolbox for Chemists, Multiwfn. J. Chem. Phys..

[44] Humphrey W., Dalke A., Schulten K. (1996). VMD: Visual Molecular Dynamics. J. Mol. Graphics.

[45] Williams, T. ; Kelley, C. ; Bersch, C. ; Bröker, H.-B. ; Campbell, J. ; Cunningham, R. ; Denholm, D. ; Elber, G. ; Fearick, R. ; Grammes, C. Gnuplot 5.4 An Interactive Plotting Program. 1986.

[46] Groom C. R., Bruno I. J., Lightfoot M. P., Ward S. C. (2016). The Cambridge Structural
Database. Acta Crystallogr., Sect. B: Struct.
Sci. Cryst. Eng. Mater..

[47] Anzini M., Braile C., Valenti S., Cappelli A., Vomero S., Marinelli L., Limongelli V., Novellino E., Betti L., Giannaccini G., Lucacchini A., Ghelardini C., Norcini M., Makovec F., Giorgi G., Fryer R. I. (2008). Ethyl 8-Fluoro-6-(3-Nitrophenyl)-4H-Imidazo­[1,5-a]­[1,4]­Benzodiazepine-3-Carboxylate
as Novel, Highly Potent, and Safe Antianxiety Agent. J. Med. Chem..

[48] Xu L., Vicic D. A. (2016). Direct Difluoromethylation of Aryl Halides via Base
Metal Catalysis at Room Temperature. J. Am.
Chem. Soc..

[49] Esswein A. J., Veige A. S., Piccoli P. M. B., Schultz A. J., Nocera D. G. (2008). Intramolecular
C–H Bond Activation and Redox Isomerization across Two-Electron
Mixed Valence Diiridium Cores. Organometallics.

[50] Gilday L.
C., Robinson S. W., Barendt T. A., Langton M. J., Mullaney B. R., Beer P. D. (2015). Halogen
Bonding in Supramolecular Chemistry. Chem. Rev..

[51] Johnson E.
R., Keinan S., Mori-Sánchez P., Contreras-García J., Cohen A. J., Yang W. (2010). Revealing Noncovalent Interactions. J. Am.
Chem. Soc..

[52] Ptak M., Dziuk B., Smółka S., Osmólska J., Stefanski M., Łukowiak A., Sieradzki A. (2023). Influence
of Bromination on the Phase Transitions, Structural, Phonon, and Optical
Properties Explored for 2-Bromoethylammonium Bismuth Bromide. J. Phys. Chem. C.

[53] Durig J. R., Ganguly A., Zheng C., Gurigis G. A., Herrebout W. A., van der Veken B. J., Gounev T. K. (2010). Conformational and Structural Studies
of 2-Fluoroethylamine from Temperature Dependent FT-IR Spectra of
Krypton and Xenon Solutions and Ab Initio Calculations. J. Mol. Struct..

[54] Ouasri A., Rhandour A., Dhamelincourt M. C., Dhamelincourt P., Mazzah A. (2003). The Infrared and Raman Spectra of
Ethylammonium Hexafluorosilicate
[C_2_H_5_NH_3_]_2_SiF_6_. Spectrochim. Acta, Part A.

[55] Durig J. R., Klaassen J. J., Panikar S. S., Darkhalil I. D., Ganguly A., Guirgis G. A. (2011). Conformational and
Structural Studies
of 2,2-Difluoroethylamine from Temperature Dependent Infrared Spectra
of Xenon Solution and Ab Initio Calculations. J. Mol. Struct..

[56] Vasylyeva V., Catalano L., Nervi C., Gobetto R., Metrangolo P., Resnati G. (2016). Characteristic Redshift
and Intensity Enhancement as
Far-IR Fingerprints of the Halogen Bond Involving Aromatic Donors. CrystEngComm.

[57] Sun C., Zhong Q. Q., Zhang X., Xiao P. C., Cheng Y., Gao Y. J., Liu G. D., Lei X. W. (2021). A Zero-Dimensional
Hybrid Cadmium Perovskite with Highly Efficient Orange–Red
Light Emission. Inorg. Chem..

[58] Kubelka P. (1948). New Contributions
to the Optics of Intensely Light-Scattering Materials. J. Opt. Soc. Am..

[59] Sun C., He W. L., Liu M. J., Pan W. J., Dong L. F., Chen G., Liu G. D., Lei X. W. (2020). Zero-Dimensional
Hybrid Cd-Based Perovskites with Broadband Bluish White-Light Emissions. Chem. – Asian J..

[60] Mączka M., Stefańska D., Zaręba J. K., Nyk M., Sieradzki A. (2020). Temperature-Dependent
Luminescence and Second-Harmonic Generation of Perovskite-Type Manganese
and Cadmium Dicyanamide Frameworks Templated by Tetrapropylammonium
Cations. J. Alloys Compd..

[61] Wang S., Li L., Sun Z., Ji C., Liu S., Wu Z., Zhao S., Zeb A., Hong M., Luo J. (2017). A Semi-Conductive
Organic–Inorganic Hybrid Emits Pure White Light with an Ultrahigh
Color Rendering Index. J. Mater. Chem. C.

[62] Zhang X., Hua X. N., Huo P., Wang L., Shi X., Cai Z., Zhang Y., Zhang D., Yu S. S. (2024). Second-Order Nonlinear
Switching and Photoluminescence Properties of Cd-Based Hybrid Perovskite
with High-Temperature Phase Transition. Inorg.
Chem..

[63] Sanni A. M., Lavan S. N., Liu Z. F., Rury A. S. (2021). Defect-Induced
Narrowband
Light Emission from a 2D Hybrid Lead Iodide Perovskite. J. Phys. Chem. C.

[64] Li S., Luo J., Liu J., Tang J. (2019). Self-Trapped Excitons in All-Inorganic
Halide Perovskites: Fundamentals, Status, and Potential Applications. J. Phys. Chem. Lett..

[65] Qi Z., Gao H., Zhu X., Lu Z., Zhang X. M. (2022). Blue Light-Excitable
Broadband Yellow Emission in a Zero-Dimensional Hybrid Bismuth Halide
with Type-II Band Alignment. Inorg. Chem..

[66] Pareja-Rivera C., Morán-Muñoz J. A., Gómora-Figueroa A. P., Jancik V., Vargas B., Rodríguez-Hernández J., Solis-Ibarra D. (2022). Optimizing Broadband Emission in 2D Halide Perovskites. Chem. Mater..

[67] Mao L., Guo P., Kepenekian M., Hadar I., Katan C., Even J., Schaller R. D., Stoumpos C. C., Kanatzidis M. G. (2018). Structural
Diversity in White-Light-Emitting Hybrid Lead Bromide Perovskites. J. Am. Chem. Soc..

[68] Chapuis G. (1977). X-ray Study
of the First-order Phase Transition Pcab–Bmab in (CH_3_CH_2_NH_3_)_2_CdCl_4_. Phys. Status Solidi A.

[69] Han X., Zheng Y., Chai S., Chen S., Xu J. (2020). 2D Organic-Inorganic
Hybrid Perovskite Materials for Nonlinear Optics. Nanophotonics.

[70] Mao L., Stoumpos C. C., Kanatzidis M. G. (2019). Two-Dimensional Hybrid Halide Perovskites:
Principles and Promises. J. Am. Chem. soc..

[71] Maqbool S., Sheikh T., Thekkayil Z., Deswal S., Boomishankar R., Nag A., Mandal P. (2021). Third Harmonic Upconversion and Self-Trapped Excitonic
Emission in 1D Pyridinium Lead Iodide. J. Phys.
Chem. C.

[72] Dorenbos P. (2005). Thermal Quenching
of Eu2+ 5d-4f Luminescence in Inorganic Compounds. J. Phys.: Condens. Matter.

[73] Stefańska D., Bondzior B., Vu T. H. Q., Miniajluk-Gaweł N., Dereń P. J. (2020). The Influence
of Morphology and Eu^3+^ Concentration
on Luminescence and Temperature Sensing Behavior of Ba_2_MgWO_6_ Double Perovskite as a Potential Optical Thermometer. J. Alloys Compd..

[74] He Y., Liu S., Yao Z., Zhao Q., Chabera P., Zheng K., Yang B., Pullerits T., Chen J. (2023). Nature of Self-Trapped
Exciton Emission in Zero-Dimensional Cs_2_ZrCl_6_ Perovskite Nanocrystals. J. Phys. Chem. Lett..

[75] Pan F., Li J., Ma X., Nie Y., Liu B., Ye H. (2021). Free and Self-Trapped
Exciton Emission in Perovskite CsPbBr_3_ Microcrystals. RSC Adv..

[76] Liu Y., Yan S., Wang T., He Q., Zhu X., Wang C., Liu D., Wang T., Xu X., Yu X. (2023). Achieving Color-Tunable
Long Persistent Luminescence in Cs_2_CdCl_4_ Ruddlesden-Popper
Phase Perovskites. Angew. Chem., Int. Ed..

