# Supplementary material for: Fluorine-Induced Rigidity and Entropy Effects in Mixed-Halide 2,2-Difluoroethylammonium Cadmium Hybrids
Source: Inorg Chem. 2026 Jun 10;65(24):13428–40. doi: 10.1021/acs.inorgchem.6c01320 (PMC13298908; doi:10.1021/acs.inorgchem.6c01320)
Supplement: Supplementary file 1 [file ic6c01320_si_001.pdf]

*Electronic Supplementary Information*

*for*

# Fluorine-Induced Rigidity and Entropy Effects in Mixed-Halide 2,2-Difluoroethylammonium Cadmium Hybrids

Maciej Ptak,[1*] Dorota A. Kowalska,[1] Szymon Smółłka,[1] Edyta Kucharska,[2] Mariusz Stefanski,[1] Damian Szymański,[1] Anna Ładak,[1] Adam Sieradzki [3]

*[1] Institute of Low Temperature and Structure Research, Polish Academy of Sciences, 50-422 Wrocław, Poland, M.Ptak@intibs.pl [2] Department of Bioorganic Chemistry, Wroclaw University of Economics and Business, 53-345 Wrocław, Poland [3] Department of Experimental Physics, Wrocław University of Science and Technology, 50-370 Wrocław, Poland*

1. **Chemical composition and phase purity – EDS and p-XRD** ........ S2
2. **Thermal studies** ........ S3
3. **Single-crystal XRD studies** ........ S4
4. **NCI calculations** ........ S17
5. **DFT calculations** ........ S19
6. **Vibrational studies** ........ S22
7. **Optical properties** ........ S29

## 1. Chemical composition and phase purity – EDS and p-XRD

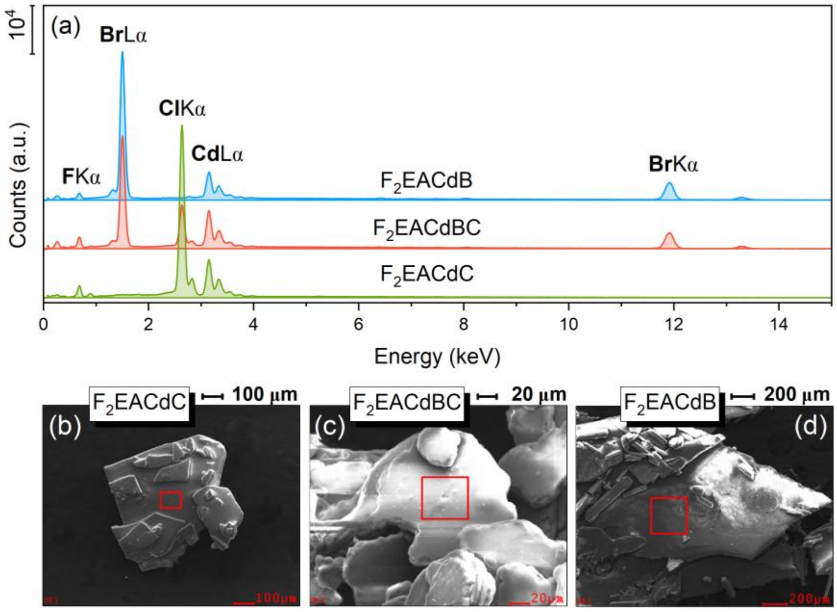


**Figure S1**. The EDS spectra (a) and representative SEM images of **$F_2$EACdC** (b), **$F_2$EACdBC** (c), and **$F_2$EACdB** (d) together with EDS profiles with indicated acquisition area.

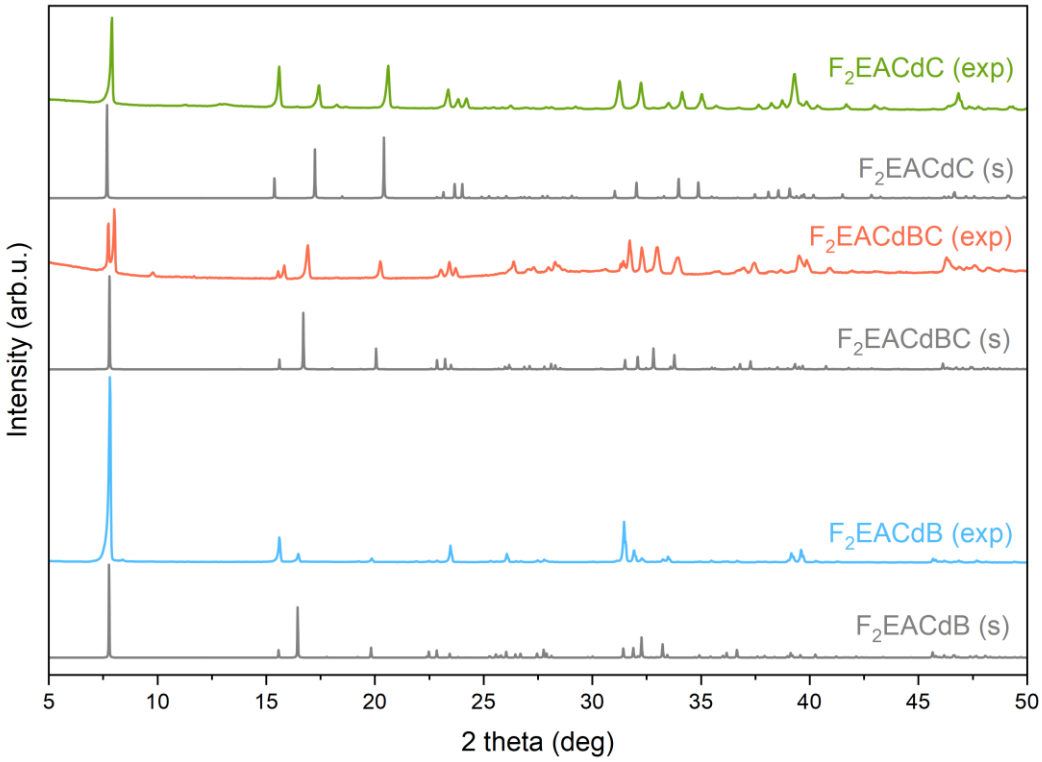


**Figure S2**. The comparison of experimental and calculated powder XRD patterns for **$F_2$EACdC**, **$F_2$EACdBC**, and **$F_2$EACdB**; s, simulated; exp, experimental.

## 2. Thermal studies

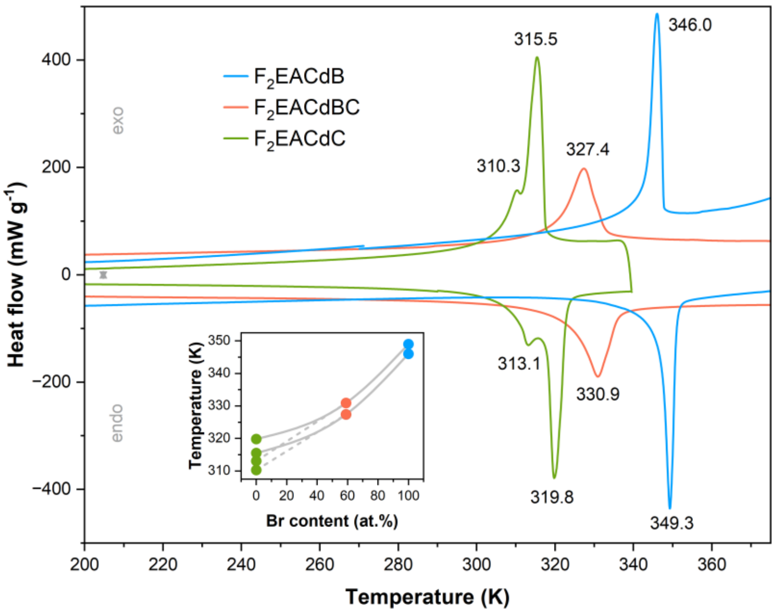


**Figure S3**. Comparison of DSC curves in mW g$^{-1}$ obtained for 10.43 mg of $F_2EACdC$, 4.65 mg of $F_2EACdB$, and 16.54 mg of $F_2EACdBC$; heating/cooling rate was 5 Kmin$^{-1}$. The inset demonstrates the changes in PT temperature as a function of Br concentration.

## 3. Single-crystal XRD studies

**Table S1.** The diffraction experimental details. CCDC deposition numbers: 2479793-2479798.

| Compound | **$F_2$EACdC** | | **$F_2$EACdBC** | | **$F_2$EACdB** | |
|---|---|---|---|---|---|---|
| Phase | **LT** | **HT** | **LT** | **HT** | **LT** | **HT** |
| ***Crystal data*** | | | | | | |
| Chemical formula | $2(C_2H_6F_2N)\cdot CdCl_4$ | | $2(C_2H_6F_2N)\cdot CdBr_{2.48}Cl_{1.52}$ | | $2(C_2H_6F_2N)\cdot CdBr_4$ | |
| $M_r$ | 418.36 | | 528.62 | | 596.16 | |
| Crystal system, space group | Orthorhombic *Pbca* (no. 61) | Orthorhombic *Cmce* (no. 64) | Orthorhombic *Pbca* (no. 61) | Orthorhombic *Cmce* (no. 64) | Orthorhombic *Pbca* (no. 61) | Orthorhombic *Cmce* (no. 64) |
| $T$ (K) | 295 | 340 | 295 | 340 | 295 | 350 |
| $a$, $b$, $c$ (Å) | 7.513(3), 7.405(3), 23.036(5) | 7.456(3), 23.415(5), 7.557(3) | 7.776(3), 7.653(3), 22.699(5) | 7.721(3), 22.941(5), 7.838(3) | 7.905(3), 7.780(3), 22.756(5) | 7.886(3), 22.709(5), 8.014(3) |
| $V$ (Å$^3$) | 1281.6 (8) | 1319.3 (8) | 1350.8 (8) | 1388.3 (8) | 1399.5 (8) | 1435.2 (8) |
| Z | 4 | | 4 | | 4 | |
| Calc. density (g cm$^{-3}$) | 2.168 | 2.106 | 2.599 | 2.530 | 2.830 | 2.759 |
| $\mu$ (mm$^{-1}$) | 2.56 | 2.48 | 9.27 | 9.03 | 13.00 | 12.68 |
| Crystal size (mm) | 0.51 × 0.24 × 0.05 | | 0.28 × 0.27 × 0.04 | | 0.18 × 0.25 × 0.04 | |
| ***Data collection*** | | | | | | |
| Refl. measured/ unique/ observed [$I > 2\sigma(I)$] | 5382/ 1510/ 995 | 3199/ 847/ 638 | 5821/ 1635/ 1192 | 4164/ 901/ 1192 | 6184/ 1424/ 1125 | 3549/ 933/ 587 |
| $R_{int}$ | 0.033 | 0.025 | 0.030 | 0.025 | 0.036 | 0.032 |
| $(\sin\theta/\lambda)_{max}$ (Å$^{-1}$) | 0.667 | 0.666 | 0.687 | 0.676 | 0.625 | 0.687 |
| ***Refinement*** | | | | | | |
| $R[F^2 > 2\sigma(F^2)]$, $wR(F^2)$, $S$ | 0.045, 0.115, 1.07 | 0.044, 0.140, 1.12 | 0.037, 0.083, 1.09 | 0.034, 0.100, 1.05 | 0.037, 0.095, 1.11 | 0.040, 0.116, 1.03 |
| Data/ parameters/ restraints | 1510/ 71/ 1 | 847/ 55/ 21 | 1635/ 95/ 1 | 901/ 57/ 9 | 1424/ 71/ 0 | 933/ 56/ 5 |
| $\Delta\rho_{max}$, $\Delta\rho_{min}$ (e Å$^{-3}$) | 0.83, −0.64 | 0.79, −0.69 | 0.56, −1.42 | 0.45, −0.53 | 1.09, −0.73 | 0.86, −0.86 |

**Table S2**. Selected F···F distances [lower than twice the van der Waals radius of F (2.94 Å)].

| Phase | C—F···F | F···F (Å) | C—F···F (°) |
|---|---|---|---|
| **$F_2$EACdC LT (295 K)** | C2—F1···F2$^{viii}$ | 2.851 | 144.6 |
| **$F_2$EACdC HT (340 K)** | C2—F1···F2$^{ix}$ | 2.741 | 138.0 |
| | C2—F1···F2$^{x}$ | 2.707 | 133.9 |
| | C2—F1···F2$^{xi}$ | 2.523 | 162.6 |
| **$F_2$EACdBC LT (295 K)** | C2—F1···F2$^{viii}$ | 2.696 | 151.9 |
| | C2—F2A···F1A$^{xii}$ | 2.656 | 145.6 |
| | C2—F2A···F1A$^{xii}$ | 2.548 | 129.5 |
| **$F_2$EACdBC HT (340 K)** | C2—F1···F2$^{ix}$ | 2.736 | 141.1 |
| | C2—F1···F2$^{x}$ | 2.677 | 125.3 |
| | C2—F1···F2$^{xi}$ | 2.454 | 166.1 |
| **$F_2$EACdB LT (295 K)** | C2—F1···F2$^{viii}$ | 2.813 | 154.7 |
| **$F_2$EACdB HT (350 K)** | C2—F1···F2$^{ix}$ | 2.634 | 145.6 |
| | C2—F1···F2$^{x}$ | 2.641 | 127.4 |
| | C2—F1···F2$^{xi}$ | 2.465 | 168.3 |

Symmetry codes: (viii) *x*-1/2, *y*, -*z*+1/2; (ix) *x*, -*y*+1/2, *z*-1/2; (x) -*x*+3/2, -*y*+1/2, -*z*+1; (xi) -*x*+3/2, *y*, -*z*+3/2; (xii) -*x*+1, *y*+1/2, -*z*+1/2.

**Table S3**. Selected hydrogen-bond parameters (Å, °).

| Phase | *D*—H···*A* | *D*—H (Å) | H···*A* (Å) | *D*···*A* (Å) | *D*—H···*A* (°) |
|---|---|---|---|---|---|
| **$F_2$EACdC LT (295 K)** | N1—H1A···Cl1$^{i}$ | 0.89 | 2.46 | 3.265(6) | 150.5 |
| | N1—H1B···Cl2$^{ii}$ | 0.89 | 2.47 | 3.337(6) | 164.2 |
| | N1—H1C···Cl2 | 0.89 | 2.34 | 3.225(5) | 171.7 |
| | C1—H1D···Cl2$^{iii}$ | 0.97 | 2.78 | 3.696(8) | 157.0 |
| | C1—H1E···Cl2$^{iv}$ | 0.97 | 2.80 | 3.766(8) | 171.4 |
| | C2—H2···F1$^{v}$ | 0.98 | 2.47 | 3.068(10) | 118.9 |
| **$F_2$EACdC HT (340 K)** | N1—H1A···Cl1$^{vi}$ | 0.87 | 2.62 | 3.445(8) | 158.7 |
| | N1—H1B···Cl2 | 0.89 | 2.35 | 3.216(8) | 162.9 |
| | N1—H1C···Cl2$^{v}$ | 0.90 | 2.87 | 3.736(2) | 163.8 |
| | C1—H1D···Cl2$^{vii}$ | 0.91 | 2.93 | 3.838(19) | 175.3 |
| | C1—H1E···Cl2$^{viii}$ | 0.91 | 2.65 | 3.534(17) | 163.5 |
| **$F_2$EACdBC LT (295 K)** | N1—H1A···Br1\|Cl1$^{i}$ | 0.89 | 2.56 | 3.353(5) | 148.8 |
| | N1—H1B···Br2\|Cl2$^{ii}$ | 0.89 | 2.59 | 3.455(5) | 163.9 |
| | N1—H1C···Br2\|Cl2 | 0.89 | 2.46 | 3.339(5) | 171.1 |
| | C1—H1D···Br2\|Cl2$^{iii}$ | 0.97 | 2.90 | 3.823(7) | 158.9 |
| | C1—H1E···Br2\|Cl2$^{iv}$ | 0.97 | 2.92 | 3.888(7) | 174.2 |
| | C2—H2···F1$^{v}$ | 0.98(2) | 2.54(7) | 3.10(2) | 116(5) |
| | C2—H2···F1A$^{v}$ | 0.98(2) | 2.24(6) | 3.02(3) | 136(6) |
| **$F_2$EACdBC HT (340 K)** | N1—H1A···Br1\|Cl1$^{vi}$ | 0.88 | 2.68 | 3.512(8) | 159.3 |
| | N1—H1B···Br2\|Cl2 | 0.93 | 2.43 | 3.331(8) | 163.5 |
| | N1—H1C···Br2\|Cl2$^{v}$ | 0.93 | 2.98 | 3.876(2) | 162.7 |
| | C1—H1D···Br2\|Cl2$^{vii}$ | 0.94 | 3.07 | 4.004(18) | 175.5 |
| | C1—H1E···Br2\|Cl2$^{viii}$ | 0.94 | 2.71 | 3.627(16) | 165.6 |
| **$F_2$EACdB LT (295 K)** | N1—H1C···Br1$^{i}$ | 0.89 | 2.61 | 3.396(7) | 148.1 |
| | N1—H1A···Br2$^{ii}$ | 0.89 | 2.63 | 3.494(6) | 164.2 |
| | N1—H1B···Br2 | 0.89 | 2.51 | 3.386(6) | 170.6 |
| | C1—H1D···Br2$^{iii}$ | 0.97 | 2.98 | 3.903(9) | 159.4 |
| | C1—H1E···Br2$^{iv}$ | 0.97 | 3.00 | 3.967(9) | 174.6 |
| | C2—H2···F1$^{v}$ | 0.98 | 2.55 | 3.070(12) | 113.4 |
| **$F_2$EACdB HT (350 K)** | N1—H1B···Br1$^{vi}$ | 0.89 | 2.77 | 3.568(9) | 149.4 |
| | N1—H1A···Br2 | 0.89 | 2.60 | 3.387(11) | 148.4 |
| | N1—H1C···Br2$^{v}$ | 0.89 | 3.07 | 3.959(2) | 179.5 |
| | C1—H1D···Br2$^{vii}$ | 0.97 | 3.18 | 4.14(2) | 171.4 |
| | C1—H1E···Br2$^{viii}$ | 0.97 | 2.72 | 3.64(2) | 157.8 |

Symmetry codes: (i) *x*+1/2, -*y*+1/2, -*z*; (ii) -*x*+1/2, *y*+1/2, *z*; (iii) -*x*+1/2, *y*-1/2, *z*; (iv) *x*+1, *y*, *z*; (v) *x*+1/2, *y*, -*z*+1/2; (vi) -*x*+1/2, -*y*, *z*+1/2; (vii) *x*, *y*, *z*+1; (viii) *x*-1/2, *y*, -*z*+1/2.

**Table S4**. Comparison of octahedral parameters: polyhedral volume ($V_p$), bond length distortion index ($\Delta$), bond angle variance ($\sigma^2$), calculated with the use of VESTA program.[S1]

| Compound | Phase | T (K) | $V_p$ (Å$^3$) | $\Delta$ | $\sigma^2$ (deg$^2$) |
|---|---|---|---|---|---|
| **$F_2$EACdC** | **LT** | 295 | 24.327 | 0.0252 | 1.665 |
| | **HT** | 340 | 23.945 | 0.0262 | 1.010 |
| **$F_2$EACdBC** | **LT** | 295 | 27.122 | 0.0222 | 1.591 |
| | **HT** | 340 | 26.842 | 0.0240 | 1.250 |
| **$F_2$EACdB** | **LT** | 295 | 28.545 | 0.0246 | 1.661 |
| | **HT** | 350 | 28.331 | 0.0294 | 1.318 |

[S1] K. Momma and F. Izumi, VESTA 3 for three-dimensional visualization of crystal, volumetric and morphology data, *J. Appl. Crystallogr*. 44, **2011**, 1272-1276.

**Table S5**. Selected geometric parameters (Å, °) of **$F_2$EACdC**.

| $F_2$EACdC – LT (295 K) | | $F_2$EACdC – HT (340 K) | |
|---|---|---|---|
| Cd1—Cl1$^{i}$ | 2.6913(15) | Cd1—Cl1 | 2.6717(8) |
| Cd1—Cl1$^{ii}$ | 2.6913(15) | Cd1—Cl1$^{v}$ | 2.6717(8) |
| Cd1—Cl1 | 2.6767(15) | Cd1—Cl1$^{vi}$ | 2.6717(8) |
| Cd1—Cl1$^{iii}$ | 2.6766(15) | Cd1—Cl1$^{vii}$ | 2.6717(8) |
| Cd1—Cl2 | 2.5345(17) | Cd1—Cl2 | 2.517(3) |
| Cd1—Cl2$^{iii}$ | 2.5346(17) | Cd1—Cl2$^{iii}$ | 2.517(3) |
| N1—C1 | 1.487(9) | N1—C1 | 1.36(2) |
| C1—C2 | 1.448(10) | C1—C2 | 1.405(16) |
| C2—F1 | 1.268(9) | C2—F1 | 1.260(15) |
| C2—F2 | 1.383(9) | C2—F2 | 1.287(14) |
| | | | |
| Cl1—Cd1—Cl1$^{iii}$ | 180.0 | Cl1—Cd1—Cl1$^{v}$ | 180.0 |
| Cl1$^{ii}$—Cd1—Cl1$^{i}$ | 180.0 | Cl1$^{vii}$—Cd1—Cl1$^{vi}$ | 180.0 |
| Cl2$^{iii}$—Cd1—Cl2 | 180.0 | Cl2$^{v}$—Cd1—Cl2 | 180.0 |
| Cl1$^{iii}$—Cd1—Cl1$^{i}$ | 91.71(3) | Cl1$^{vii}$—Cd1—Cl1$^{v}$ | 91.52(3) |
| Cl1—Cd1—Cl1$^{ii}$ | 91.71(3) | Cl1—Cd1—Cl1$^{vi}$ | 91.52(3) |
| Cl1—Cd1—Cl1$^{i}$ | 88.29(3) | Cl1—Cd1—Cl1$^{vii}$ | 88.48(3) |
| Cl1$^{iii}$—Cd1—Cl1$^{ii}$ | 88.29(3) | Cl1$^{vi}$—Cd1—Cl1$^{v}$ | 88.48(3) |
| Cl2$^{iii}$—Cd1—Cl1$^{ii}$ | 91.22(5) | Cl2$^{v}$—Cd1—Cl1$^{vi}$ | 90.49(7) |
| Cl2—Cd1—Cl1$^{i}$ | 91.22(5) | Cl2—Cd1—Cl1$^{vii}$ | 90.49(7) |
| Cl2—Cd1—Cl1$^{iii}$ | 90.38(5) | Cl2—Cd1—Cl1$^{v}$ | 89.51(7) |
| Cl2$^{iii}$—Cd1—Cl1 | 90.38(5) | Cl2$^{v}$—Cd1—Cl1 | 89.51(7) |
| Cl2—Cd1—Cl1 | 89.62(5) | Cl2—Cd1—Cl1 | 90.49(7) |
| Cl2$^{iii}$—Cd1—Cl1$^{iii}$ | 89.62(5) | Cl2$^{v}$—Cd1—Cl1$^{v}$ | 90.49(7) |
| Cl2—Cd1—Cl1$^{ii}$ | 88.78(5) | Cl2—Cd1—Cl1$^{vi}$ | 89.51(7) |
| Cl2$^{iii}$—Cd1—Cl1$^{i}$ | 88.78(5) | Cl2$^{v}$—Cd1—Cl1$^{vii}$ | 89.51(7) |
| Cd1—Cl1—Cd1$^{iv}$ | 158.58(7) | Cd1—Cl1—Cd $^{viii}$ | 166.82(12) |
| C2—C1—N1 | 114.6(6) | C2—C1—N1 | 126.0(13) |
| F1—C2—C1 | 111.6(8) | F1—C2—C1 | 114.4(16) |
| F2—C2—C1 | 107.0(7) | F2—C2—C1 | 108.3(16) |
| F1—C2—F2 | 105.4 (8) | F1—C2—F2 | 90.1 (10) |
| | | | |
| N1—C1—C2—F1 | 48.3(10) | N1—C1—C2—F1 | 10(3) |
| N1—C1—C2—F2 | -66.5(9) | N1—C1—C2—F2 | -89(3) |

Symmetry codes: (i) *x*+1/2, -*y*+1/2, -*z*; (ii) -*x*+1/2, *y*+1/2, *z*; (iii) -*x*+1/2, *y*-1/2, *z*; (iv) *x*+1, *y*, *z*; (v) *x*+1/2, *y*, -*z*+1/2; (vi) -*x*+1/2, -*y*, *z*+1/2; (vii) *x*, *y*, *z*+1; (viii) *x*-1/2, *y*, -*z*+1/2.

**Table S6**. Selected geometric parameters (Å, °) of **$F_2$EACdBC**.

| $F_2$EACdBC – LT (295 K) | | $F_2$EACdBC – HT (340 K) | |
|---|---|---|---|
| Cd1—Br1\|Cl1$^{i}$ | 2.7861(10) | Cd1—Br1\|Cl1 | 2.7707(8) |
| Cd1—Br1\|Cl1$^{ii}$ | 2.7861(10) | Cd1—Br1\|Cl1$^{v}$ | 2.7707(8) |
| Cd1—Br1\|Cl1 | 2.7669(10) | Cd1—Br1\|Cl1$^{vi}$ | 2.7707(8) |
| Cd1—Br1\|Cl1$^{iii}$ | 2.7669(10) | Cd1—Br1\|Cl1$^{vii}$ | 2.7707(8) |
| Cd1—Br2\|Cl1 | 2.6404(9) | Cd1—Br2\|Cl2 | 2.6238(12) |
| Cd1—Br2\|Cl1$^{iii}$ | 2.6404(9) | Cd1—Br2\|Cl2$^{iii}$ | 2.6239(12) |
| N1—C1 | 1.480(8) | N1—C1 | 1.359(18) |
| C1—C2 | 1.448(11) | C1—C2 | 1.409(15) |
| C2—F1 / F1A | 1.304(15) /1.52(4) | C2—F1 | 1.313(13) |
| C2—F2 / F2A | 1.433(16) /1.11(3) | C2—F2 | 1.352(13) |
| | | | |
| Br1\|Cl1—Cd1—Br1\|Cl1$^{iii}$ | 180.0 | Br1\|Cl1—Cd1—Br1\|Cl1$^{v}$ | 180.0 |
| Br1\|Cl1$^{ii}$—Cd1—Br1\|Cl1$^{i}$ | 180.0 | Br1\|Cl1$^{vii}$—Cd1—Br1\|Cl1$^{vi}$ | 180.0 |
| Br2\|Cl2$^{iii}$—Cd1—Br2\|Cl2 | 180.0 | Br2\|Cl2$^{v}$—Cd1—Br2\|Cl2 | 180.0 |
| Br1\|Cl1$^{iii}$—Cd1—Br1\|Cl1$^{i}$ | 91.88(3) | Br1\|Cl1$^{vii}$—Cd1—Br1\|Cl1$^{v}$ | 91.68(3) |
| Br1\|Cl1—Cd1—Br1\|Cl1$^{ii}$ | 91.88(3) | Br1\|Cl1—Cd1—Br1\|Cl1$^{vi}$ | 91.68(3) |
| Br1\|Cl1—Cd1—Br1\|Cl1$^{i}$ | 88.12(3) | Br1\|Cl1—Cd1—Br1\|Cl1$^{vii}$ | 88.32(3) |
| Br1\|Cl1$^{iii}$—Cd1—Br1\|Cl1$^{ii}$ | 88.12(3) | Br1\|Cl1$^{vi}$—Cd1—Br1\|Cl1$^{v}$ | 88.32(3) |
| Br2\|Cl2$^{iii}$—Cd1—Br1\|Cl1$^{ii}$ | 90.91(2) | Br2\|Cl2$^{v}$—Cd1—Br1\|Cl1$^{vi}$ | 90.55(3) |
| Br2\|Cl2—Cd1—Br1\|Cl1$^{i}$ | 90.91(2) | Br2\|Cl2—Cd1—Br1\|Cl1$^{vii}$ | 90.55(3) |
| Br2\|Cl2—Cd1—Br1\|Cl1$^{iii}$ | 89.92(2) | Br2\|Cl2—Cd1—Br1\|Cl1$^{v}$ | 89.45(3) |
| Br2\|Cl2$^{iii}$—Cd1—Br1\|Cl1 | 89.92(2) | Br2\|Cl2$^{v}$—Cd1—Br1\|Cl1 | 89.45(3) |
| Br2\|Cl2—Cd1—Br1\|Cl1 | 90.08(2) | Br2\|Cl2—Cd1—Br1\|Cl1 | 90.55(3) |
| Br2\|Cl2$^{iii}$—Cd1—Br1\|Cl1$^{iii}$ | 90.08(2) | Br2\|Cl2$^{v}$—Cd1—Br1\|Cl1$^{v}$ | 90.55(3) |
| Br2\|Cl2—Cd1—Br1\|Cl1$^{ii}$ | 89.09(2) | Br2\|Cl2—Cd1—Br1\|Cl1$^{vi}$ | 89.45(3) |
| Br2\|Cl2$^{iii}$—Cd1—Br1\|Cl1$^{i}$ | 89.09(2) | Br2\|Cl2$^{v}$—Cd1—Br1\|Cl1$^{vii}$ | 89.45(3) |
| Cd1—Br1\|Cl1—Cd1$^{iv}$ | 158.45(3) | Cd1—Br1\|Cl1—Cd$^{viii}$ | 166.52(4) |
| C2—C1—N1 | 114.4(6) | C2—C1—N1 | 128.8(12) |
| F1 / F1A—C2—C1 | 115.7(9) / 94(2) | F1—C2—C1 | 112.2(13) |
| F2 / F2A—C2—C1 | 102.9(10) / 125(2) | F2—C2—C1 | 101.8(13) |
| F1 / F1A—C2—F2 / F2A | 99.0(13) / 107(3) | F1—C2—F2 | 90.3(9) |
| | | | |
| N1—C1—C2—F1 / F1A | 37.2(19) / 77(2) | N1—C1—C2—F1 | 14(2) |
| N1—C1—C2—F2 / F2A | -69.5(9) / -36(6) | N1—C1—C2—F2 | -81(2) |

Symmetry codes: (i) *x*+1/2, -*y*+1/2, -*z*; (ii) -*x*+1/2, *y*+1/2, *z*; (iii) -*x*+1/2, *y*-1/2, *z*; (iv) *x*+1, *y*, *z*; (v) *x*+1/2, *y*, -*z*+1/2; (vi) -*x*+1/2, -*y*, *z*+1/2; (vii) *x*, *y*, *z*+1; (viii) *x*-1/2, *y*, -*z*+1/2.

**Table S7**. Selected geometric parameters (Å, °) of **$F_2$EACdB**.

| $F_2$EACdB – LT (295 K) | | $F_2$EACdB – HT (350 K) | |
|---|---|---|---|
| Cd1—Br1$^{i}$ | 2.8384(10) | Cd1—Br1 | 2.8327(8) |
| Cd1—Br1$^{ii}$ | 2.8384(10) | Cd1—Br1$^{v}$ | 2.8327(8) |
| Cd1—Br1 | 2.8208(10) | Cd1—Br1$^{vi}$ | 2.8327(8) |
| Cd1—Br1$^{iii}$ | 2.8208(10) | Cd1—Br1$^{vii}$ | 2.8327(8) |
| Cd1—Br2 | 2.6758(10) | Cd1—Br2 | 2.6495(13) |
| Cd1—Br2$^{iii}$ | 2.6758(10) | Cd1—Br2$^{iii}$ | 2.6495(13) |
| N1—C1 | 1.479(11) | N1—C1 | 1.36(2) |
| C1—C2 | 1.442(13) | C1—C2 | 1.39(3) |
| C2—F1 | 1.283(11) | C2—F1 | 1.368(14) |
| C2—F2 | 1.386(11) | C2—F2 | 1.400(14) |
| | | | |
| Br1—Cd1—Br1$^{iii}$ | 180.0 | Br1—Cd1—Br1$^{v}$ | 180.0 |
| Br1$^{ii}$—Cd1—Br1$^{i}$ | 180.00(4) | Br1$^{vii}$—Cd1—Br1$^{vi}$ | 180.00(5) |
| Br2$^{iii}$—Cd1—Br2 | 180.0 | Br2$^{v}$—Cd1—Br2 | 180.0 |
| Br1$^{iii}$—Cd1—Br1$^{i}$ | 91.96(3) | Br1$^{vii}$—Cd1—Br1$^{v}$ | 91.79(3) |
| Br1—Cd1—Br1$^{ii}$ | 91.96(3) | Br1—Cd1—Br1$^{vi}$ | 91.79(3) |
| Br1—Cd1—Br1$^{i}$ | 88.04(3) | Br1—Cd1—Br1$^{vii}$ | 88.21(3) |
| Br1$^{iii}$—Cd1—Br1$^{ii}$ | 88.04(3) | Br1$^{vi}$—Cd1—Br1$^{v}$ | 88.21(3) |
| Br2$^{iii}$—Cd1—Br1$^{ii}$ | 90.79(2) | Br2$^{v}$—Cd1—Br1$^{vi}$ | 90.45(3) |
| Br2—Cd1—Br1$^{i}$ | 90.79(2) | Br2—Cd1—Br1$^{vii}$ | 90.45(3) |
| Br2—Cd1—Br1$^{iii}$ | 89.65(2) | Br2—Cd1—Br1$^{v}$ | 89.55(3) |
| Br2$^{iii}$—Cd1—Br1 | 89.65(2) | Br2$^{v}$—Cd1—Br1 | 89.55(3) |
| Br2—Cd1—Br1 | 90.35(2) | Br2—Cd1—Br1 | 90.45(3) |
| Br2$^{iii}$—Cd1—Br1$^{iii}$ | 90.35(2) | Br2$^{v}$—Cd1—Br1$^{v}$ | 90.45(3) |
| Br2—Cd1—Br1$^{ii}$ | 89.21(2) | Br2—Cd1—Br1$^{vi}$ | 89.55(3) |
| Br2$^{iii}$—Cd1—Br1$^{i}$ | 89.21(2) | Br2$^{v}$—Cd1—Br1$^{vii}$ | 89.55(3) |
| Cd1—Br1—Cd1$^{iv}$ | 157.01(3) | Cd1—Br1—Cd$^{viii}$ | 165.75(5) |
| C2—C1—N1 | 114.3(7) | C2—C1—N1 | 105.5(18) |
| F1—C2—C1 | 112.6(9) | F1—C2—C1 | 114.0(18) |
| F2—C2—C1 | 107.9(9) | F2—C2—C1 | 105.5(18) |
| F1—C2—F2 | 101.7(10) | F1—C2—F2 | 93.1(10) |
| | | | |
| N1—C1—C2—F1 | 46.7(12) | N1—C1—C2—F1 | 23(3) |
| N1—C1—C2—F2 | -64.7(10) | N1—C1—C2—F2 | -78(3) |

Symmetry codes: (i) $x$+1/2, -$y$+1/2, -$z$; (ii) -$x$+1/2, $y$+1/2, $z$; (iii) -$x$+1/2, $y$-1/2, $z$; (iv) $x$+1, $y$, $z$; (v) $x$+1/2, $y$, -$z$+1/2; (vi) -$x$+1/2, -$y$, $z$+1/2; (vii) $x$, $y$, $z$+1; (viii) $x$-1/2, $y$, -$z$+1/2.

**Table S8**. Contributions of individual intermolecular contacts to the Hirshfeld surface area.

| Compound | Phase | T (K) | F···F (%) | H···H (%) | H···F / F···H (%) | H···Br/Cl (%) |
|---|---|---|---|---|---|---|
| $F_2$EACdC | LT | 295 | 8.0 | 8.0 | 42.4 | 38.6 |
| | HT | 340 | 24.7 | 20.8 | 13.9 | 37.4 |
| $F_2$EACdBC | LT | 295 | 15.1 | 6.5 | 37.0 | 39.2 |
| | HT | 340 | 26.0 | 17.8 | 14.9 | 39.9 |
| $F_2$EACdB | LT | 295 | 8.0 | 6.9 | 42.0 | 40.9 |
| | HT | 350 | 26.3 | 19.5 | 16.4 | 38.1 |

**Table S9**. Volumes of void domains (in Å$^3$) and their contribution to the unit cell for the LT and HT phases, calculated in CrystalExplorer with an isovalue of 0.002 e au$^{-3}$.

| Phase | $F_2$EACdC | $F_2$EACdBC | $F_2$EACdB |
|---|---|---|---|
| HT | 62.75 | 42.00 | 38.19 |
| | (4.76 %) | (3.03 %) | (2.71 %) |
| LT | 110.24 | 68.86 | 118.42 |
| | (8.60 %) | (5.10 %) | (8.46 %) |

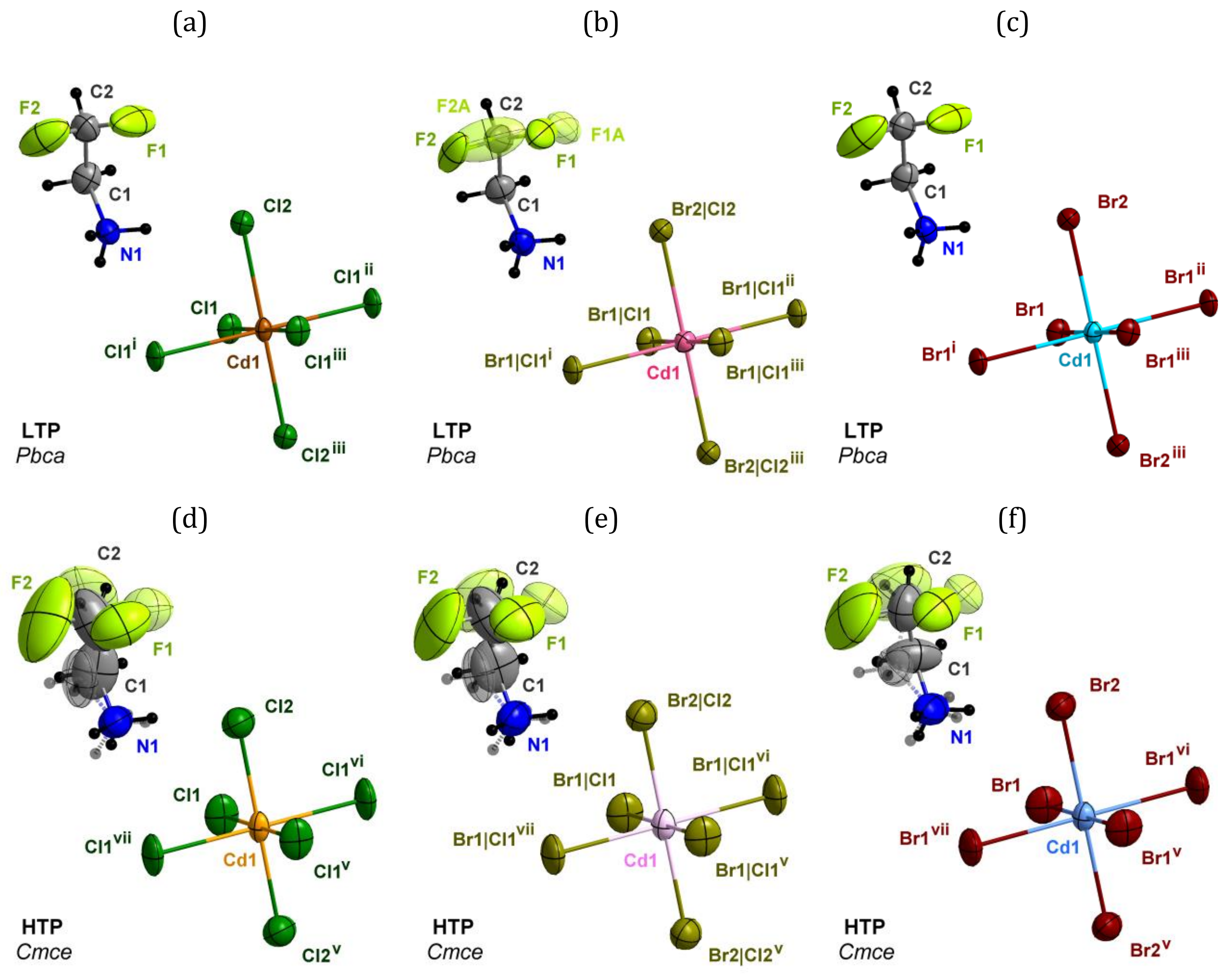


**Figure S4**. The asymmetric unit of **$F_2$EACdC**, **$F_2$EACdBC**, and **$F_2$EACdB** showing the atom numbering scheme for two phases: (a-c) the LT phase at 295 K, and (d-f) the HT phase with a disordered cation (the disordered part is shown in transparent color and with dashed bonds).

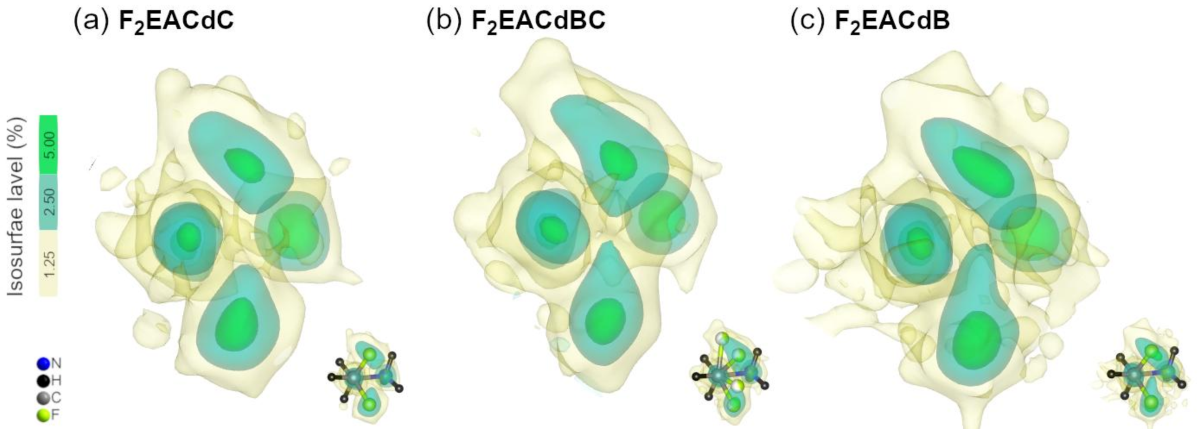


**Figure S5**. Comparison of observed electron density of the $F_2EA^+$ cation in the three studied structures in the LT phase, viewed along the C–C bond direction. For clarity, only positive density isosurfaces are shown.

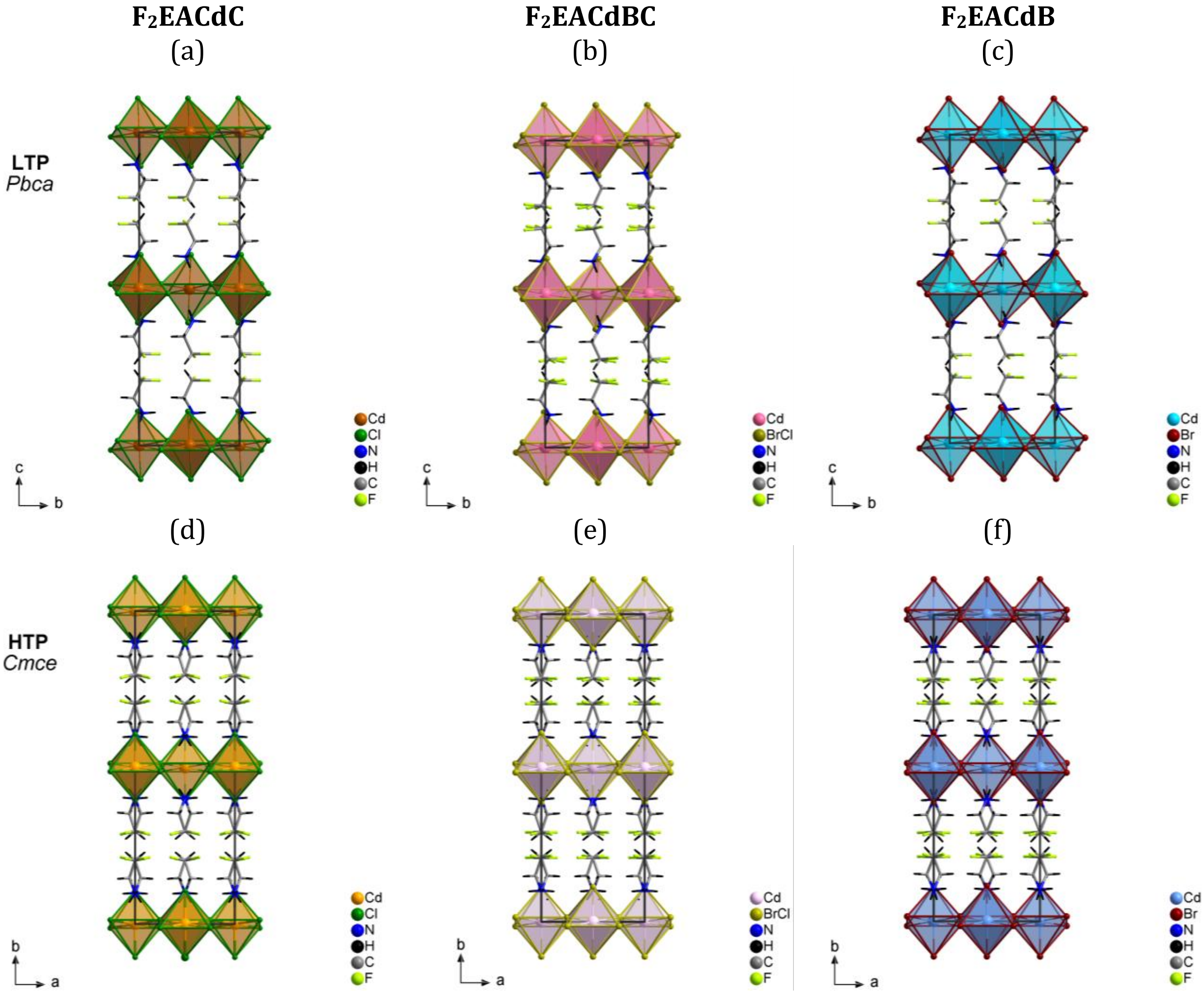


**Figure S6**. Corresponding views in the LT phase (a-c) and the HT phase (d-f) along $a_{LT} \rightarrow c_{\mathrm{HT}}$.

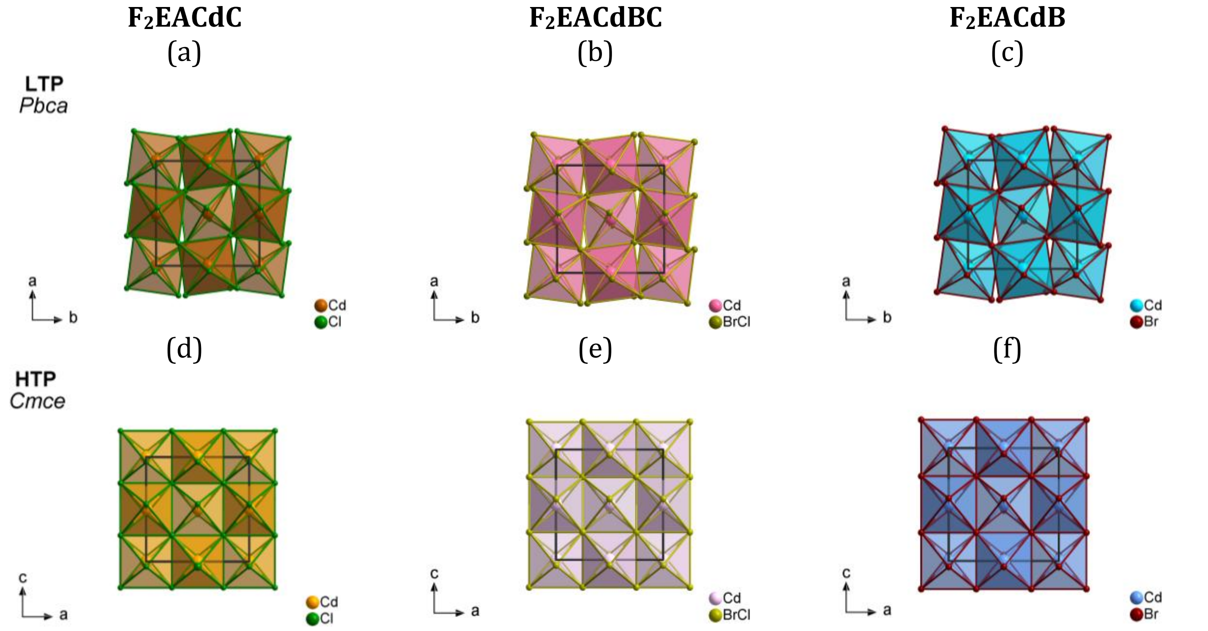


**Figure S7.** Corresponding views in the LT phase (a-c) and the HT phase (d-f) along $c_{LT} \rightarrow b_{\mathrm{HT}}$. The organic part is omitted for clarity.

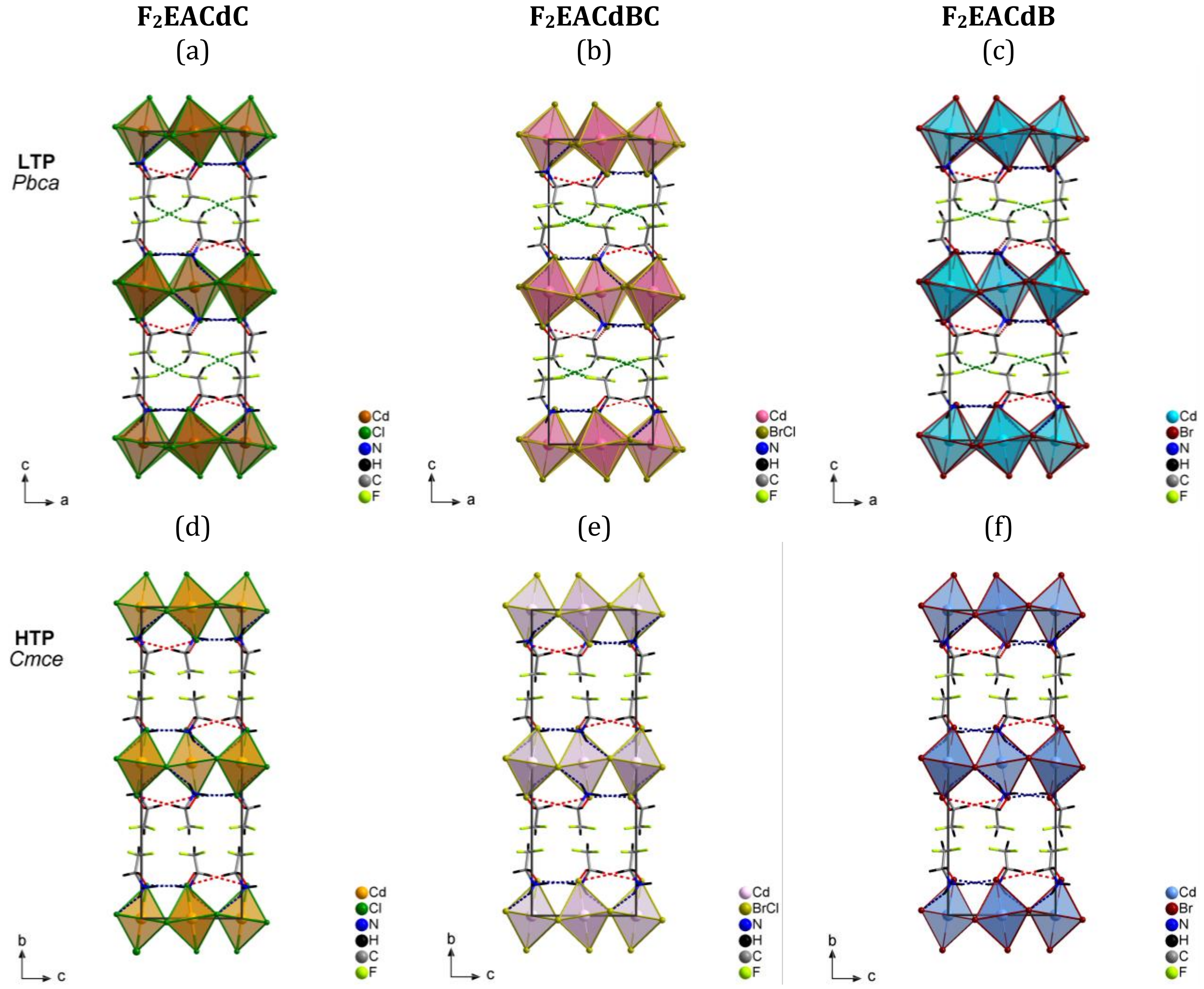


**Figure S8.** Corresponding views in the LT phase (a-c) and the HT phase (d-f) along $b_{\mathrm{LT}} \rightarrow a_{\mathrm{HT}}$, showing N–H···Br/Cl, C–H···Br/Cl, and C–H···F interactions, represented by blue, red and green dashed lines, respectively.

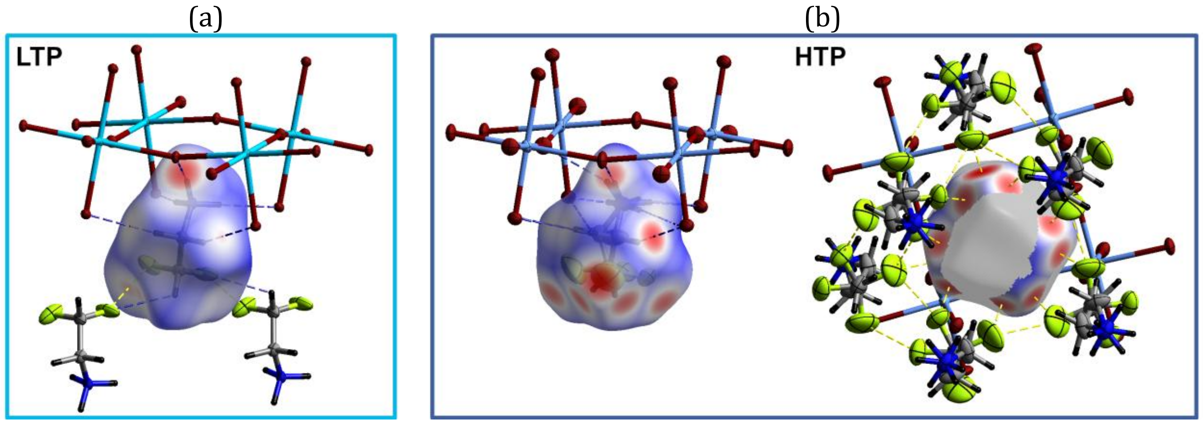


**Figure S9**. Comparison of Hirshfeld surfaces of $F_2EA^+$ in **$F_2$EACdB** in the LT and HT phase without close-contact filtering (a, b) and with close contact decomposition mapped for F···F for HT phase (b). The Hirshfeld surface is mapped with $d_{\mathrm{norm}}$, highlighting both donor and acceptor ability. Dashed lines indicate hydrogen bonds: N–H···Br/Cl, C–H···Br/Cl (blue), and C–H···F (green).

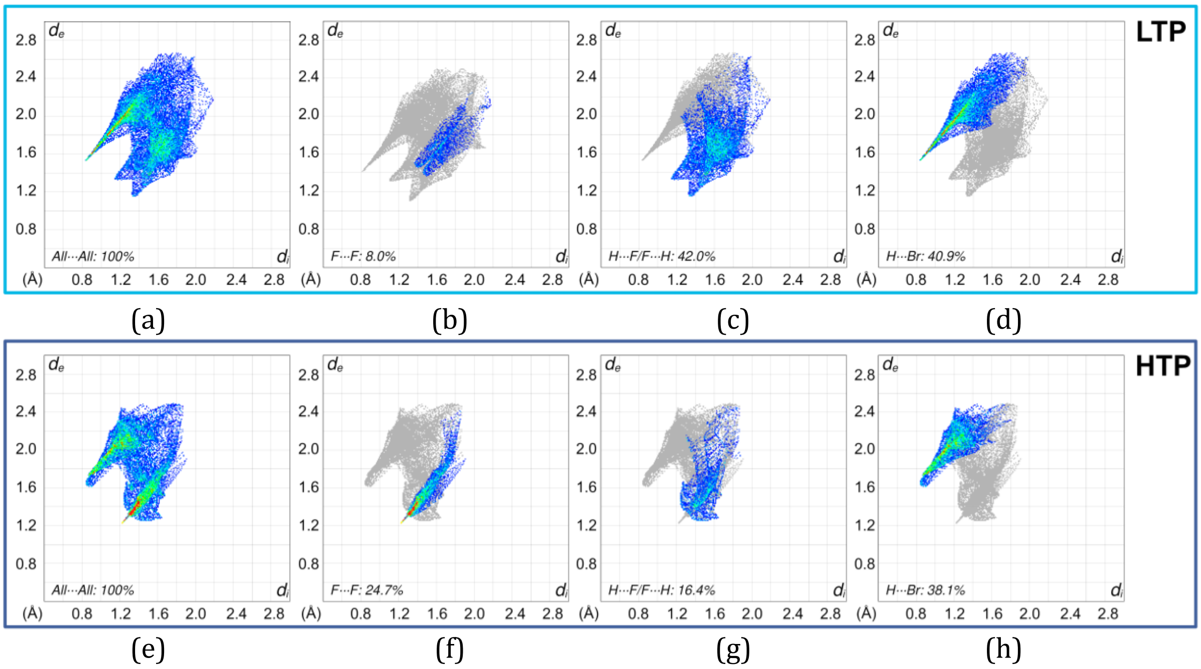


**Figure S10**. Fingerprint plots of the Hirshfeld surfaces without close contacts (a) and (e), and with close contacts decomposition mapped for: F···F (b, f), H···F (c, g), and H···Br (d, h) in **$F_2$EACdB** for the LT (a-d) and HT (e-h) phase.

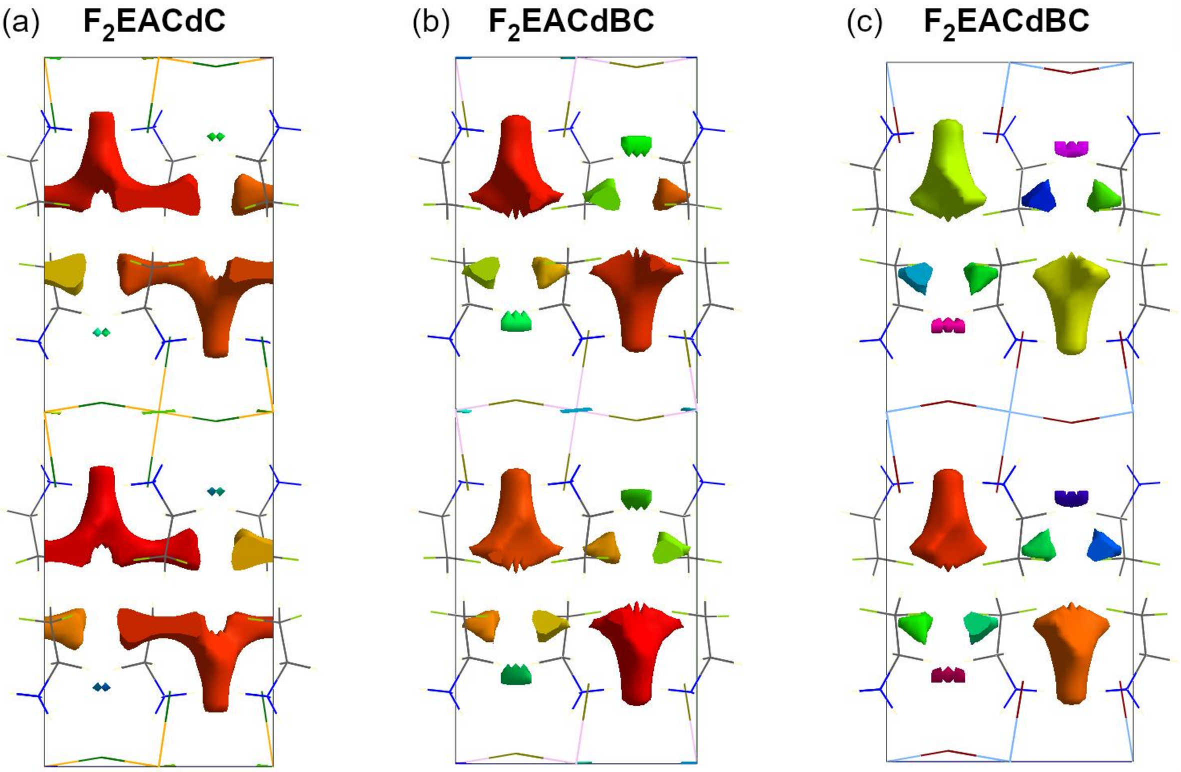


**Figure S11**. The representation of void domains in the HT phase calculated in CrystalExplorer with an isovalue of 0.002 e au$^{-3}$.

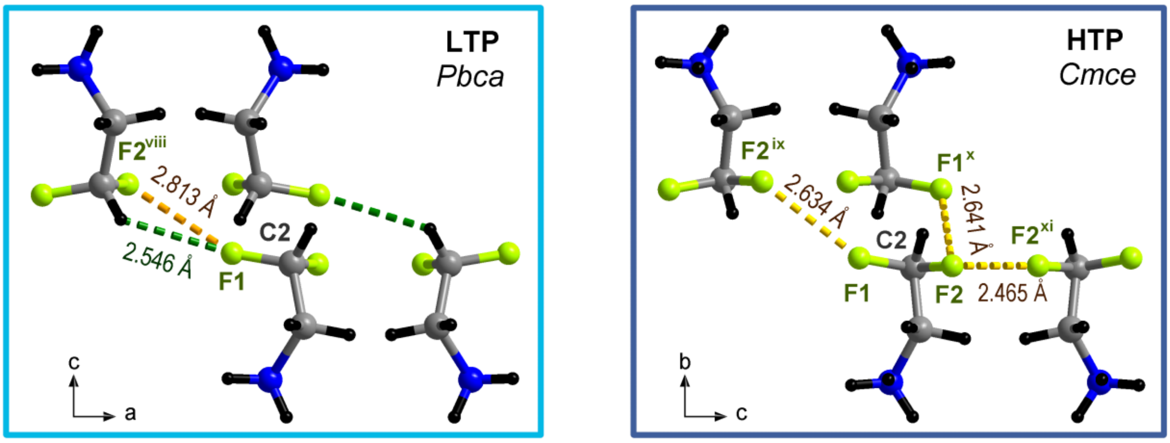


**Figure S12**. Fragment of the $F_2$**EACdB** structure showing changes in C–H···F interactions and the F···F distances between the LT and HT phases. The disorder in the HT phase is not visible from this viewing direction due to the mirror-plane orientation.

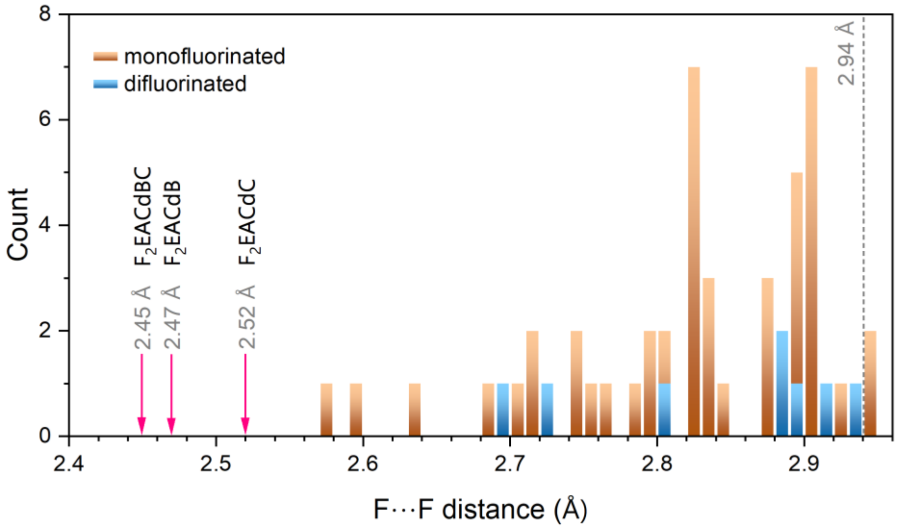


**Figure S13**. Diagram showing search results in CSD for structures with intermolecular F···F distances below the sum of van der Waals radii of 2.94 Å.

## 4. NCI calculations

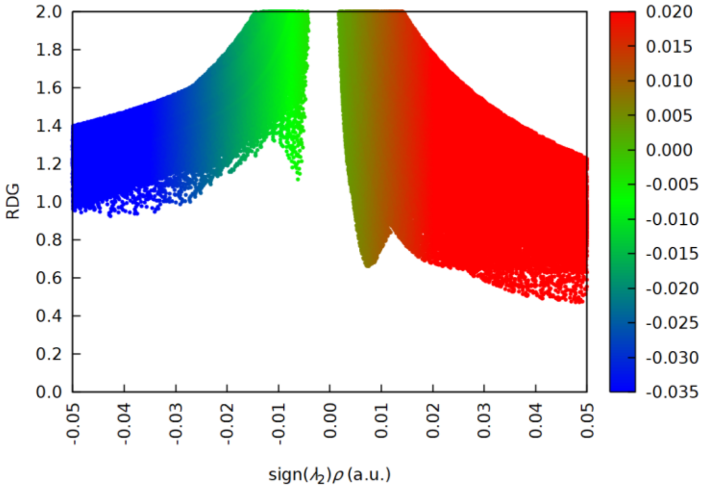


**(a)**

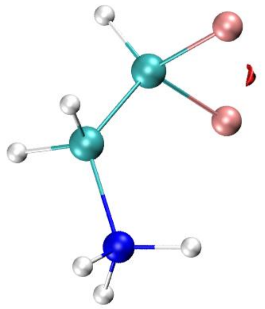

**(b)**

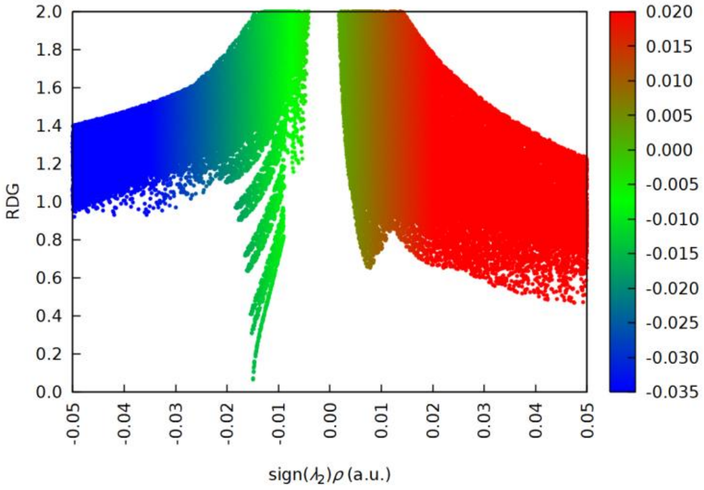


**(c)**

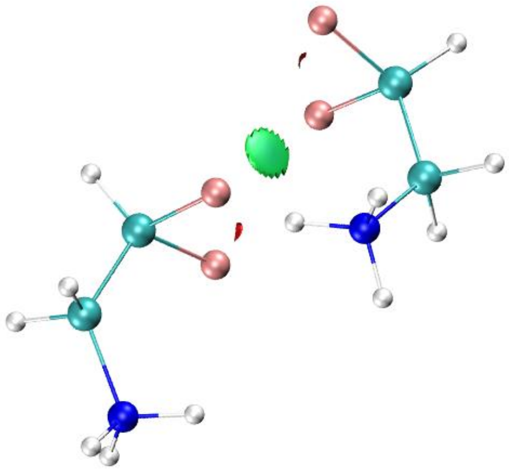

**(d)**

**Figure S14**. The RDG *vs* sign($\lambda_2$)$\rho$ plots (a, c) and corresponding 0.5 RDG isosurfaces (b, d) calculated for one $F_2EA^+$ cation (a, b) and two cations (c, d) for **$F_2$EACdB** in the HTP phase; colors: blue, attractive interactions ($\lambda_2 < 0$); green, van der Waals interactions ($\lambda_2 \approx 0$); red, repulsive interactions ($\lambda_2 > 0$);atom colors: blue, N; turquoise, C; pink, F; white, H.

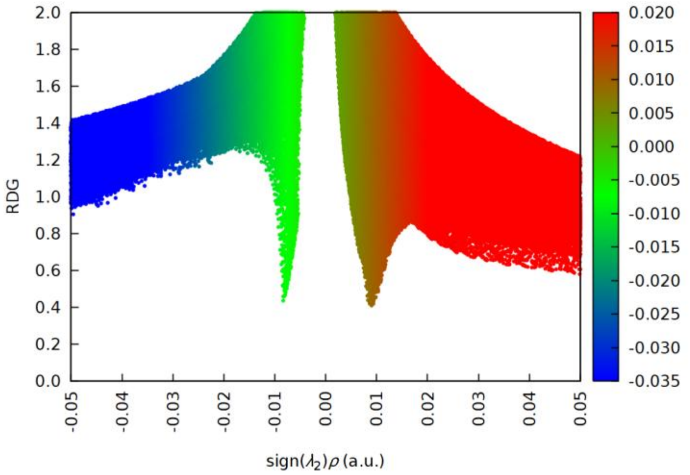


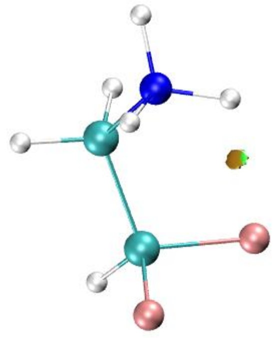

**(a)** **(b)**

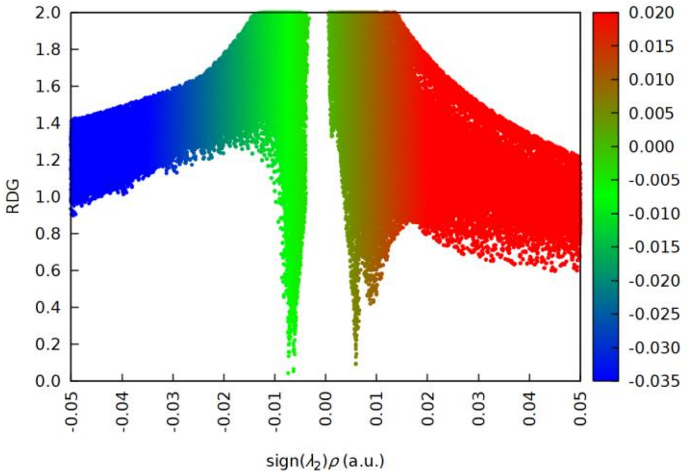


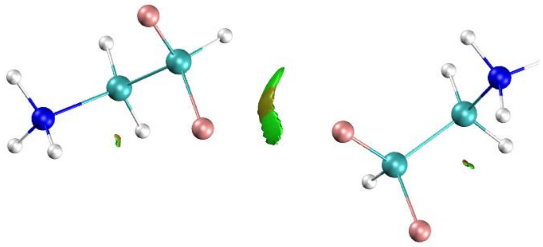

**(c)** **(d)**

**Figure S15**. The RDG *vs* sign($\lambda_2$)$\rho$ plots (a, c) and corresponding 0.5 RDG isosurfaces (b, d) calculated for one $F_2EA^+$ cation (a, b) and two cations (c, d) for **$F_2$EACdB** in the LTP phase; colors: blue, attractive interactions ($\lambda_2 < 0$); green, van der Waals interactions ($\lambda_2 \approx 0$); red, repulsive interactions ($\lambda_2 > 0$); atom colors: blue, N; turquoise, C; pink, F; white, H.

## 5. DFT calculations

**Table S10**. Selected bond lengths (Å) and angles (°) of $F_2EA$ and $F_2EA^+$ cation.

| FA molecule | | $FA^+$ cation | |
|---|---|---|---|
| **Bondlength/angle** | **Calculated** | **Bondlength/angle** | **Calculated** |
| N1–C4 | 1.456 | N1–C5 | 1.512 |
| N1–H2 | 1.014 | N1–H2 | 1.021 |
| N1–H3 | 1.014 | N1–H3 | 1.025 |
| | | N1–H4 | 1.025 |
| C4–H5 | 1.092 | C5–H6 | 1.086 |
| C4–H6 | 1.092 | C5–H7 | 1.086 |
| C4–C7 | 1.517 | C5–C8 | 1.519 |
| C7–H8 | 1.092 | C8–H9 | 1.088 |
| C7–F9 | 1.372 | C8–F10 | 1.363 |
| C7–F10 | 1.372 | C8–F11 | 1.362 |
| N1–C4–C7 | 114.81 | N1–C5–C8 | 106.64 |
| H2–N1–C4 | 109.45 | H2–N1–C5 | 112.23 |
| H3–N1–C4 | 109.45 | H3–N1–C5 | 109.94 |
| | | H4–N1–C5 | 109.90 |
| H2–N1–H3 | 106.23 | H2–N1–H3 | 108.65 |
| | | H2–N1–H4 | 108.66 |
| N1–C4–H5 | 109.46 | N1–C5–H6 | 108.33 |
| N1–C4–H6 | 109.46 | N1–C5–H7 | 108.33 |
| C4–C7–H8 | 114.29 | C5–C8–H9 | 113.84 |
| C4–C7–F9 | 109.88 | C5–C8–F10 | 107.74 |
| C4–C7–F10 | 109.89 | C5–C8–F11 | 107.75 |
| F9–C7–F10 | 106.80 | F10–C8–F11 | 117.51 |
| F9–C7–H8 | 107.84 | F10–C8–H9 | 109.88 |
| N1–C4–C7–H8 | 179.94 | H9–C8–C5–N1 | 179.83 |
| N1–C4–C7–F9 | 58.57 | F10–C8–C5–N1 | 57.72 |
| N1–C4–C7–F10 | -58.66 | F11–C8–C5–N1 | -58.01 |

**Table S11**. Calculated harmonic ($\nu_{HA}$) and anharmonic ($\nu_{AA}$) wavenumbers for $F_2EA$ molecule and $F_2EA^+$ cation as well as potential energy distribution (PED, %) of the respective predominant modes; contributions higher than 30% PED are bolded.

| **$F_2EA$ molecule** | | | | | **$F_2EA^+$ cation** | | | | |
|---|---|---|---|---|---|---|---|---|---|
| **#** | **$\nu_{HA}$** | **$\nu_{HA}$*** | **$\nu_{AA}$** | **PED (%)** | **#** | **$\nu_{HA}$** | **$\nu_{HA}$*** | **$\nu_{AA}$** | **PED (%)** |
| 1 | 3567 | 3424 | 3397 | **$\nu_{as}NH_2$ (100)** | 1 | 3493 | 3353 | 3328 | **$\nu_{as}NH_3^+$ (100)** |
| 2 | 3497 | 3357 | 3351 | **$\nu_sNH_2$ (100)** | 2 | 3454 | 3316 | 3276 | **$\nu_{as}NH_3^+$ (100)** |
| | | | | | 3 | 3395 | 3259 | 3263 | **$\nu_sNH_3^+$ (100)** |
| 3 | 3084 | 2961 | 2941 | **$\nu_{as}CH_2$ (100)** | 4 | 3173 | 3046 | 3038 | **$\nu_{as}CH_2$ (100)** |
| 4 | 3069 | 2946 | 2952 | **$\nu CH$ (90)** + $\nu_sCH_2$ (10) | 5 | 3132 | 3007 | 3014 | **$\nu CH$ (98)** |
| 5 | 3044 | 2922 | 2929 | **$\nu_sCH_2$ (90)** + $\nu CH$ (10) | 6 | 3109 | 2985 | 3002 | **$\nu_sCH_2$ (98)** |
| 6 | 1682 | 1648 | 1634 | **$\delta NH_2$ (98)** | 7 | 1680 | 1647 | 1617 | **$\delta_{as}NH_3^+$ (99)** |
| | | | | | 8 | 1659 | 1626 | 1610 | **$\delta_{as}NH_3^+$ (99)** |
| | | | | | 9 | 1525 | 1495 | 1479 | **$\delta_sNH_3^+$ (99)** |
| 7 | 1475 | 1446 | 1437 | **$\delta CH_2$ (100)** | 10 | 1492 | 1462 | 1455 | **$\delta CH_2$ (100)** |
| 8 | 1413 | 1384 | 1385 | **$\tau CH_2$ (47) + $\rho NH_2$ (31)** + $\gamma CH$ (23) | 11 | 1421 | 1392 | 1386 | **$\omega CH_2$ (71)** + $\nu CC$ (20) |
| 9 | 1399 | 1371 | 1366 | **$\delta CH$ (57) + $\omega CH_2$ (40)** | 12 | 1389 | 1361 | 1357 | **$\delta CH$ (79)** + $\omega CH_2$ (15) |
| 10 | 1381 | 1353 | 1349 | **$\omega CH_2$ (52)** + $\delta CH$ (25) + $\nu CC$ (22) | 13 | 1380 | 1353 | 1349 | **$\gamma CH$ (71)** + $\tau CH_2$ (22) |
| 11 | 1374 | 1347 | 1336 | **$\gamma CH$ (62)** + $\rho NH_2$ (16) + $\gamma CF$ (16) + $\rho CH_2$ (11) | 14 | 1333 | 1306 | 1298 | **$\tau CH_2$ (69)** + $\rho NH_3^+$(21) |
| 12 | 1208 | 1184 | 1181 | **$\tau CH_2$ (51)** + $\rho NH_2$ (28) + $\nu CF$ (170 + $\gamma CH$ (10) | 15 | 1153 | 1130 | 1125 | **$\nu CF$ (41) + $\rho CH_2$ (36)** + $\rho NH_3^+$ (18) + $\gamma CH$ (10) |
| 13 | 1152 | 1129 | 1122 | **$\nu CN$ (41) + $\nu CF$ (40)** + $\nu CC$ (19) | 16 | 1145 | 1122 | 1119 | **$\nu CF$ (73)** + $\rho NH_3^+$ (11) + $\delta CH_2$ (13) |
| 14 | 1118 | 1095 | 1088 | **$\nu CF$ (48)** + $\nu CN$ (26) + $\omega NH_2$ (16) + $\delta CH_2$ (11) | 17 | 1092 | 1070 | 1058 | **$\rho NH_3^+$ (63)** + $\nu CF$ (24) + $\delta CH_2$ (13) |
| 15 | 1045 | 1024 | 1023 | **$\nu CF$ (70)** + $\tau CH_2$ (20) + $\rho NH_2$ (10) | 18 | 1030 | 1009 | 1008 | **$\nu CF$ (56)** + $\rho NH_3^+$ (26) + $\tau CH_2$ (21) |
| 16 | 931 | 912 | 881 | **$\omega NH_2$ (62)** + $\nu CC$ (17) + $\nu CN$ (16) | 19 | 983 | 964 | 956 | **$\nu CN$ (41)** + $\nu CC$ (25) + $\nu CF$ (16) + $\omega CH_2$ (11) |
| 17 | 862 | 845 | 853 | **$\rho CH_2$ (59)** + $\nu CF$ (27) + $\rho NH_2$ (13) | 20 | 859 | 842 | 846 | **$\rho CH_2$ (53)** + $\rho NH_3^+$ (28) + $\nu CF$ (19) |
| 18 | 859 | 842 | 837 | **$\nu CC$ (42) + $\nu CN$ (33)** + $\delta CF$ (14) + $\delta CH$ (11) | 21 | 850 | 833 | 824 | **$\nu CN$ (57) + $\nu CC$ (43)** |
| 19 | 727 | 713 | 718 | **$\gamma CF$ (72)** + $\delta CH_2$ (22) + $\nu CC$ (10) + $\omega NH_2$ (10) | 22 | 698 | 684 | 690 | **$\gamma CF$ (59)** + $\delta CH_2$ (17) + $\rho NH_3^+$ (15) + $\nu CC$ (9) |
| 20 | 501 | 491 | 496 | **$\gamma CF$ (96)** | 23 | 501 | 491 | 496 | **$\gamma CF$ (95)** |
| 21 | 399 | 391 | 392 | **$\delta CF$ (60)** + $\tau NH_2$ (27) + $\rho CH_2$ (11) | 24 | 396 | 388 | 393 | **$\delta CF$ (80)** + $\rho CH_2$ (11) |
| 22 | 285 | 280 | 266 | **$\tau NH_2$ (74)** + $\delta CF$ (24) | 25 | 222 | 218 | 225 | **$\tau NH_3^+$ (93)** |
| 23 | 255 | 250 | 257 | **$\delta CCN$ (66) + $\delta CF$ (34)** | 26 | 259 | 253 | 269 | **$\delta CCN$ (67)** + $\delta CF$ (30) |
| 24 | 126 | 124 | 122 | **$\tau CF$ (98)** | 27 | 122 | 120 | 121 | **$\tau CF$ (98)** |

* scaling factor = 0.98 (2499-0 $cm^{-1}$) + 0.96 (3600-2500 $cm^{-1}$); in-plane vibrations: $\nu$, stretching; $\delta$, scissoring; $\rho$, rocking; out-of-plane vibrations: $\omega$, wagging; $\tau$, twisting; $\gamma$, out-of-plane bending.

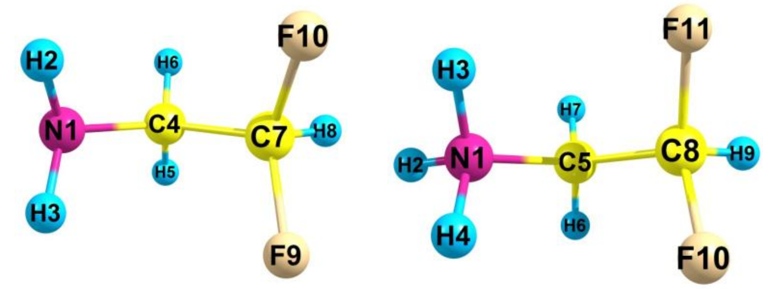


(a) (b)

**Figure S16**. The numbering of atoms in $F_2EA$ molecule (a) and $F_2EA^+$ cation (b) used for the DFT calculations.

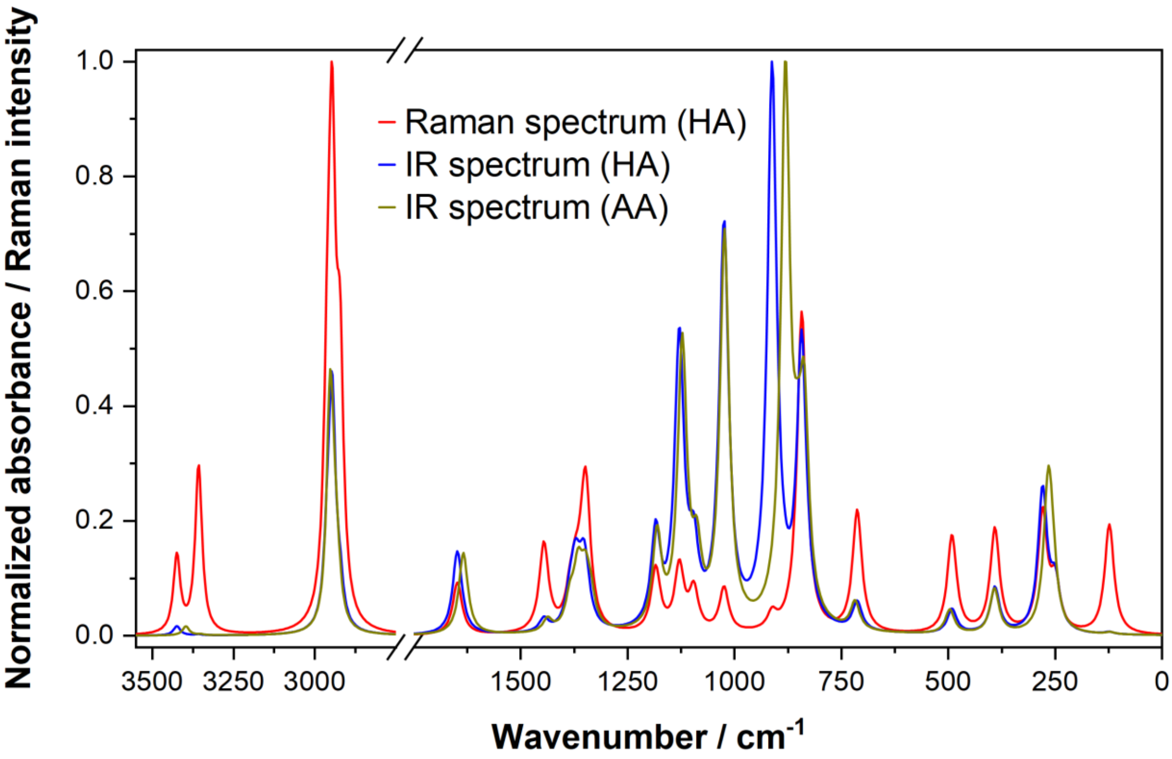


**Figure S17**. A comparison of calculated IR and Raman spectra of $F_2EA$ in harmonic (HA) and anharmonic approximation (AA); the HH spectra were scaled by 0.98 (2499-0 $cm^{-1}$) and 0.96 (3600-2500 $cm^{-1}$).

## 6. Vibrational studies

*Factor group analysis and selection rules*

The 24 vibrational degrees of freedom of the $F_2EA$ molecule can be subdivided into 9 stretching, 14 bending, and 1 torsional mode. The stretching modes can be roughly described as 2 × νCF, 2 × $\nu NH_2$, 2 × $\nu CH_2$, νCC, νCH and νCN. The bending modes can be described as: scissoring, $\delta NH_2$ and $\delta CH_2$; rocking, $\rho CH_2$ and $\rho NH_2$; wagging, $\omega CH_2$ and $\omega NH_2$, twisting, $\tau CH_2$ and $\tau NH_2$, in-plane bending, δCF and δCH; out-of-plane bending, 2 × γCF and γCH; and skeleton bending, δCCN. The remaining torsional vibration is τCF and. The presence of one additional proton in the $F_2EA^+$ cation leads to the appearance of additional $\nu NH_3$ and two $\delta NH_3$ vibrations.

According to the factor group analysis, the total number of optical phonon modes for the orthorhombic *Pbca* LT phase is equal to 321 ($39A_g + 42A_u + 39B_{1g} + 41B_{1u} + 39B_{2g} + 41B_{2u} + 39B_{3g} + 41B_{3u}$). They can be further subdivided into 57 modes originating from the inorganic lattice ($6A_g + 9A_u + 6B_{1g} + 8B_{1u} + 6B_{2g} + 8B_{2u} + 6B_{3g} + 8B_{3u}$) and 264 modes associated with organic cations ($33A_g + 33A_u + 33B_{1g} + 33B_{1u} + 33B_{2g} + 33B_{2u} + 33B_{3g} + 33B_{3u}$). According to this, 156 modes are Raman-active (24 related to inorganic layers and 132 to organic cations) and 123 are IR-active (24 related to inorganic layers and 99 to organic cations).

Since the HT phase is disordered, only the total number of modes can be calculated, and the representation can be given only for the ordered inorganic sublattice. Since the primitive cell of the *Cmce* structure is twice lower, the number of optical phonons is 159, subdivided into 27 ($3A_g + 3A_u + 3B_{1g} + 5B_{1u} + 2B_{2g} + 4B_{2u} + 4B_{3g} + 3B_{3u}$) modes related to the inorganic slabs and 132 to the organic cations.

Summarizing, the *Pbca* → *Cmce* PT is expected to be visible as a decrease in the number of vibrational bands by half for the inorganic part (from 24 Raman and IR to 12), as well as by about half for organic cations.

**Table S12**. A list of observed room-temperature IR and Raman wavenumbers for **$F_2$EACdC**, **$F_2$EACdBC**, and **$F_2$EACdB** and proposed assignment; bands with intensity between medium and very strong are bold.

| **$F_2$EACdC** IR | Raman | **$F_2$EACdBC** IR | Raman | **$F_2$EACdB** IR | Raman | **Assignment** |
|---|---|---|---|---|---|---|
| 3169$_{sh}$, **3097$_{s}$** | 3101$_{sh}$ | 3169$_{sh}$, **3089$_{s}$** | | 3165$_{sh}$, **3091$_{s}$** | 3100$_{sh}$ | $\nu_{as}NH_3^+$ |
| **3028$_{s}$** | **3029$_{m}$** | **3025$_{s}$** | **3026$_{m}$** | **3024$_{s}$** | **3027$_{m}$** | $\nu_{s}NH_3^+$ |
| 3013$_{sh}$ | **3013$_{m}$** | 3011$_{sh}$ | **3011$_{m}$** | 3008$_{sh}$ | **3007$_{m}$** | $\nu_{as}CH_2$ |
| 2979$_{sh}$ | **2980$_{vs}$** | 2976$_{sh}$ | **2976$_{s}$** | 2976$_{sh}$ | **2974$_{m}$** | $\nu CH$ |
| **2935$_{s}$** | 2943$_{sh}$ | **2931$_{s}$** | 2938$_{w}$ | **2930$_{s}$** | 2934$_{w}$ | $\nu_{as}CH_2$ |
| 2896$_{sh}$ | 2891$_{w}$ | 2891$_{sh}$ | 2883$_{w}$ | 2888$_{sh}$ | 2882$_{w}$ | $\nu_{s}CH_2$ |
| 2804$_{vw}$ | 2819$_{w}$, 2784$_{w}$ | 2799$_{vw}$ | 2812$_{w}$, 2777$_{w}$ | 2796$_{vw}$ | 2812$_{w}$, 2779$_{w}$ | o/cb |
| **1588$_{m}$**, 1574$_{sh}$ | 1582$_{w}$ | **1586$_{m}$**, 1575$_{sh}$ | 1573$_{w}$ | **1589$_{m}$**, 1577$_{sh}$ | 1573$_{w}$ | $\delta_{as}NH_3$ |
| **1493$_{vs}$** | 1493$_{w}$ | **1490$_{vs}$** | 1487$_{w}$ | **1488$_{vs}$** | 1484$_{w}$ | $\delta_{s}NH_3$ |
| 1446$_{w}$ | 1456$_{w}$, 1449$_{w}$ | 1445$_{w}$ | 1447$_{w}$ | 1445$_{w}$ | 1447$_{w}$ | $\delta CH_2+\omega CH_2$ |
| 1419$_{vw}$ | 1416$_{w}$ | 1418$_{vw}$ | 1415$_{w}$ | 1417$_{vw}$ | 1414$_{w}$, 1402$_{sh}$ | $\omega CH_2$ |
| 1385$_{w}$, 1365$_{w}$ | 1379$_{w}$, 1369$_{w}$ | 1383$_{w}$, 1363$_{w}$ | 1378$_{w}$, 1369$_{w}$ | 1381$_{w}$, 1361$_{w}$ | 1384$_{w}$, 1378$_{w}$, 1369$_{w}$ | $\delta CH+\gamma CH$ |
| **1319$_{m}$** | 1316$_{vw}$ | 1315$_{w}$ | 1314$_{vw}$ | 1314$_{w}$ | 1314$_{vw}$ | $\tau CH_2$ |
| **1142$_{m}$** | **1136$_{s}$** | **1139$_{m}$** | **1135$_{s}$** | 1138$_{w}$ | **1134$_{s}$** | $\nu CF+\rho CH_2$ |
| **1111$_{m}$** | **1105$_{s}$** | 1105$_{w}$ | **1103$_{s}$** | 1102$_{w}$ | **1102$_{s}$** | $\nu CF$ |
| 1053$_{w}$ | | 1053$_{w}$ | | 1053$_{w}$ | | $\rho NH_3^+ + \nu CF$ |
| 1025$_{sh}$, **1017$_{vs}$**, 1011$_{sh}$ | **1027$_{s}$**, 1012$_{sh}$ | 1024$_{sh}$, **1016$_{vs}$**, 1010$_{sh}$ | **1020$_{m}$**, 1011$_{sh}$ | 1023$_{sh}$, **1016$_{vs}$**, 1010$_{sh}$ | **1019$_{m}$**, 1010$_{sh}$ | $\nu CF+\rho NH_3^+$ |
| 953$_{vw}$ | | 951$_{vw}$ | | 951$_{vw}$ | | $\nu CN+\nu CF$ |
| 864$_{w}$, 858$_{sh}$ | **868$_{m}$**, **858$_{s}$** | 864$_{w}$, 857$_{sh}$ | 865$_{sh}$, **857$_{s}$** | 864$_{w}$, 856$_{sh}$ | 862$_{sh}$, **855$_{s}$** | $\rho CH_2+\nu CN+\nu CC$ |
| 709$_{w}$, 510$_{w}$ | **707$_{m}$**, 574$_{vw}$, 512$_{w}$ | 709$_{w}$, 510$_{w}$ | **706$_{m}$**, 512$_{w}$ | 709$_{w}$ | 705$_{w}$, 512$_{w}$ | $\gamma CF$ |
| | 440$_{vw}$, 393$_{w}$ | | 391$_{w}$ | | 391$_{w}$ | $\delta CF$ |
| | 264$_{w}$, 241$_{w}$ | | 261$_{w}$ | | 261$_{w}$ | $\delta CH_2$ |
| | **207$_{s}$** | | 211$_{sh}$ | | | $\nu CdCl+\tau NH_3^+$ |
| | **184$_{m}$** | | | | | $\nu CdCl$ |
| | | | | | 164$_{sh}$ | $\nu CdBr$ |
| | **142$_{m}$** | | **142$_{vs}$** | | | $\nu CdCl$+lm |
| | | | 121$_{sh}$ | | **134$_{vs}$** | $\nu CdBr+\gamma CF$+lm |
| | **96$_{vs}$**, **78$_{vs}$**, **60$_{vs}$** | | **93$_{vs}$**, **57$_{m}$** | | **89$_{vs}$**, **58$_{vs}$** | $\delta CdCl+\delta CdBr$+lm |

in-plane vibrations: ν, stretching; δ, scissoring or in-plane bending; ρ, rocking; out-of-plane vibrations: ω, wagging; τ, twisting; γ, out-of-plane bending; symbols: o/cb, overtone or combinational band; lm, lattice mode

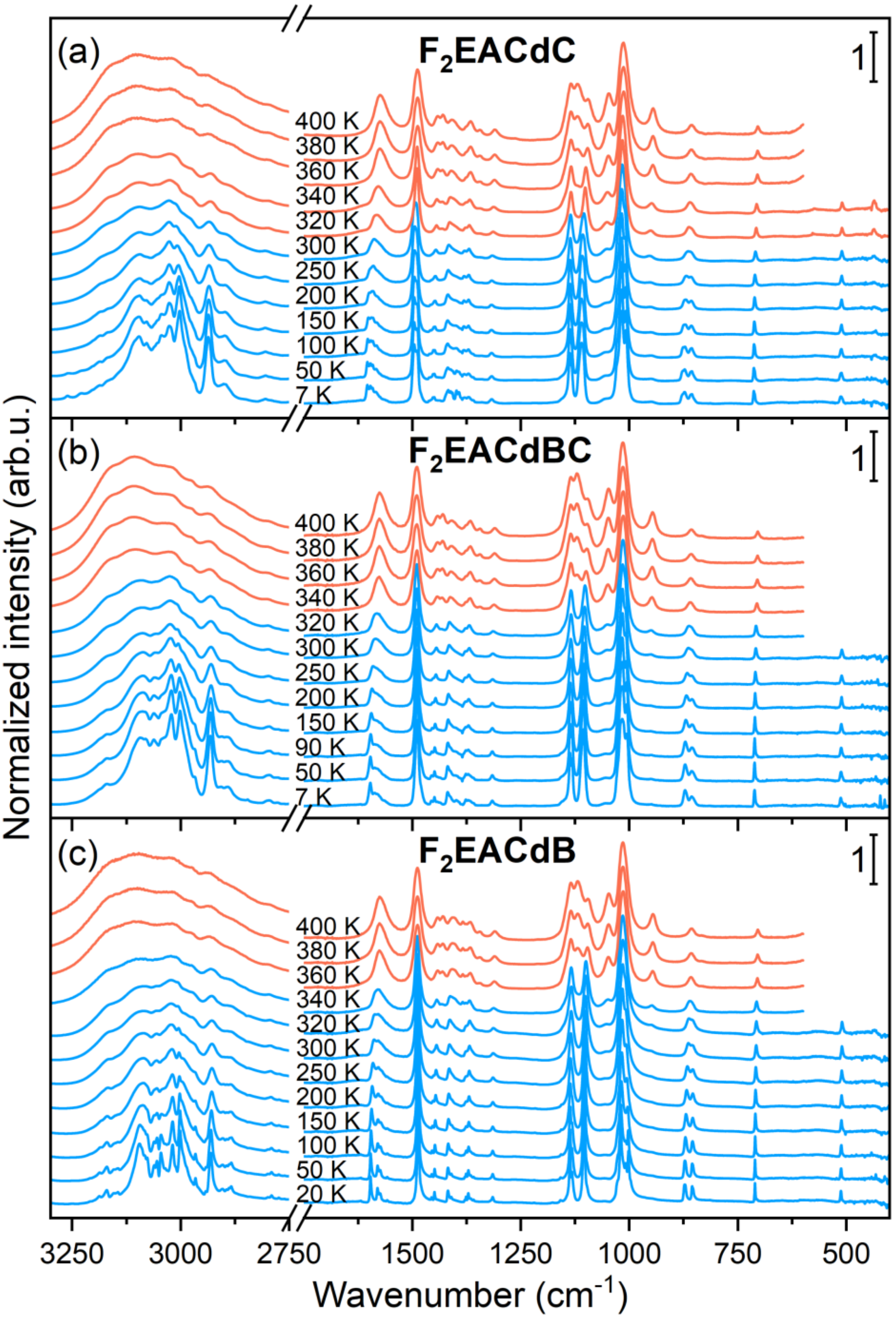


**Figure S18**. A comparison of thermal evolution of IR spectra measured for (a) $F_2$EACdC, (b) $F_2$EACdBC, and (c) $F_2$EACdB.

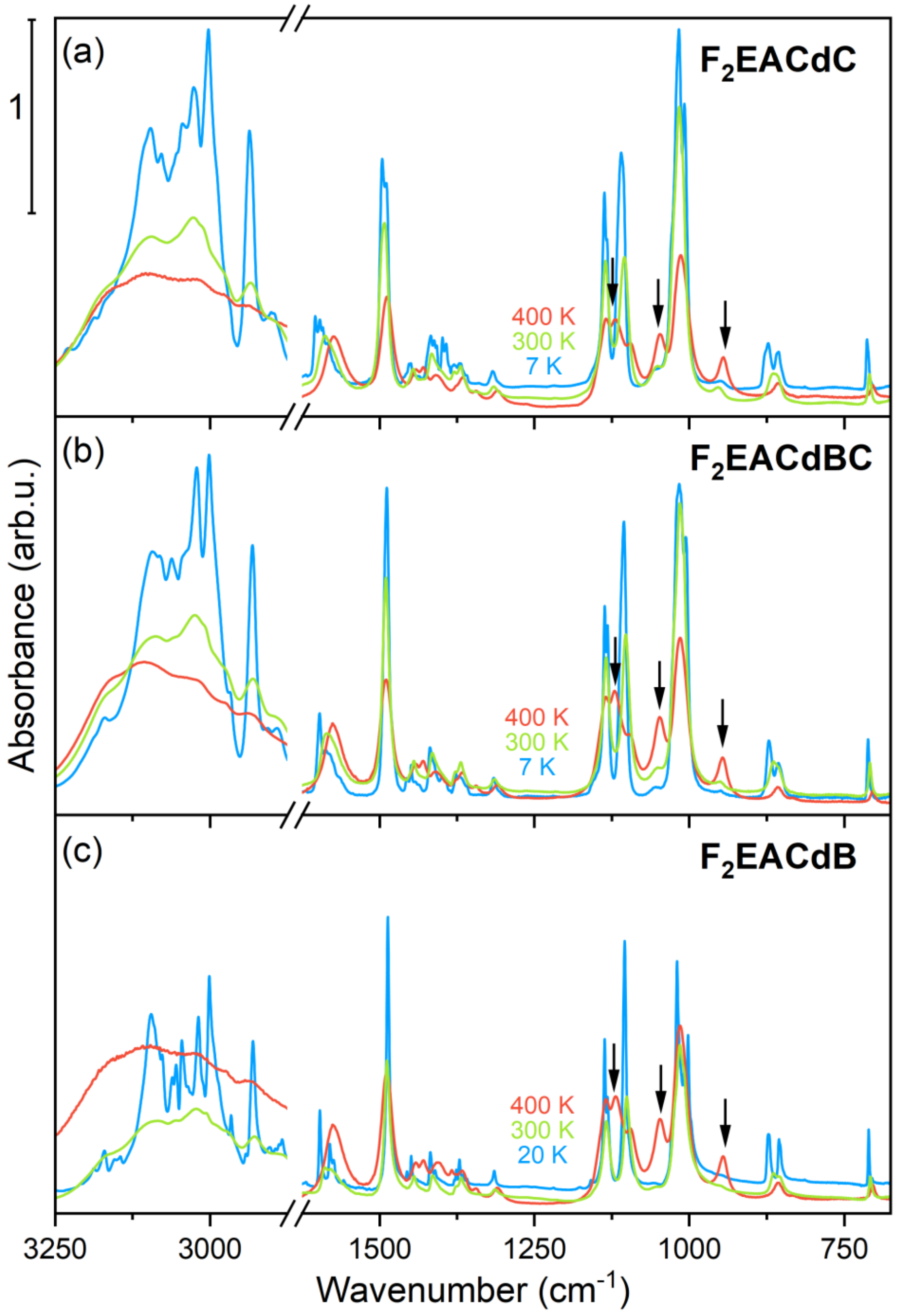


**Figure S19**. A comparison of IR spectra measured for (a) $F_2EACdC$, (b) $F_2EACdBC$, and (c) $F_2EACdB$ at 7/20, 300, and 400 K.

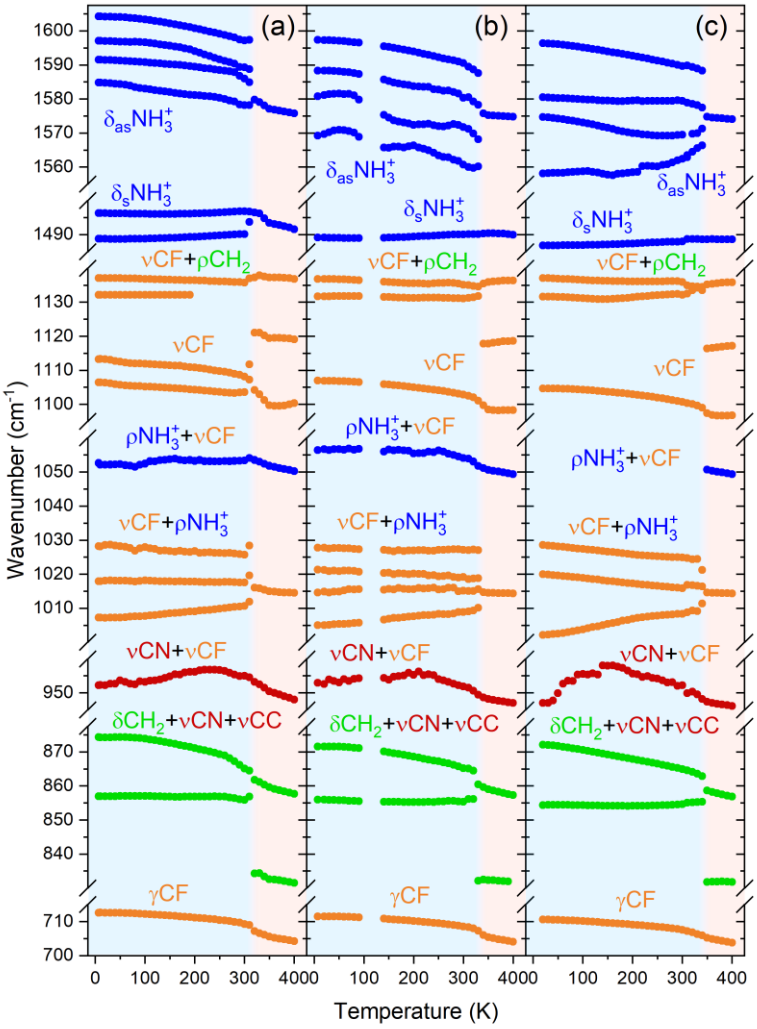


**Figure S20**. Temperature dependence of band positions for (a) $F_2EACdC$, (b) $F_2EACdBC$, and (c) $F_2EACdB$.

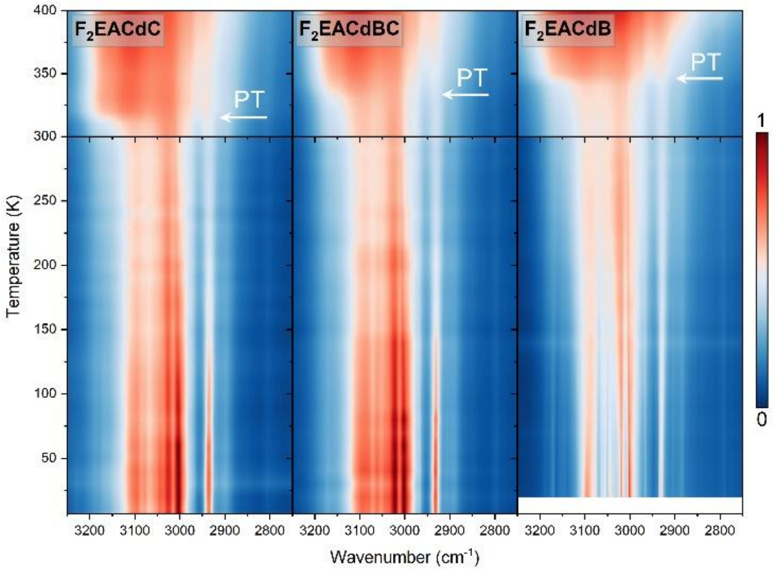


**Figure S21**. A color map of thermal evolution for bands corresponding to the ν$NH_3^+$, ν$NH_3$, and νCH vibrations for three studied compounds.

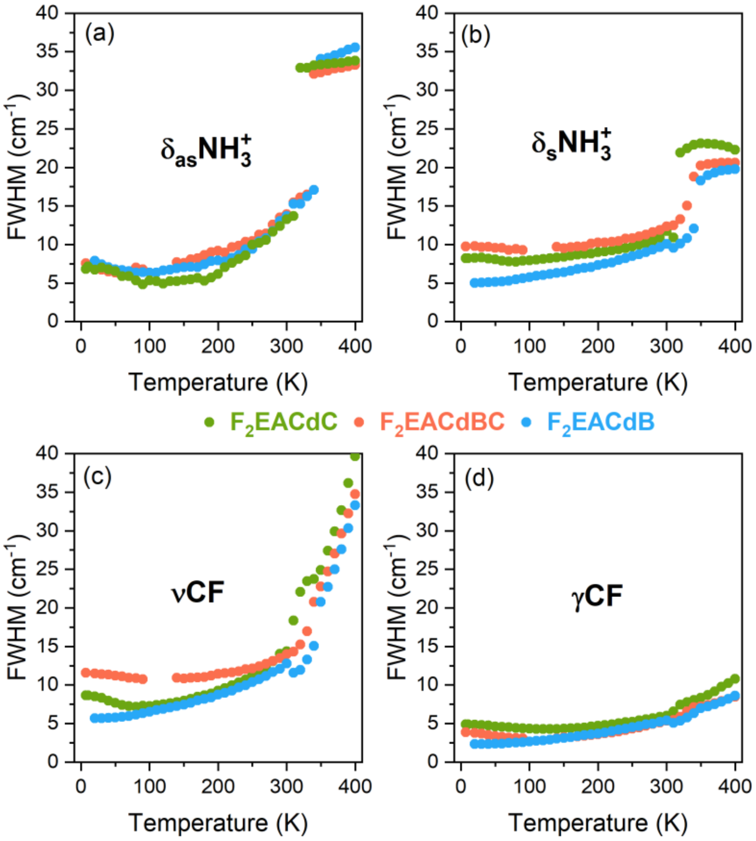


**Figure S22**. Changes of FWHM (full width at half maximum) for IR bands corresponding to (a) $\delta_{as}NH_3^+$ (1586-1589 cm$^{-1}$), (b) $\delta_sNH_3^+$ (1488-1493 cm$^{-1}$), (c) νCF (1134-1142 cm$^{-1}$), and (d) γCF (709 cm$^{-1}$) vibrations.

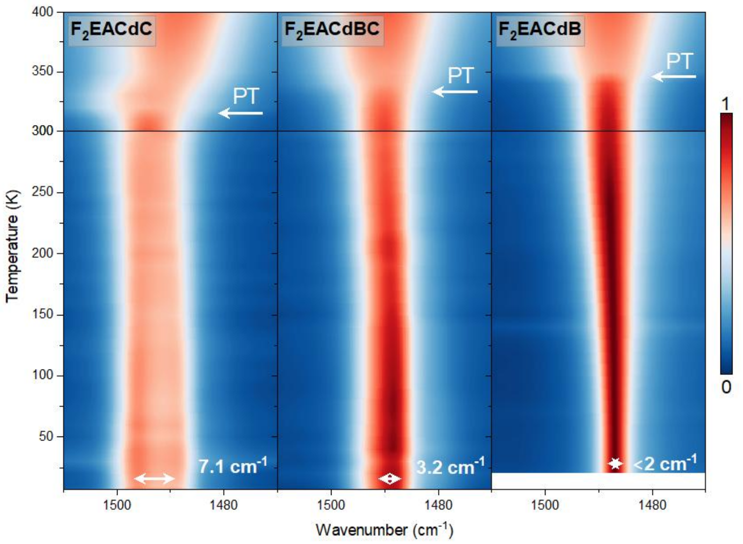


**Figure S23**. A color map of thermal evolution for bands corresponding to the $\delta_sNH_3^+$ vibrations for three studied compounds; horizontal arrows show the magnitude of Davydov splitting.

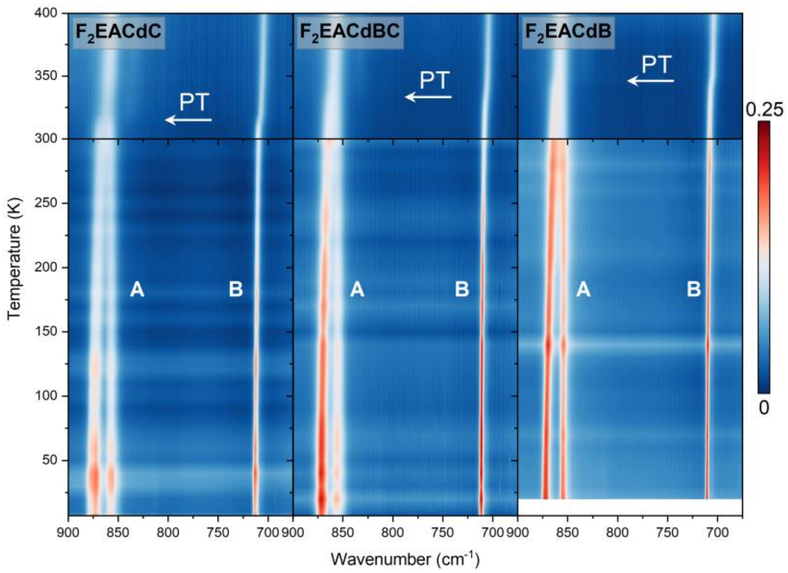


**Figure S24**. A color map of thermal evolution for bands corresponding to the $\rho CH_2+\nu CN+\nu CC$ (A) and $\gamma CF$ (B) vibrations for three studied compounds.

## 7. Optical properties

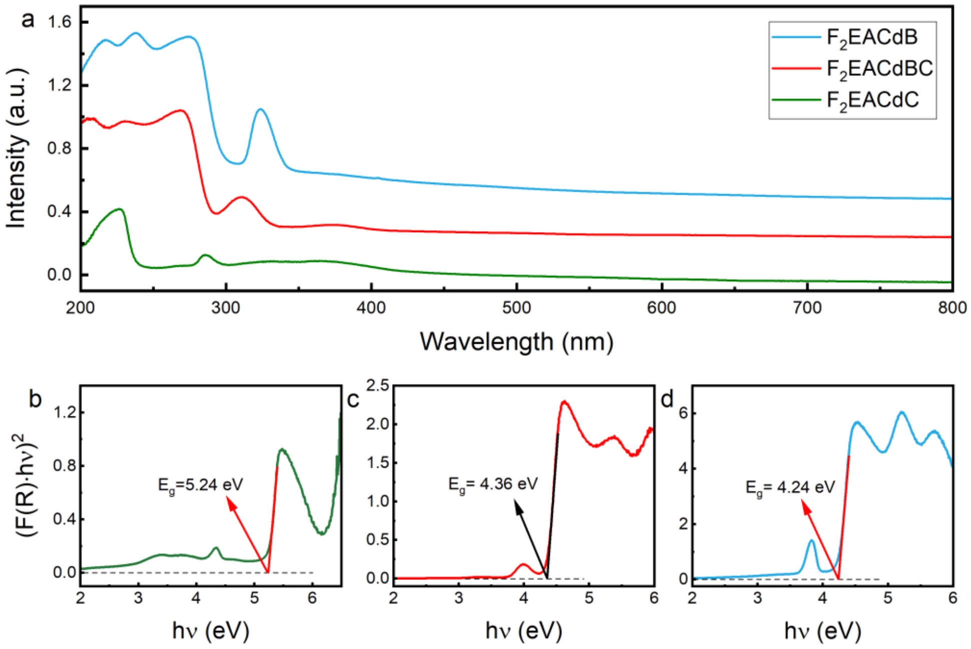


**Figure S25**. Diffuse reflectance spectra of investigated materials (a) and their energy band gap sizes (b-d).

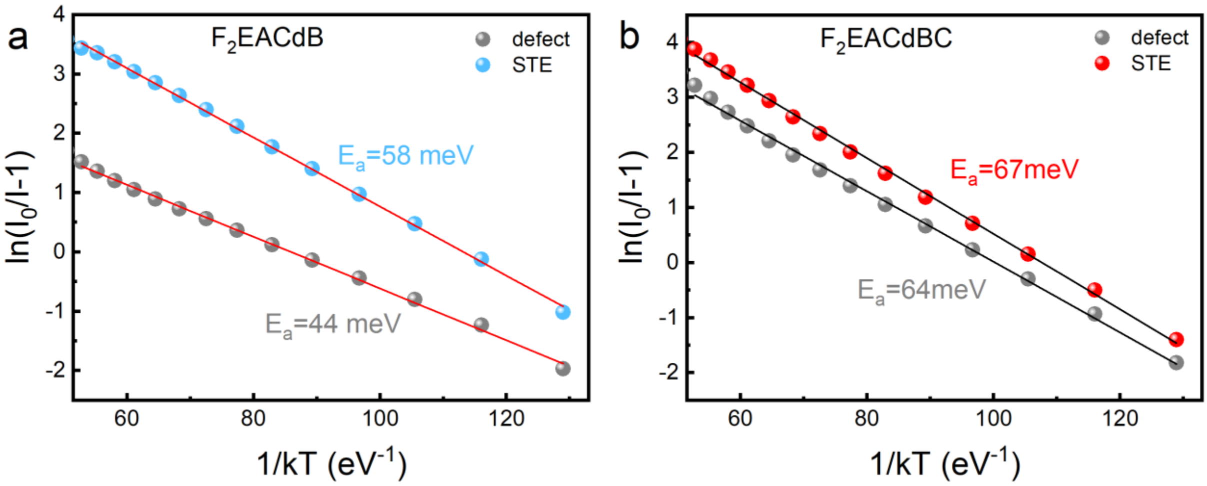


**Figure S26**. The activation energy of the thermal quenching of structural defect-related and STE emission bands of the **$F_2$EACdB** (a) and **$F_2$EACdBC** (b) crystals upon 266 nm excitation line.

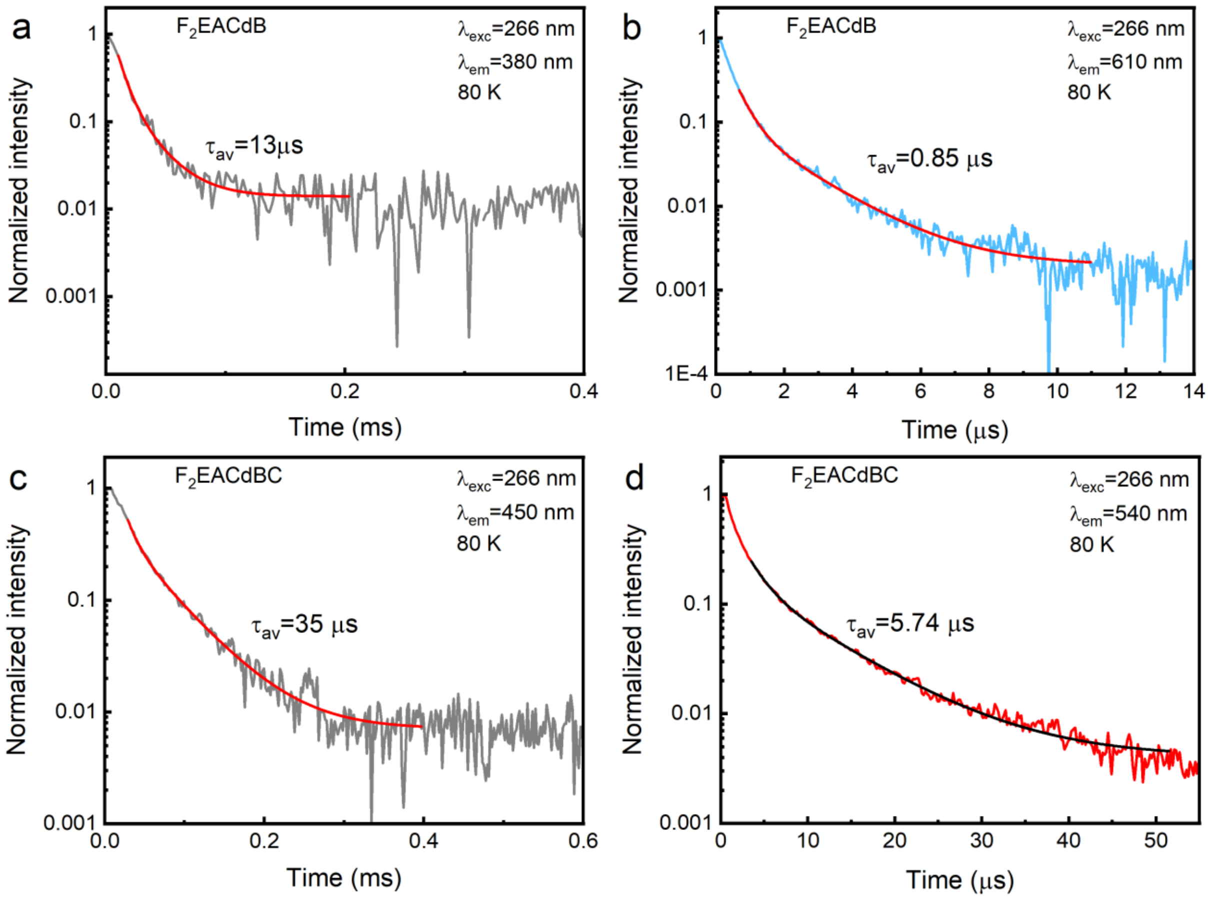


**Figure S27**. The luminescent decay profiles of the **$F_2$EACdB** (a, b) and **$F_2$EACdBC** (c, d) crystals measured for structural defect-related (a, c) and STE (b, d) emission at 80 K.
